# Facile approach to N,O,S-heteropentacycles via condensation of sterically crowded 3*H*-phenoxazin-3-one with *ortho-*substituted anilines

**DOI:** 10.3762/bjoc.20.34

**Published:** 2024-02-21

**Authors:** Eugeny Ivakhnenko, Vasily Malay, Pavel Knyazev, Nikita Merezhko, Nadezhda Makarova, Oleg Demidov, Gennady Borodkin, Andrey Starikov, Vladimir Minkin

**Affiliations:** 1 Institute of Physical and Organic Chemistry, Southern Federal University, 194/2 Stachki St., 344090, Rostov-on-Don, Russian Federation; 2 North Caucasus Federal University, 1 Pushkin St., 355017, Stavropol, Russian Federation

**Keywords:** 3*H*-phenoxazin-3-one, fluorescence, molecular structure, pentacyclic heterocycles, synthesis

## Abstract

A convenient method for the synthesis of a series of 2-(arylamino)-3*H*-phenoxazin-3-ones based on the nucleophilic substitution reaction between sterically crowded 3*H*-phenoxazin-3-one and arylamines performed by short-term heating of the melted reactants at 220–250 °C is described, and the compounds were characterized by means of single-crystal X-ray crystallography, NMR, UV–vis, and IR spectroscopy, as well as cyclic voltammetry. The reaction with *o*-amino-, *o*-hydroxy-, and *o*-mercapto-substituted arylamines widened the scope and provided an access to derivatives of N,O- and N,S-heteropentacyclic quinoxalinophenoxazine, triphenodioxazine and oxazinophenothiazine systems.

## Introduction

**Scheme 1:**
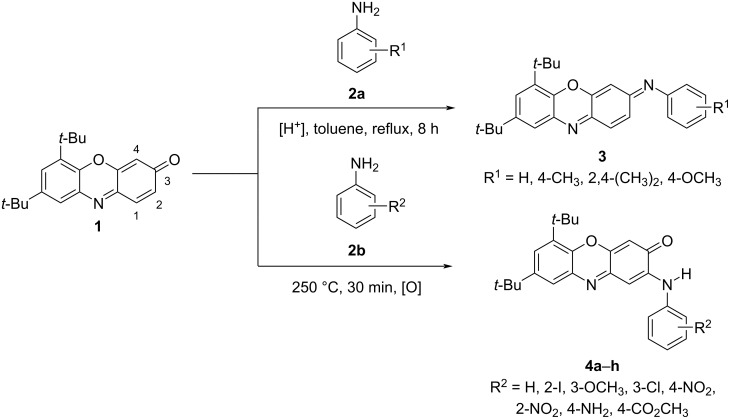
Synthesis of 6,8-di-*tert*-butyl-*N*-aryl-3*H*-phenoxazin-3-imines **3** [6] and 6,8-di-*tert*-butyl-2-(arylamino)-3*H*-phenoxazin-3-ones **4**.

**Figure 1:**
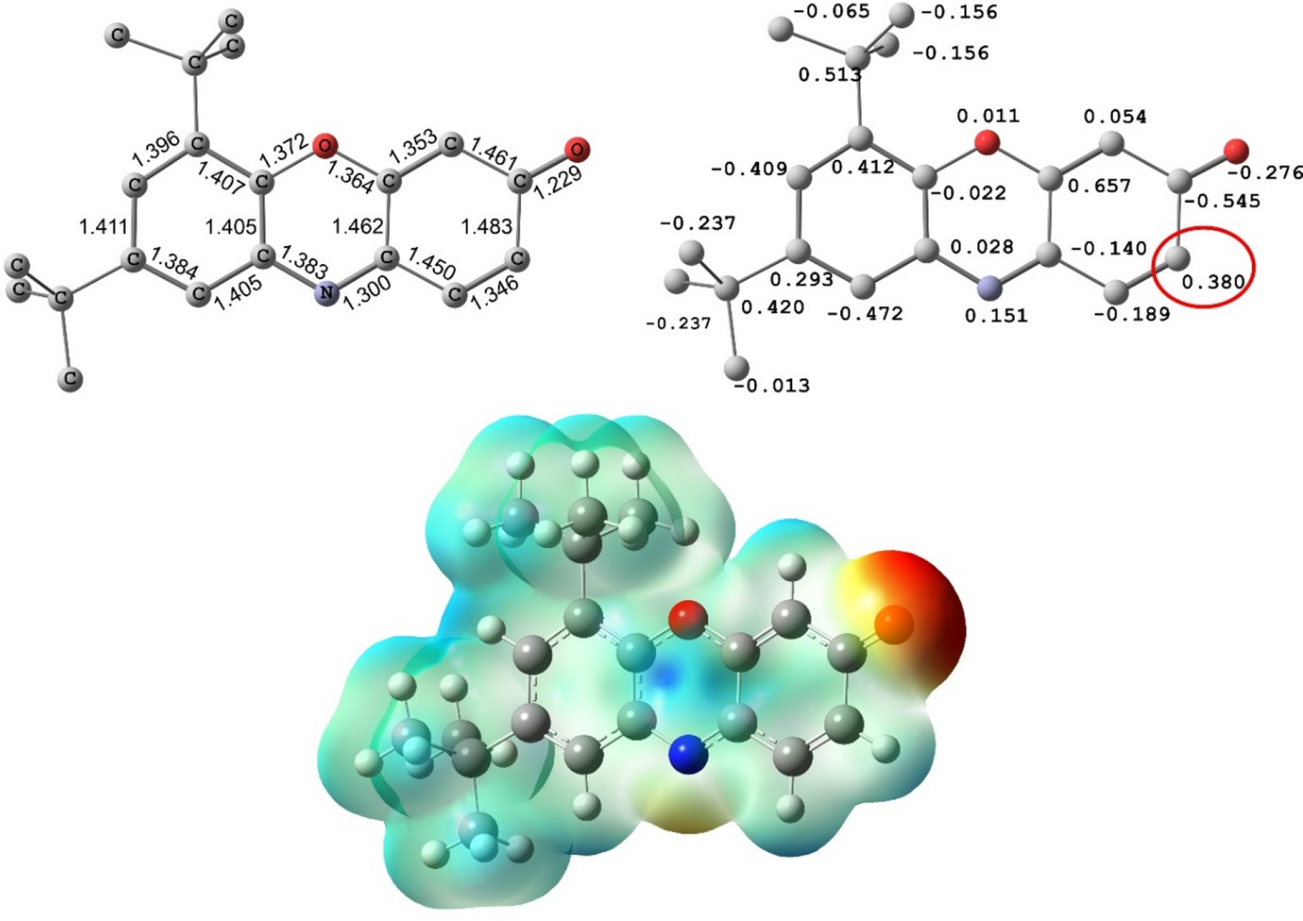
DFT-calculated molecular geometry (B3LYP/6-311++G(d,p) level) and distribution of electronic density in 6,8-di-*tert*-butyl-3*H*-phenoxazin-3-one (**1**): Mulliken charges and molecular electrostatic potential (MEP, isovalue = 0.004).

3*H*-Phenoxazin-3-one and its derivatives are widely distributed in nature in microorganisms and fungi, and they represent the key structural units of many important drugs with antibacterial, antifungal, anticancer, anti-inflammatory, and antiviral activities [1–2]. Due to the presence of several reactive centers in the structure, 3*H*-phenoxazin-3-ones can easily be accessed through oxidative couplings of *o*-aminophenols [3–4] or *N*-aryl-*o*-benzoquinone imines [5–6]. Further, they can serve as efficient precursors of pentacyclic N,O-heterocyclic compounds that possess promising properties for application in fluorescent probes, organic light-emitting diodes, and organic solar cells [2,7–9]. The principal way for the preparation of these heterocycles involves the coupling of 3*H*-phenoxazin-3-ones with variously functionalized aromatic amines. This is followed by the cyclization of the initially formed adducts [10–12]. At the first stage, this reaction follows one of three possible reaction pathways, including Schiff base formation (attack at the C(3) center), Michael addition at C(1), or nucleophilic substitution (S_N_H) at the C(2) center [13–15]. Most readily used is the pathway involving carbonyl–amine condensation and Schiff base formation, which is then cyclized [12,16]. The reaction of **1** with arylamines **2a** is performed in toluene solution in the presence of a catalytic amount of *p*-toluenesulfonic acid. This readily affords 6,8-di-*tert*-butyl-*N*-aryl-3*H*-phenoxazin-3-imines **3** but proceeds smoothly only with highly basic amines (Scheme 1) [6]. The choice for one of the other two possible reaction pathways (nucleophilic additions to either the C(1) or C(2) center) critically depends on the electrophilicity. Figure 1 shows the distribution of electronic density in 6,8-di-*tert*-butyl-3*H*-phenoxazin-3-one (**1**). This is also the basic compound used in the transformations that are studied in this work due to the high kinetic stability and good solubility ensured by the *tert*-butyl groups. The largest positive charge of the C(1)–C(2)–C(3) segment is concentrated at the C(2) atom. The charge at the other electrophilic center C(1) of **1** is much lower.

It comes, therefore, with no surprise that the interaction of arylamines and the 5-hydroxy and 5-acetoxy derivatives of 3*H*-phenoxazin-3-one is directed toward the C(2) reaction center to yield 2-amino-3*H*-phenoxazin-3-ones as the final products under aerobic conditions. The reactions proceed readily in refluxing acidified (p*K*_a_ = 1–5) ethanol solutions of the amine hydrochlorides to give 2-monosubstituted derivatives of 3*H*-phenoxazin-3-ones in a moderate yield [10,17]. In the present work, we intended to explore the reaction of 3*H*-phenoxazin-3-ones with aromatic amines, the direction of which is controlled by the large positive charge at the C(2) center of the *p*-quinone imine moiety of the heterocycle. With this in mind, we turned our attention to solid-state organic reactions. Numerous examples of nucleophilic substitutions at carbon centers are discussed in comprehensive reviews [18–19], but none is directly related to aromatic S_N_H reactions. The developed procedure was applied to the synthesis of compounds **4** and extended to arylamines with *o*-amino, *o*-hydroxy, and *o*-mercapto substituents, providing access to N-, O-, and S-containing heteropolycyclic structures.

## Results and Discussion

**Scheme 2:**
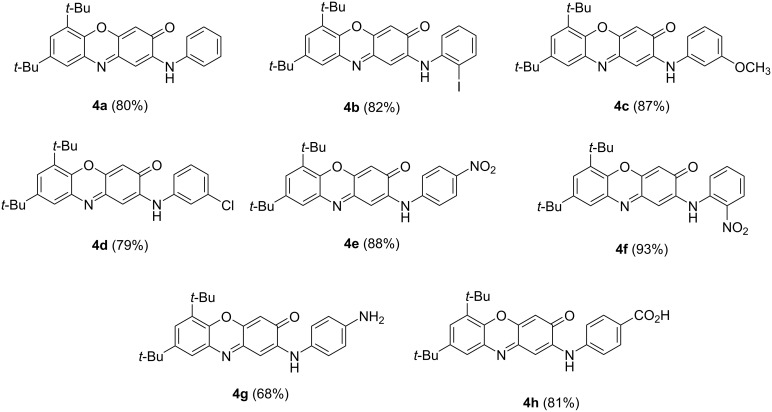
6,8-Di-*tert*-butyl-2-(arylamino)-3*H*-phenoxazin-3-ones **4** prepared by the one-pot reaction between 6,8-di-*tert*-butyl-3*H*-phenoxazin-3-one (**1**) and aromatic amines **2b** (the yield is given in parentheses).

We found that a convenient way toward 6,8-di-*tert*-butyl-2-(arylamino)-3*H*-phenoxazin-3-ones **4** involves the short-term heating (30 min) of a molten mixture of **1** and an arylamine at 250 °C, followed by purification of the products by column chromatography. No preliminary grinding of the crystalline samples, which is otherwise typical for solid-state reaction, was employed in this case. As seen in Scheme 2, the nucleophilic substitution reaction occured in good yield and with no restrictions in terms of amine basicity.

**Figure 2:**
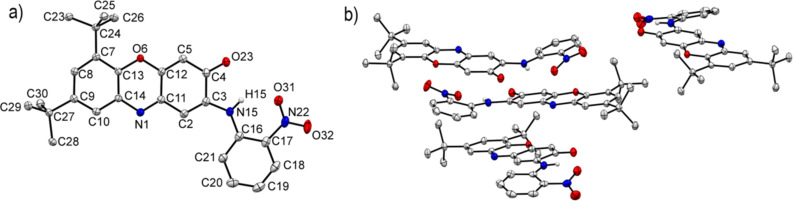
Molecular structure of 6,8-di-*tert*-butyl-2-(*o*-nitrophenylamino)-3*H*-phenoxazin-3-one (**4f**). a) Selected bond distances (Å) and angles: N(1)–C(11) 1.3061(19), N(1)–C(14) 1.3836(18), O(23)–C(4) 1.2314(19), N(15)–C(3) 1.3765(19), N(15)–C(16) 1.383(2), C(11)–N(1)–C(14) 117.55(12), C(3)–N(15)–C(16) 131.12(14). b) Crystal packing of **4f**. Important crystallographic parameters and bond distances are given in Tables S2 and S5, Supporting Information File 1. Thermal ellipsoids are drawn at the 50% probability level.

The molecular structures of compounds **4c**,**d**,**f** were determined by X-ray crystallography and are shown in Figure 2 (i.e., **4f**) and Figures S1 and S2, Supporting Information File 1 (i.e., **4c**,**d**). The geometry of the phenoxazine-3-one fragment of **4c**,**d**,**f** coincides with that found for 6,8-di-*tert*-butyl-3*H*-phenoxazin-3-one (**1**) [6]. A strong hydrogen bridge, N(15)–H···O(31), is formed between the nitro and imino groups of the *N*-aryl ring.

**Figure 3:**
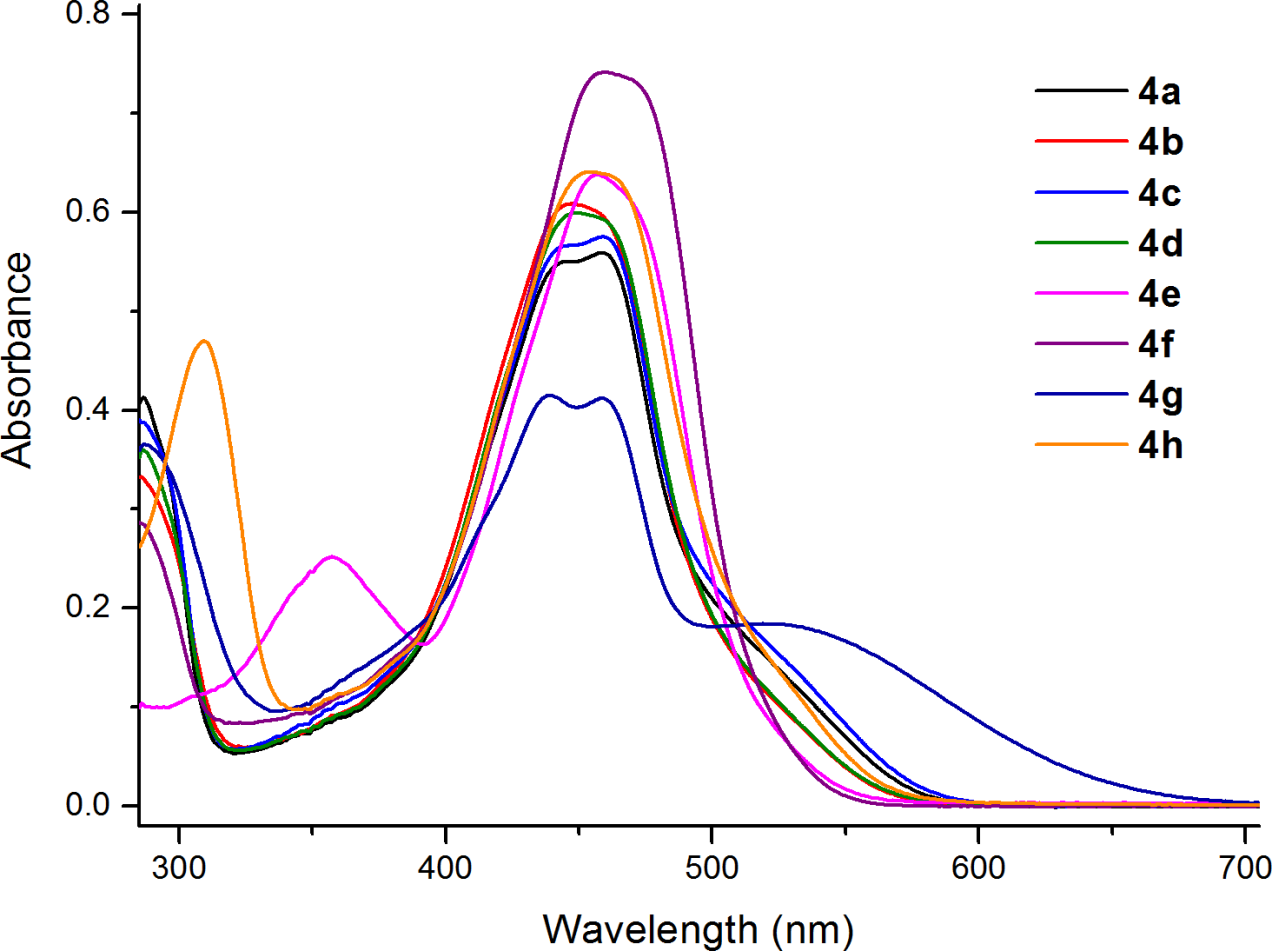
UV–vis spectra of 6,8-di-*tert*-butyl-2-(arylamino)-3*H*-phenoxazin-3-ones **4a**–**h** (toluene, *c* = 2⋅10^−5^ M, *l* = 1 cm, *T* = 293 K).

The compounds **4a**–**h** intensely absorb light in the spectral range of 400–550 nm, with maxima at 439–459 nm, ε = 20600–37100 M^−1^⋅cm^−1^ (Figure 3 and Table 1). The introduction of an amino group into the *p*-position of the *N*-phenyl fragment gave rise to the appearance of an additional long-wavelength absorption band with λ_max_ = 520 nm and ε = 9200 M^−1^⋅сm^−1^.

**Table 1:** UV–vis absorption data of 6,8-di-*tert*-butyl-2-(arylamino)-3*H*-phenoxazin-3-ones **4a**–**h** in toluene.

compound	λ_max_, nm (ε, 10^4^ M^−1^·cm^−1^)

**4a**	445 (2.75)^a^, 458 (2.80)
**4b**	447 (3.04)
**4c**	444 (2.83)^a^, 459 (2.88)
**4d**	449 (2.99)
**4e**	357 (1.26), 457 (3.19)
**4f**	459 (3.71)
**4g**	439 (2.07), 459 (2.06), 520 (0.92)
**4h**	309 (2.35), 454 (3.20)

^a^Shoulder.

**Scheme 3:**
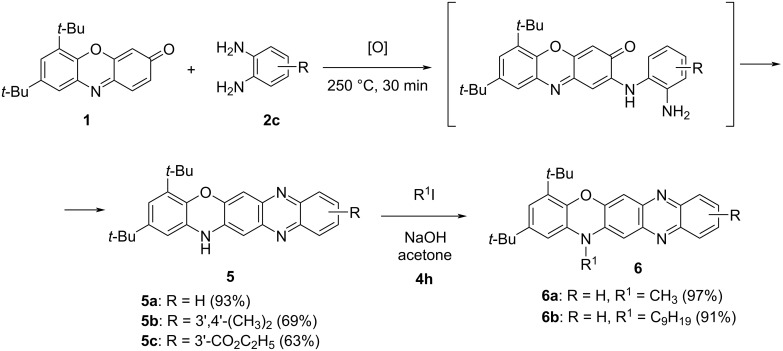
Synthesis of 14*H*-quinoxaline[2,3-*b*]phenoxazines **5** and **6**.

Subjecting *o*-phenylenediamines **2с** to the reaction with 3*H*-phenoxazin-3-one makes the simultaneous activation of two principle reaction pathways (S_N_H and Schiff base formation) possible. By using a similar procedure to that applied to the synthesis of compounds **4**, we succeeded in the preparation of 14*Н*-quinoxaline[2,3-*b*]phenoxazine derivatives **5** (Scheme 3).

**Scheme 4:**
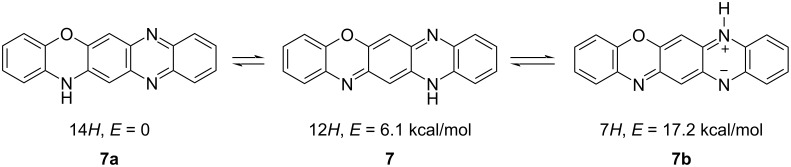
Relative stability of the tautomers **7** and **7a**,**b** of quinoxaline[2,3-*b*]phenoxazine calculated at the DFT B3LYP/6-311++G(d,p) level.

The nitrogen atoms in the oxazine and pyrazine rings of **5**, N(7), N(12), and N(14), offer three possible positions for the N–H proton. Therefore, three tautomeric forms are possible for **5** (Scheme 4), one of which, the 7*H*-tautomer **7b**, inevitably adopts a bipolar or biradical structure. According to the data from the DFT calculations performed at the B3LYP/6-311++G(d,p) approximation (Figure S6, Supporting Information File 1), the energetically preferred tautomer is the 14*H*-form **7a**. The least stable 7*H*-isomer **7b** conforms to a minimum on the corresponding potential energy surface. However, the stable wave function of **7b** corresponds to an electronic state with a broken symmetry [20], indicating the presence of two unpaired electrons and the singlet biradical form.

**Scheme 5:**
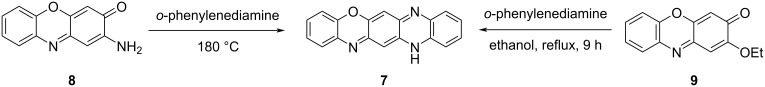
Preparation of quinoxaline[2,3-*b*]phenoxazine (**7**) from 2-amino-3*H*-phenoxazin-3-one (**8**) [10] and 2-ethoxy-3*H*-phenoxazin-3-one (**9**) [11], respectively.

In previous studies on the coupling of 3*H*-phenoxazin-3-one derivatives **8** and **9** with *o*-phenylenediamine [10–11], the preference was given to the 12*Н*-quinoxaline[2,3-*b*]phenoxazine form **7** (Scheme 5). A series of *N*-aryl derivatives of this form was also obtained via treatment of 6,8-di-*tert*-butyl-*N*-aryl-3*H*-phenoxazin-3-imines with various arylamines in the presence of an excess of trifluoroacetic acid [9].

**Figure 4:**
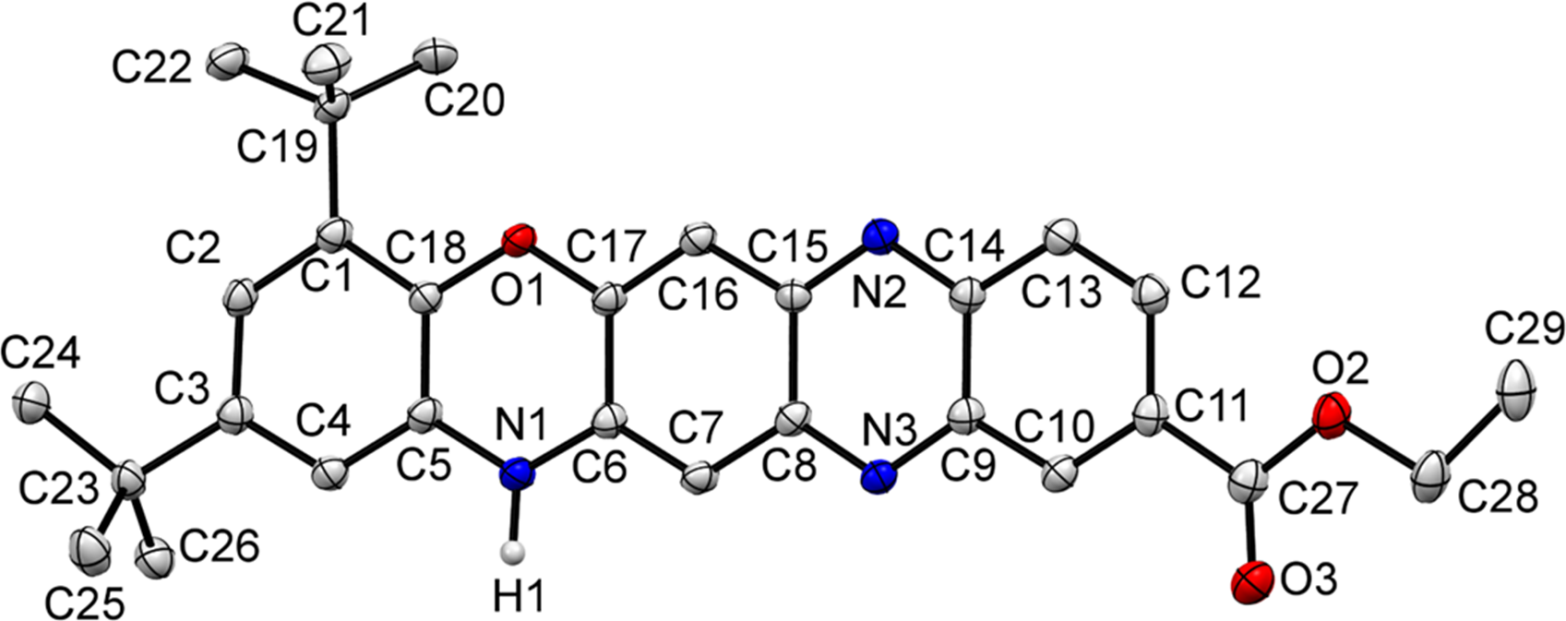
Molecular structure of ethyl 2,4-di-*tert*-butyl-14*H*-quinoxalino[2,3-*b*]phenoxazine-10-carboxylate (**5c**), with atom numbering scheme. Selected bond distances (Å) and angles: N(1)–C(5) 1.390(2), N(1)–C(6) 1.365(3), N(2)–C(14) 1.363(3), N(2)–C(15) 1.336(3), N(3)–C(8) 1.338(3), N(3)–C(9) 1.356(3), C(6)–N(1)–C(5) 122.13(17), C(15)–N(2)–C(14) 116.88(17), C(8)–N(3)–C(9) 116.66(17). All bond lengths, angles, and important crystallographic parameters are given in Tables S6 and S7, Supporting Information File 1. Hydrogen atoms are omitted for clarity.

The structure of the newly synthesized compounds **5**, which are derivatives of a previously unknown 14*Н*-quinoxaline[2,3-*b*]phenoxazine system **7a**, was unambiguously established based on COSY, HSQC, and HMBC NMR-spectroscopic data. Further, the ^15^N NMR spectrum of **5a** confirmed the typical pyrrole-like character of the N(12) atom as well as the pyridine-like character of the N(7) and N(14) atoms (Figure S31, Supporting Information File 1) [21–22]. The molecular structure of **5c** was also determined using X-ray crystallography (Figure 4).

**Scheme 6:**
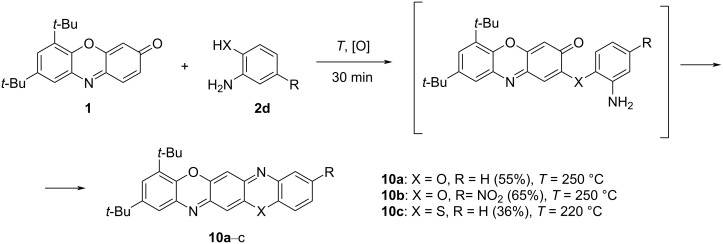
Triphenodioxazine and oxazinophenothiazine derivatives **10** via condensation of 3*H*-phenoxazin-3-one **1** with *o*-aminophenol and *o*-mercaptoaniline derivatives **2d**.

We assumed that the scope of the reaction shown in Scheme 3 could be expanded via replacement of one of the amino groups of *o*-phenylenediamine by another strong nucleophilic center. It was earlier found [23] that condensation of 3*H*-phenoxazin-3-one (**1**) with various *o*-aminophenols (in refluxing DMF for 8–10 h), upon formation of the corresponding imine intermediate, affords benzo[5,6][1,4]oxazino[2,3-*b*]phenoxazines derivatives **10a**,**b** (triphenodioxazines). As shown in the present work, this reaction can also be performed successfully under the conditions applied to the preparation of 14*H*-quinoxaline[2,3-*b*]phenoxazines **5**. The reaction proceeds readily with *o*-mercaptoaniline to produce the benzo[5,6][1,4]oxazino[2,3-*b*]phenothiazine derivative **10c** (Scheme 6).

**Figure 5:**
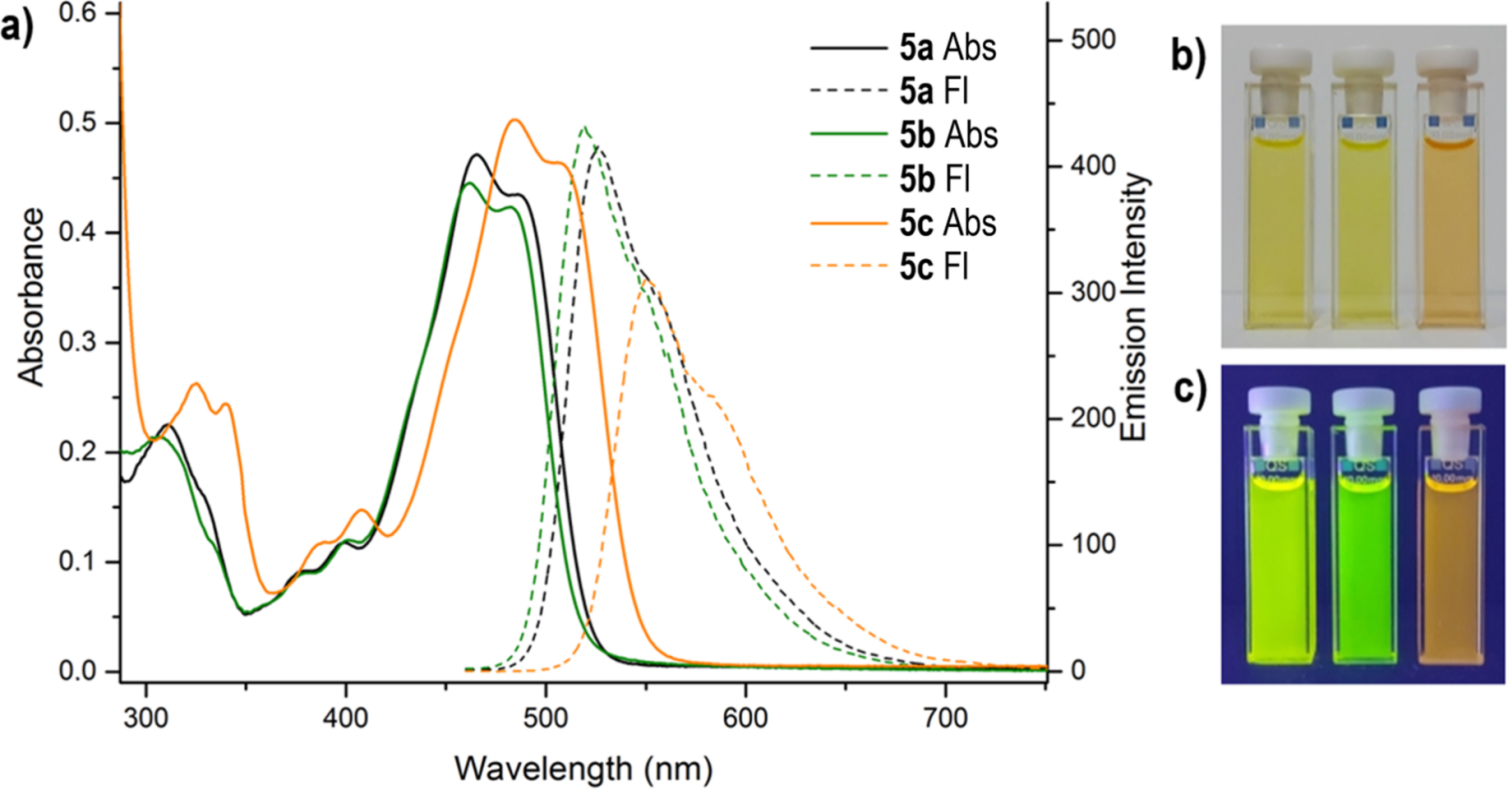
a) UV–vis (solid lines) and fluorescence emission (λ_ex_ = 365 nm, dashed) spectra of compounds **5a**–**c** (toluene, *c* = 2 10^−5^ M, *l* = 1 cm). b) Solutions of compounds **5a**–**c** in toluene before irradiation (no emission) and c) during irradiation (photoluminescence, λ_ex_ = 365 nm) at room temperature.

**Figure 6:**
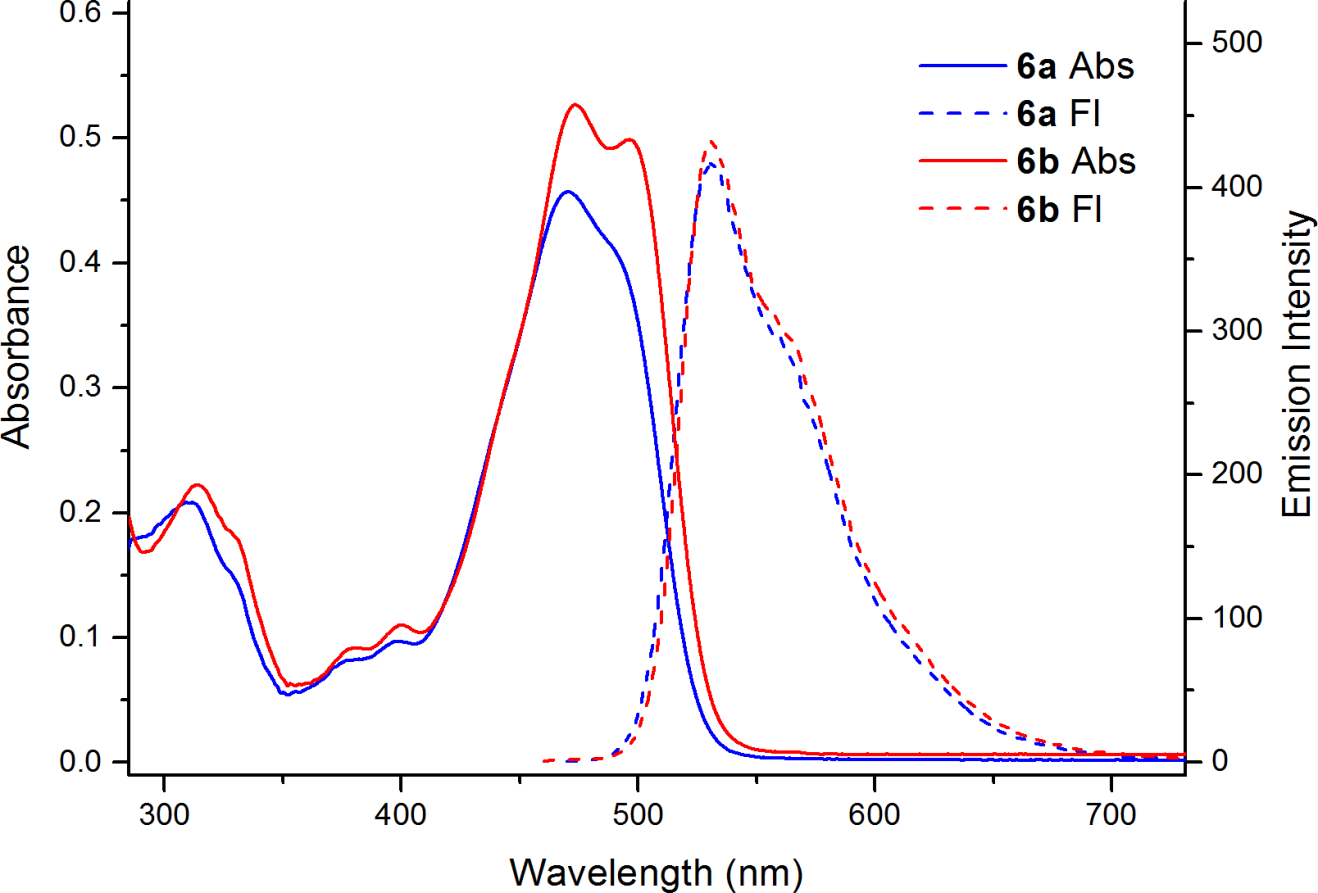
UV–vis (solid lines) and fluorescence emission (dashed, λ_ex_ = 365 nm) spectra of compounds **6a**,**b** in toluene (*c* = 2 10^−5^ M, *l* = 1 cm) at room temperature.

**Figure 7:**
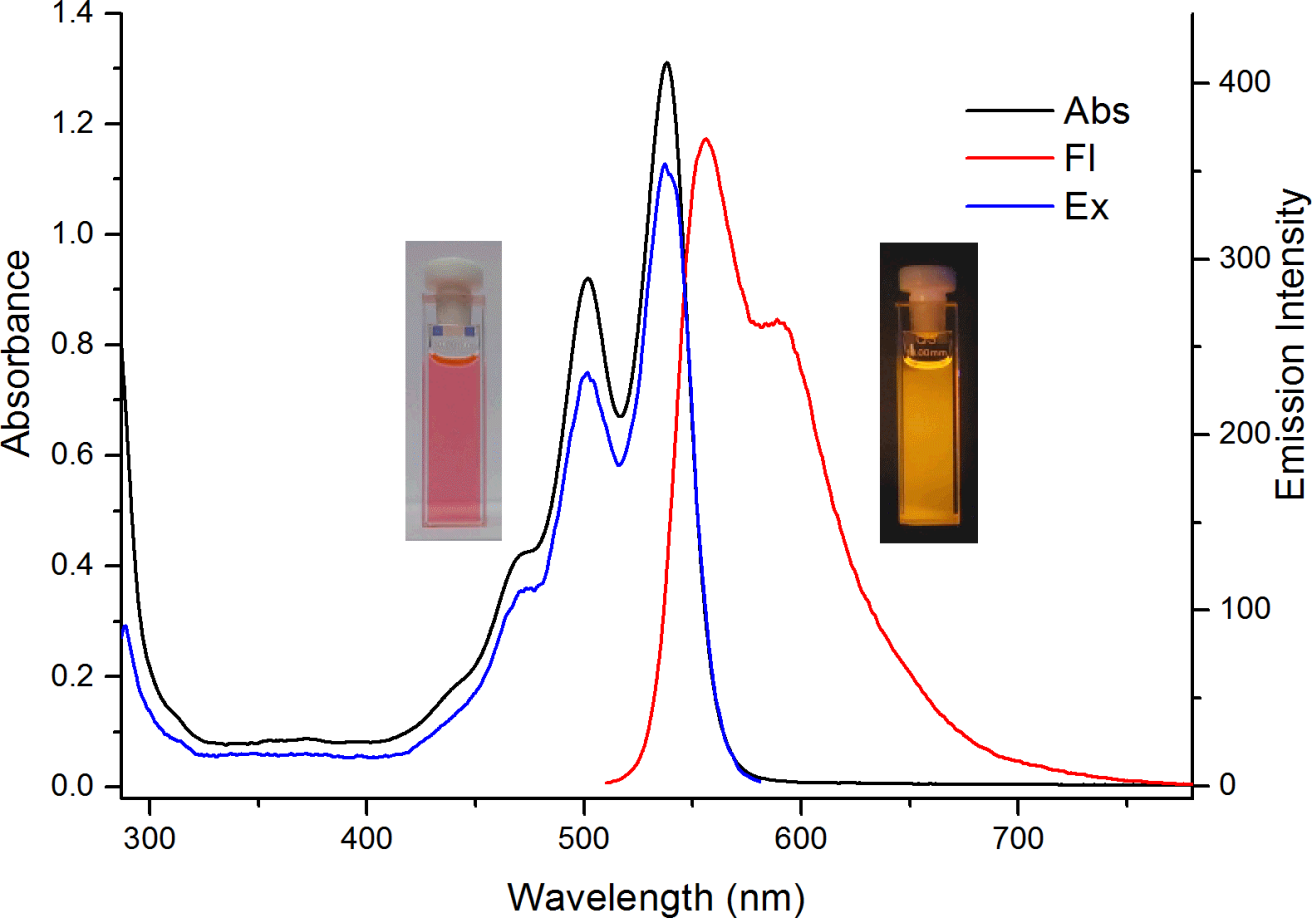
UV–vis, fluorescence emission (λ_ex_ = 500 nm), and fluorescence excitation (λ_obs_ = 590 nm) spectra of benzo[5,6][1,4]oxazino[2,3-*b*]phenothiazine **10c** in toluene (*c* = 2 10^−5^ M (UV–vis) or *c* = 2 10^−6^ M (fluorescence), *l* = 1 cm) at room temperature.

Electronic absorption spectra of the prepared 14*Н*-quinoxaline[2,3-*b*]phenoxazines **5** and **6** (Table 2, Figures 5–7, and Figures S7–S11, Supporting Information File 1) exhibit broad and high-intensity absorption maxima in the range of 450–550 nm, which encompass the strongest emissive part of the solar spectrum. In contrast to nonfluorescent 6,8-di-*tert*-butyl-2-(arylamino)-3*H*-phenoxazin-3-ones **4**, compounds **5** and **6** display intense fluorescence in solution at room temperature (Figure 5). The excitation spectra of the compounds (Figures S7–S11, Supporting Information File 1) correspond to the longest-wavelength absorption bands. The absorption and emission spectra of benzo[5,6][1,4]oxazino[2,3-*b*]phenothiazine **10c** (Figure 7) were bathochromically shifted by about 50 nm relative to those of the quinoxaline[2,3-*b*]phenoxazines **6**.

**Table 2:** UV–vis absorption and fluorescence emission data of compounds **5a**–**c**, **6a**,**b**, and **10c** in toluene.

compound	absorption λ_max_, nm (ε, 10^4^ M^−1^⋅cm^−1^)	emission λ_fl_, nm	Φ_fl_^a^

**5a**	311 (1.13), 327^b^ (0.83), 380^b^ (0.46), 400 (0.59), 466 (2.36), 486^b^ (2.18)	526, 550^b^	0.19
**5b**	307 (1.07), 326^b^ (0.72), 379^b^ (0.45), 401^b^ (0.60), 462 (2.23), 482 (2.12)	519, 542^b^	0.20
**5c**	325 (1.32), 339 (1.22), 389^b^ (0.59), 408 (1.47), 484 (2.52), 505^b^ (2.32)	550, 573^b^	0.13
**6a**	310 (1.04), 326^b^ (0.78), 379^b^ (0.41), 399 (0.49), 471 (2.29), 486^b^ (2.10)	531, 555^b^	0.19
**6b**	314 (1.11), 327^b^ (0.93), 380^b^ (0.46), 400 (0.55), 473 (2.64), 497 (2.50)	531, 555^b^	0.19
**10c**	474^b^ (2.13), 502 (4.61), 538 (6.56)	556, 590^b^	0.35

^a^Fluorescence quantum yield. ^b^Shoulder.

The electrochemical behavior of compounds **4a**–**h**, **5a**–**c**, **6a**,**b**, and **10c** was studied using cyclic voltammetry (CV). As exemplified by the CV curves (Figure S12, Supporting Information File 1), 2-(arylamino)-3*H*-phenoxazin-3-ones **4a**–**h** manifest two reduction waves at *Е*_1/2_^RED1^ = −1.36 ± 1.69 V and *Е*_1/2_^RED2^ = −1.85 ± 2.12 V. Oxidation of **4a**–**f**,**h** occurs as an irreversible process at *Е*_1/2_^OX^ = 0.81–1.07 V. For **4g** bearing an amino group, the oxidation potential is shifted to *Е*_1/2_^OX^ = 0.25 V. The irreversible two-wave reduction (*Е*_1/2_^RED1^ = −1.40 ± 1.60 V and *Е*_1/2_^RED2^ = −1.92 ± 2.45 V) is also characteristic of 14*H*-quinoxaline[2,3-*b*]phenoxazines **5a**–**c** and **6a**,**b**. In contrast to **5a**–**c** and **6a**,**b**, benzo[5,6][1,4]oxazino[2,3-*b*]phenothiazine **10c** is reversibly reduced at *Е*_1/2_^RED1^ = −1.39 V to a radical anion and then undergoes irreversible reduction at *Е*_1/2_^RED2^ = −1.91 V and irreversible oxidation at *Е*_1/2_^OX^ = 0.48 V. These CV parameters are close to those recorded for triphenodioxazines [23]. The energy of the HOMO and LUMO orbitals assessed on the basis of the CV and electronic absorption spectral data are given in Table 3.

**Table 3:** CV parameters and calculated energy levels of **4a**–**h**, **5a**–**c**, **6a**,**b**, and **10c**.

compound	CV (vs Fc^+^/Fc)						UV–vis	
	
	*E*_1/2_^OX^, V	*E*_1/2_^RED1^, V	*E*_1/2_^RED2^, V	HOMO, eV	LUMO, eV	∆*E*, eV	∆*E*, eV	LUMO, eV

**4a**	0.89	−1.41	−1.97	−5.69	−3.39	2.30	2.71	−2.98
**4b**	0.91	−1.36	−1.90	−5.71	−3.44	2.27	2.77	−2.94
**4c**	0.83	−1.42	−1.92	−5.63	−3.38	2.25	2.70	−2.93
**4d**	0.81	−1.36	−1.94	−5.61	−3.44	2.17	2.76	−2.85
**4e**	1.07	−1.59	−1.91	−5.87	−3.21	2.66	2.71	−3.16
**4f**	1.05	−1.69	−2.12	−5.85	−3.11	2.74	2.70	−3.15
**4g**	0.25	−1.54	−1.96	−5.05	−3.26	1.79	2.38	−2.67
**4h**	0.92	−1.43	−1.85	−5.72	−3.37	2.35	2.73	−2.99
**5a**	0.86	−1.40	−2.32	−5.66	−3.40	2.26	2.55	−3.11
**5b**	0.95	−1.60	−2.45	−5.75	−3.20	2.55	2.57	−3.18
**5c**	0.82	−1.40	−2.15	−5.62	−3.40	2.22	2.46	−3.16
**6a**	1.10	−1.45	−1.92	−5.9	−3.35	2.55	2.55	−3.35
**6b**	1.15	−1.48	−2.02	−.95	−3.32	2.63	2.49	−3.46
**10c**	0.48	−1.39	−1.91	−.28	−3.41	1.87	2.30	−2.98

## Conclusion

The diverse reactions of 3*H*-phenoxazin-3-one derivatives with nucleophilic reagents are primarily directed toward the *p*-quinone imine fragment [5,13,15]. In the presence of protonic acids, the reaction with amines proceeds through Schiff base formation [6]. In turn, without an acidic catalyst, it is driven by the distribution of the electron density (Figure 1), such that the nucleophilic attack occurs at the most electrophilic C(2) center. With this in mind, we presented a convenient procedure for the S_N_H reaction of aromatic amines with sterically crowded 6,8-di-*tert*-butyl-3*H*-phenoxazin-3-one (**1**) that afforded a series of 6,8-di-*tert*-butyl-2-(arylamino)-3*H*-phenoxazin-3-ones **4** prepared in 68–93% yield (Scheme 2). Using *o*-amino-, *o*-hydroxy- and *o*-mercapto-substituted anilines in this process allowed to pursue both principal reaction pathways (Schiff base formation and S_N_H), which led to the formation of derivatives of the previously unknown 14*Н*-quinoxaline[2,3-*b*]phenoxazine system **5** (Scheme 3) as well as N,O- and N,S-heteropentacyclic triphenodioxazines and oxazinophenothiazine **10a**–**c**. The structural assignment [10–11] of the N,O-containing reaction products as 12*H*-quinoxaline[2,3-*b*]phenoxazines was confirmed through DFT calculations, X-ray crystallography, and NMR spectroscopy.

Electronic absorption spectra (Table 2 and Figures 5–7) and electrochemical properties (Table 3) of the heteropentacyclic compounds **4a**–**h**, **5a**–**c**, **6a**,**b**, and **10c** revealed potential for testing as potential donors for organic solar cells or as dye sensitizers for dye-sensitized solar cells [24–25].

## Experimental

All reagents and solvents were purchased from commercial sources (Aldrich) and used without additional purification. The compounds were characterized by ^1^H, ^13^C, and ^15^N NMR spectroscopy (NMR spectra of compounds **4a**–**h**, **5a**–**c**, **6a**,**b**, and **10c** are given in Figures S13–S43, Supporting Information File 1), mass spectrometry (Figures S44–S56, Supporting Information File 1), IR and UV–vis spectroscopy, as well as elemental analysis. The NMR spectra were recorded on the spectrometers Varian UNITY-300 (300 MHz for ^1^H) and Bruker AVANCE-600 (600 MHz for ^1^H, 151 MHz for ^13^C, and 60 MHz for ^15^N) in CDCl_3_ solutions. Chemical shifts are reported in ppm using the residual solvent peaks as reference (7.24 ppm for ^1^H, 77.0 ppm for ^13^C, and 384 ppm for ^15^N using nitromethane). Chemical shifts were measured with a precision of 0.01 ppm, and 0.1 Hz for spin–spin coupling constants *J*. The assignment of resonance peaks was carried out using COSY, HSQC, and ^1^H,^13^C as well as ^1^H,^15^N HMBC. Melting points were determined using a PTP (M) apparatus and were left uncorrected. IR spectra were recorded on a Varian Excalibur 3100 FTIR instrument using the attenuated total internal reflection technique (ZnSe crystal). UV–vis spectra were recorded at *c* = 2⋅10^−5^ M in toluene solutions with a Varian Cary 100 spectrophotometer. Photoluminescent spectra were recorded at *c* = 2⋅10^−5^ M (compounds **5a**–**c** and **6a**,**b**) and *c* = 2⋅10^−6^ M (compound **10c**) in toluene solutions with a Varian Cary Eclipse fluorescence spectrophotometer. UV–vis and fluorescence spectra were recorded using standard 1 cm quartz cells at room temperature. Toluene of spectroscopic grade (Aldrich) was used to prepare the solutions. The fluorescence quantum yield was determined relative to quinine bisulfate in 0.05 M H_2_SO_4_ as standard (Φ_F_ = 0.52, excitation at 365 nm for **5a**–**c** and **6a**,**b**) [26] and cresyl violet perchlorate in ethanol (Φ_F_ = 0.54, excitation at 510 nm for **10c**) [27]. Mass spectrometric analysis was performed on a Bruker UHR-TOF Maxis™ Impact (resolving power (FWHM) of 40,000 at *m*/*z* 1222, electrospray ionization). The cyclic voltammograms of **4a**–**h**, **5a**–**c**, **6a**,**b**, and **10c** were measured with the use of three-electrode configuration (glassy carbon working electrode, Pt counter electrode, Ag/Ag^+^ reference electrode using 0.01 M AgNO_3_ in CH_3_CN) in CH_2_Cl_2_ (**4a**–**h**), CH_3_CN (**5a**–**c**, **6a**,**b**, and **10c**) and potentiostat–galvanostat Elins P-45X. X-ray data collection was performed on an Agilent SuperNova diffractometer using a microfocus X-ray source with copper anode (Cu Kα radiation, λ = 1.54184 Å) and Atlas S2 CCD detector. The diffraction data of **4c**,**d**,**f**, **5c**, and **10b** were obtained at 100 K. Crystals of **5c** were obtained in the form of a solvate with molecules of isopropanol and water present. The protons attached to heteroatoms were localized by difference Fourier synthesis and refined with isotropic thermal parameters. The collection of reflexes as well as the determination and refinement of unit cell parameters were performed by using the specialized CrysAlisPro 1.171.38.41 software suite [28]. The structures were solved by using the SHELXT program [29]. Structural refinement was performed with the SHELXL program [30]. Molecular graphics were rendered and prepared for publication with the Olex2 version 1.3.0 software suite [31]. The complete X-ray diffraction datasets were deposited in the Cambridge Crystallographic Data Center (CCDC numbers 2292841, 2292840, 2292847, 2308520, and 2292848, Tables S2–S9, Supporting Information File 1). The DFT calculations [32] were performed using the Gaussian 16 program package [33] with the B3LYP functional [34] and the 6-311++G(d,p) basis set. The structures corresponding to minima on the potential energy surface and states with broken symmetry [20] were found through complete optimization of the geometry without imposing symmetry restrictions, followed by analyzing the stability of the DFT wave function. The images of the molecular structures in Figure 1 and Figure S6, Supporting Information File 1, were obtained using the Chemcraft program [35].

## Supporting Information

File 1 Synthetic details, compound characterization and additional analytic data, including copies of spectra and Cartesian coordinates.

## Data Availability

All data that supports the findings of this study is available in the published article and/or the supporting information to this article.
