# Supplementary material for: Facile approach to N,O,S-heteropentacycles via condensation of sterically crowded 3H-phenoxazin-3-one with ortho-substituted anilines
Source: Beilstein J Org Chem. 2024 Feb 21;20:336–45. doi: 10.3762/bjoc.20.34 (PMC10896220; doi:10.3762/bjoc.20.34)
Supplement: File 1 — Synthetic details, compound characterization and additional analytic data, including copies of spectra and Cartesian coordinates. [file Beilstein_J_Org_Chem-20-336-s001.pdf]

BEILSTEIN JOURNAL OF ORGANIC CHEMISTRY

Supporting Information

for

Facile approach to N,O,S-heteropentacycles via condensation of sterically crowded 3*H*-phenoxazin-3-one with *ortho*-substituted anilines

Eugeny Ivakhnenko, Vasily Malay, Pavel Knyazev, Nikita Merezhko, Nadezhda Makarova, Oleg Demidov, Gennady Borodkin, Andrey Starikov and Vladimir Minkin

Beilstein J. Org. Chem. **2024,** *20,* 336–345. doi:10.3762/bjoc.20.34

Synthetic details, compound characterization and additional analytic data, including copies of spectra and Cartesian coordinates

License and Terms: This is a supporting information file under the terms of the Creative Commons Attribution License (https://creativecommons.org/licenses/by/4.0). Please note that the reuse, redistribution and reproduction in particular requires that the author(s) and source are credited and that individual graphics may be subject to special legal provisions.
The license is subject to the *Beilstein Journal of Organic Chemistry* terms and conditions: (https://www.beilstein-journals.org/bjoc/terms)

Table of Contents

1. Synthesis S2
2. XRD study S6
3. DFT study S17
4. UV–vis and luminescence spectra S18
5. Cyclic voltammetry S21
6. NMR spectra S22
7. HRMS spectra S56
8. Cartesian coordinates S69

1. Synthesis

General procedure for the synthesis of compounds **4a**–**h**

309 mg (1 mmol) of 6,8-di-*tert*-butyl-3*H*-phenoxazin-3-one (**1**) powder was mixed with 3 mmol of the corresponding amine powder, placed in a round-bottom flask, and heated in a glycerin bath for 30 minutes at a temperature of 250°C, stirring the melt with a spatula every 10 minutes. For compounds **4a**–**h**, the reaction mixture was separated by column chromatography (Al_2O_3, eluent toluene), and the resulting fraction was recrystallized from diethyl ether.

6,8-Di-*tert*-butyl-2-(phenylamino)-3*H*-phenoxazin-3-one (**4a**)

Dark orange solid with m.p. 145-147 °C. Yield 80% (320 mg). Rf ~ 0.70. IR (neat, cm^{-1}): 3269 (N-H), 2950 (t-Bu), 2903 (t-Bu), 2863 (t-Bu), 1566 (C=O), 1519 (C=N), 1266 (C-N), 1239 (C-N), 1182 (C-O), 1159 (C-O). ^1H NMR (600 MHz, $CDCl_3$) δ 7.71 (s, 1H), 7.63 (d, *J* = 2.1 Hz, 1H), 7.49 (d, *J* = 2.1 Hz, 1H), 7.37 (t, *J* = 7.8 Hz, 2H), 7.33 (d, *J* = 7.8 Hz, 2H), 7.12 (t, *J* = 7.8 Hz, 1H), 7.00 (s, 1H), 6.52 (s, 1H), 1.51 (s, 9H), 1.36 (s, 9H). ^{13}C NMR (151 MHz, $CDCl_3$) δ 180.08, 149.34, 147.85, 147.53, 141.98, 139.45, 138.94, 136.75, 134.20, 129.57, 125.33, 124.39, 123.81, 121.39, 103.20, 99.48, 35.17, 34.87, 31.40, 30.08. HRMS (ESI) m/z: $[M+H]^+$ Calcd. for $C_{26}H_{29}N_2O_2$ 401.2224; Found 401.2229.

6,8-Di-*tert*-butyl-2-((2-iodophenyl)amino)-3*H*-phenoxazin-3-one (**4b**)

Orange needles with m.p. 170-173 °C. Yield 82% (431 mg). Rf ~ 0.70. IR (neat, cm^{-1}): 3243 (N-H), 2961 (t-Bu), 2902 (t-Bu), 2868 (t-Bu), 1580 (C=O), 1529 (C=N), 1264 (C-N), 1208 (C-N), 1162 (C-O), 1150 (C-O). ^1H NMR (600 MHz, $CDCl_3$) δ 7.89 (d, *J* = 7.9, 1H), 7.78 (s, 1H), 7.62 (d, *J* = 2.3 Hz, 1H), 7.55 (d, *J* = 7.9 Hz, 1H), 7.50 (d, *J* = 2.3 Hz, 1H), 7.38 (t, *J* = 7.9 Hz, 1H), 6.89 (t, *J* = 7.9 Hz, 1H), 6.85 (s, 1H), 6.55 (s, 1H), 1.51 (s, 9H), 1.36 (s, 9H). ^{13}C NMR (151 MHz, $CDCl_3$) δ 179.74, 149.28, 147.88, 147.45, 141.81, 140.03, 139.86, 136.78, 134.08, 129.21, 126.17, 125.60, 123.83, 122.13, 103.33, 100.05, 93.67, 35.15, 34.84, 31.36, 30.03. HRMS (ESI) m/z: $[M+H]^+$ Calcd. for $C_{26}H_{28}IN_2O_2$ 527.1132; Found 527.1135.

6,8-Di-*tert*-butyl-2-((3-methoxyphenyl)amino)-3*H*-phenoxazin-3-one (**4c**)

Brown needles with m.p. 163-165 °C. Yield 87% (374 mg). Rf ~ 0.70. IR (neat, cm^{-1}): 3316 (N-H), 2952 (t-Bu), 2904 (t-Bu), 2868 (t-Bu), 1580 (C=O), 1523 (C=N), 1256 (C-

N), 1238 (C-N), 1174 (C-O), 1143 (C-O). ^{1}H NMR (600 MHz, $CDCl_3$) δ 7.69 (s, 1H), 7.64 (d, *J* = 2.3 Hz, 1H), 7.49 (d, *J* = 2.3 Hz, 1H), 7.28 (t, *J* = 8.1 Hz, 1H), 7.03 (s, 1H), 6.94 (dd, *J* = 8.1, 2.2 Hz, 1H), 6.85 (t, *J* = 2.2 Hz, 1H), 6.68 (dd, *J* = 8.1, 2.2 Hz, 1H), 6.51 (s, 1H), 3.81 (s, 3H), 1.51 (s, 9H), 1.36 (s, 9H). ^{13}C NMR (151 MHz, $CDCl_3$) δ 180.03, 160.69, 149.29, 147.83, 147.48, 141.80, 140.10, 139.41, 136.71, 134.18, 130.27, 125.34, 123.78, 113.63, 109.73, 107.41, 103.14, 99.94, 55.36, 35.13, 34.83, 31.36, 30.03. HRMS (ESI) m/z: $[M+H]^+$ Calcd. for $C_{27}H_{31}N_2O_3$ 431.2329; Found 431.2339.

6,8-Di-*tert*-butyl-2-((3-chlorophenyl)amino)-3*H*-phenoxazin-3-one (**4d**)

Brown needles with m.p. 171-174 °C. Yield 79% (344 mg). Rf ~ 0.70. IR (neat, cm^{-1}): 3274 (N-H), 2952 (t-Bu), 2905 (t-Bu), 2866 (t-Bu), 1574 (C=O), 1519 (C=N), 1264 (C-N), 1239 (C-N), 1207 (C-O), 1157 (C-O). ^{1}H NMR (600 MHz, $CDCl_3$) δ 7.72 (s, 1H), 7.65 (d, *J* = 2.3 Hz, 1H), 7.51 (d, *J* = 2.3 Hz, 1H), 7.34 (t, *J* = 2.0 Hz, 1H), 7.29 (t, *J* = 8.0 Hz, 1H), 7.20 (dd, *J* = 8.0, 2.0 Hz, 1H), 7.09 (dd, *J* = 8.0, 2.0 Hz, 1H), 7.01 (s, 1H), 6.52 (s, 1H), 1.51 (s, 9H), 1.37 (s, 9H). ^{13}C NMR (151 MHz, $CDCl_3$) δ 179.69, 149.26, 147.97, 147.29, 141.27, 140.25, 139.46, 136.76, 135.25, 134.09, 130.49, 125.71, 124.21, 123.86, 120.86, 119.19, 103.15, 100.35, 35.12, 34.83, 31.32, 30.00. HRMS (ESI) m/z: $[M+H]^+$ Calcd. for $C_{26}H_{28}ClN_2O_2$ 435.1834; Found 435.1837.

6,8-Di-*tert*-butyl-2-((4-nitrophenyl)amino)-3*H*-phenoxazin-3-one (**4e**)

Dark orange powder with m.p. 180-183 °C. Yield 88% (391 mg). Rf ~ 0.70. IR (neat, cm^{-1}): 3250 (N-H), 2953 (t-Bu), 2904 (t-Bu), 2866 (t-Bu), 1637 (C=O), 1582 (C=N), 1574 (N=O), 1269 (C-N), 1240 (C-N), 1155 (C-O), 1111 (C-O). ^{1}H NMR (600 MHz, $CDCl_3$) δ 8.24 (d, *J* = 8.6 Hz, 2H), 8.15 (s, 1H), 7.67 (s, 1H), 7.56 (s, 1H), 7.41 (d, *J* = 8.6 Hz, 2H), 7.22 (s, 1H), 6.55 (s, 1H), 1.51 (s, 9H), 1.37 (s, 9H). ^{13}C NMR (151 MHz, $CDCl_3$) δ 179.39, 149.32, 148.37, 146.97, 145.27, 142.72, 139.75, 139.71, 136.98, 134.15, 126.73, 125.73, 124.24, 118.89, 103.50, 103.33, 35.22, 34.92, 31.35, 30.05. HRMS (ESI) m/z: $[M-H]^-$ calcd for $C_{26}H_{26}N_2O_2$ 444.1929; found 444.1933.

6,8-Di-*tert*-butyl-2-((2-nitrophenyl)amino)-3*H*-phenoxazin-3-one (**4f**)

Orange needles with m.p. 178-180 °C. Yield 93% (414 mg). Rf ~ 0.70. IR (neat, cm^{-1}): 3221 (N-H), 2997 (t-Bu), 2961 (t-Bu), 2869 (t-Bu), 1589 (C=O), 1572 (N=O), 1509 (C=N), 1262 (C-N), 1241 (C-N), 1153 (C-O), 1111 (C-O). ^{1}H NMR (600 MHz, $CDCl_3$) δ 10.19 (s, 1H), 8.23 (d, *J* = 8.4, 1H), 7.86 (d, *J* = 8.4 Hz, 1H), 7.64 (d, *J* = 2.3 Hz, 1H), 7.62 (t, *J* = 8.4 Hz, 1H), 7.54 (d, *J* = 2.3 Hz, 1H), 7.13 (t, *J* = 8.4 Hz, 1H), 6.56 (s, 1H), 1.51 (s, 9H), 1.36 (s, 9H). ^{13}C NMR (151 MHz, $CDCl_3$) δ 179.46, 149.08, 148.10, 147.26, 140.27, 139.73, 138.71, 136.94, 136.08, 135.04, 134.00, 126.91, 126.51, 124.15, 122.29, 120.56, 104.37, 103.76, 35.18, 34.86, 31.33, 30.01. HRMS (ESI) m/z: $[M-Na]^+$ calcd for $C_{26}H_{27}N_3NaO_4$ 468.1894; found 468.1887.

2-((4-Aminophenyl)amino)-6,8-di-*tert*-butyl-3*H*-phenoxazin-3-one (**4g**)

Dark green powder with m.p. 156-158 °C. Yield 68% (282 mg). Rf ~ 0.70. IR (neat, cm^{-1}): 3410 (N-H), 3262 (N-H), 3208 (N-H), 2953 (t-Bu), 2902 (t-Bu), 2867 (t-Bu), 1637 (C=O), 1573 (C=N), 1265 (C-N), 1240 (C-N), 1182 (C-O), 1155 (C-O). ^{1}H NMR (600 MHz, $CDCl_3$) δ 7.60 (d, *J* = 2.3 Hz, 1H), 7.49 (s, 1H), 7.45 (d, *J* = 2.3 Hz, 1H), 7.11 (d, *J* = 8.6 Hz, 2H), 6.74 (s, 1H), 6.69 (d, *J* = 8.6 Hz, 2H), 6.48 (s, 1H), 3.69 (s, 2H), 1.50 (s, 9H), 1.35 (s, 9H). ^{13}C NMR (151 MHz, $CDCl_3$) δ 180.22, 149.42, 147.63, 147.44, 143.86, 143.14, 139.24, 136.60, 134.18, 129.63, 124.71, 123.87, 123.50, 115.82, 103.04, 97.89, 35.08, 34.78, 31.34, 30.01. HRMS (ESI) m/z: $[M+H]^+$ Calcd. for $C_{26}H_{30}N_3O_2$ 416.2342; Found 416.2333.

Methyl 4-((6,8-di-*tert*-butyl-3-oxo-3H-phenoxazin-2-yl)amino)benzoate (**4h**)

Red brown powder with m.p. 174-177 °C. Yield 81% (371 mg). Rf ~ 0.70. IR (neat, cm^{-1}): 3264 (N-H), 2950 (t-Bu), 2902 (t-Bu), 2869 (t-Bu), 1713 (C=O), 1580 (C=O), 1531 (C=N), 1280 (C-N), 1265 (C-N), 1182 (C-O), 1156 (C-O), 1111 (C-O). ^{1}H NMR (600 MHz, $CDCl_3$) δ 8.04 (d, *J* = 8.7 Hz, 2H), 7.95 (s, 1H), 7.66 (d, *J* = 2.3 Hz, 1H), 7.52 (d, *J* = 2.3 Hz, 1H), 7.36 (d, *J* = 8.7 Hz, 2H), 7.16 (s, 1H), 6.53 (s, 1H), 3.90 (s, 3H), 1.51 (s, 9H), 1.37 (s, 9H). ^{13}C NMR (151 MHz, $CDCl_3$) δ 179.68, 166.39, 149.26, 148.06, 147.26, 143.35, 140.44, 139.55, 136.82, 134.14, 131.31, 126.00, 125.01, 124.02, 119.22, 103.23, 101.81, 51.98, 35.15, 34.86, 31.34, 30.02. HRMS (ESI) m/z: $[M-H]^-$ Calcd. for $C_{28}H_{29}N_2O_4$ 457.2133; Found 457.2131.

General procedure for the synthesis of compounds **5a**–**c**

309 mg (1 mmol) of 6,8-di-*tert*-butyl-3*H*-phenoxazin-3-one (**1**) powder was mixed with 3 mmol of the corresponding *o*-phenylenediamine powder, placed in a round-bottom flask, and heated in a glycerin bath for 30 minutes at a temperature of 250 °C, stirring the melt with a spatula every 10 minutes. The reaction mixture was separated by column chromatography (Al_2O_3, eluent dichloromethane and isopropanol, *l* = 40 cm, *d* = 15 mm). The resulting fraction was recrystallized from isopropanol.

2,4-Di-*tert*-butyl-14*H*-quinoxalino[2,3-*b*]phenoxazine (**5a**)

Red powder with m.p. > 260 °C. Yield 93% (369 mg). Rf ~ 0.45. IR (neat, cm^{-1}): 3264 (N-H), 3087 (t-Bu), 2949 (t-Bu), 2902 (t-Bu), 2865 (t-Bu), 1618 (C=N), 1593 (C=N), 1235 (C-N), 1211 (C-N), 1179 (C-N), 1139 (C-O), 1113 (C-O). ^{1}H NMR (600 MHz, $CDCl_3$) δ 8.01 (s 1H), 7.96 (d, *J* = 7.8 Hz, 1H), 7.87 (d, *J* = 7.8 Hz, 1H), 7.48 (t, *J* = 7.8 Hz, 2H), 7.47 (t, *J* = 7.8 Hz, 2H) 7.26 (s, 1H), 6.85 (s, 1H), 6.74 (d, *J* = 2.4 Hz, 1H), 6.42 (d, *J* = 2.4 Hz, 1H), 1.46 (s, 9H), 1.13 (s, 9H). ^{13}C NMR (151 MHz, $CDCl_3$) δ 148.97, 146.46, 144.11, 143.58, 142.19, 141.67, 138.09, 136.94, 136.53, 129.10, 128.98, 128.02, 127.80, 127.28, 117.04, 109.69, 109.64, 102.35, 34.93, 34.38, 31.20, 29.98. ^{15}N NMR (60 MHz, $CDCl_3$) δ 314.85, 296.56, 92.79. HRMS (ESI) m/z: $[M+H]^+$ Calcd. for $C_{26}H_{28}N_3O$ 398.2227; Found 398.2218.

2,4-Di-*tert*-butyl-9,10-dimethyl-14*H*-quinoxalino[2,3-*b*]phenoxazine (**5b**)

Red powder with m.p. > 260 °C. Yield 69% (293 mg). Rf ~ 0.48. IR (neat, cm^{-1}): 3245 (N-H), 2952 (t-Bu), 2867 (t-Bu), 1615 (C=N), 1592 (C=N), 1238 (C-N), 1212 (C-N), 1168 (C-N), 1110 (C-O), 1032 (C-O). ^{1}H NMR (600 MHz, $CDCl_3$) δ 7.72 (s, 1H), 7.65 (s, 1H), 7.25 (s, 1H), 6.79 (s, 1H), 6.74 (d, *J* = 1.9 Hz, 1H), 6.42 (d, *J* = 1.9 Hz, 1H), 2.37 (s, 3H), 2.36 (s, 3H), 1.46 (s, 9H), 1.15 (s, 9H). ^{13}C NMR (151 MHz, $CDCl_3$) δ 148.36, 146.23, 143.42, 142.80, 141.43, 140.93, 140.04, 138.90, 138.12, 136.90, 135.83, 127.63, 127.61, 126.71, 116.87, 109.79, 109.48, 102.86, 34.91, 34.36, 31.20, 29.95, 20.35, 20.26. HRMS (ESI) m/z: $[M+H]^+$ Calcd. for $C_{28}H_{32}N_3O$ 426.2540; Found 426.2534.

Ethyl 2,4-di-*tert*-butyl-14*H*-quinoxalino[2,3-*b*]phenoxazine-10-carboxylate (**5c**)

Violet powder with m.p. > 260 °C. Yield 63% (295 mg). Rf ~ 0.54. IR (neat, cm^{-1}): 3330 (N-H), 2952 (t-Bu), 2902 (t-Bu), 2866 (t-Bu), 1701 (C=O), 1646 (C=N), 1592 (C=N), 1261 (C-N), 1233 (C-N), 1196 (C-N), 1174 (C-O), 1088 (C-O). ^{1}H NMR (600 MHz, $CDCl_3$) (600 MHz, $CDCl_3$) δ 8.64 (s, 1H), 8.11 (d, *J* = 8.8 Hz, 1H), 7.98 (d, *J* = 8.8 Hz, 1H), 7.65 – 7.45 (m, 2H), 6.84 (s, 1H), 6.80 (s, 1H), 6.46 (s, 1H), 4.36 (q, *J* = 7.1 Hz, 2H), 1.44 (s, 9H), 1.35 (t, *J* = 7.1 Hz, 3H), 1.18 (s, 9H). ^{13}C NMR (151 MHz, $CDCl_3$) δ 166.08, 150.01, 146.82, 145.07, 144.82, 143.39, 141.54, 138.03, 137.21, 136.64, 131.11, 130.57, 129.04, 127.32, 126.97, 117.49, 109.79, 109.55, 102.72, 61.35, 34.96,

34.49, 31.25, 29.96, 14.25. HRMS (ESI) m/z: $[M+H]^+$ Calcd. for $C_{29}H_{31}N_3O_3$ 470.2438; Found 470.2444.

General procedure for the synthesis of compounds **6a**,**b**

100 mg (0.25 mmol) of 2,4-di-*tert*-butyl-14*H*-quinoxalino[2,3-b]phenoxazine (**5a**) in 15 ml of acetone was added to 40 mg (1 mmol) of sodium hydroxide. After the solution took on an intense blue color, 0.1 ml (1.6 mmol) methyl iodide was added for **6a** or 254 mg (1 mmol) 1-iodonanane for **6b**. This was stirred at room temperature for 4 hours. The reaction mixture was filtered, and the mother liquor was recrystallized from acetone.

2,4-Di-*tert*-butyl-14-methyl-14*H*-quinoxalino[2,3-*b*]phenoxazine (**6a**)

Red powder with m.p. 232-235 °C. Yield 97% (398 mg). Rf ~ 0.80. IR (neat, cm^{-1}): 2950 (t-Bu), 2924 (t-Bu), 2860 (t-Bu), 1579 (C=N), 1563 (C=N), 1342 (C-N), 1301 (C-N), 1236 (C-N), 1222 (C-N), 1128 (C-O), 1070 (C-O). ^1H NMR (600 MHz, $CDCl_3$) δ 8.01 (d, *J* = 8.1 Hz, 1H), 7.98 (d, *J* = 8.1 Hz, 1H), 7.63 (t, *J* = 8.1 Hz, 1H), 7.60 (t, *J* = 8.1 Hz, 1H), 7.34 (s, 1H), 6.94 (s, 1H), 6.93 (d, *J* = 2.1, 1H), 6.71 (d, *J* = 2.1, 1H), 3.37 (s, 3H), 1.46 (s, 9H), 1.31 (s, 9H). ^{13}C NMR (151 MHz, $CDCl_3$) δ 150.45, 146.30, 144.48, 143.17, 142.70, 141.90, 140.04, 139.19, 136.92, 130.84, 129.07, 128.90, 128.49, 128.31, 117.35, 109.17, 108.55, 103.54, 34.97, 34.83, 32.45, 31.45, 30.03. ^{15}N NMR (60 MHz, $CDCl_3$) δ 311.64, 305.64, 83.69. HRMS (ESI) m/z: $[M+H]^+$ Calcd. for $C_{27}H_{30}N_3O$ 412.2383; Found 412.2389.

2,4-Di-*tert*-butyl-14-nonyl-14*H*-quinoxalino[2,3-*b*]phenoxazine (**6b**)

Red powder with m.p. 154-157 °C. Yield 91% (476 mg). Rf ~ 0.73. IR (neat, cm^{-1}): 2948 (t-Bu), 2927 (t-Bu), 2863 (t-Bu), 1582 (C=N), 1568 (C=N), 1346 (C-N), 1299 (C-N), 1231 (C-N), 1220 (C-N), 1128 (C-O), 1083 (C-O). ^1H NMR (600 MHz, $CDCl_3$) δ 7.98 (d, *J* = 8.1 Hz, 1H), 7.97 (d, *J* = 8.1 Hz, 1H), 7.63 (t, *J* = 8.1 Hz, 1H), 7.59 (t, *J* = 8.1 Hz, 1H), 7.29 (s, 1H), 6.91 (s, 1H), 6.90 (d, *J* = 2.1, 1H), 6.70 (d, *J* = 2.1, 1H), 3.79 (t, *J* = 8.3, 2H), 1.85 (qvin, *J* = 7.6, 2H), 1.53 (m, 2H), 1.50 (m, 2H), 1.45 (s, 9H), 1.42 (m 2H), 1.35 (m, 2H), 1.32 (m, 2H), 1.31 (s, 9H), 1.28 (m, 2H), 0.89 (t, *J* = 7.0, 3H). ^{13}C NMR (151 MHz, $CDCl_3$) δ 149.87, 146.04, 144.76, 143.29, 142.74, 141.90, 139.44, 137.50, 136.82, 129.19, 129.02, 128.89, 128.34, 128.17, 117.25, 108.99, 108.41, 102.76, 45.61, 34.99, 34.77, 31.83, 31.41, 29.99, 29.54, 29.39, 29.21, 26.97, 24.74, 22.65, 14.07. ^{15}N NMR (60 MHz, $CDCl_3$) δ 310.36, 303.85, 96.38. HRMS (ESI) m/z: $[M+H]^+$ Calcd. for $C_{35}H_{46}N_3O$ 524.3635; Found 524.3640.

General procedure for the synthesis of compounds **10a**,**b**

309 mg (1 mmol) of 6,8-di-*tert*-butyl-3*H*-phenoxazin-3-one (**1**) powder was mixed with 3 mmol of the corresponding *o*-aminophenol powder, placed in a round-bottom flask, and heated in a glycerin bath for 30 minutes at a temperature of 250 °C, stirring the melt with a spatula every 10 minutes. The structure of the obtained products **10a**,**b** corresponded to those previously described [20].

2,4-Di-*tert*-butylbenzo[5,6][1,4]oxazino[2,3-*b*]phenoxazine (**10a**)

The reaction mixture was separated by column chromatography on Al_2O_3, eluent toluene, Rf ~ 0.70. Red powder. Yield 55% (219 mg). M.p. 228–229 °C.

2,4-Di-*tert*-butyl-9-nitrobenzo[5,6][1,4]oxazino[2,3-*b*]phenoxazine (**10b**)

The reaction mixture was separated by column chromatography on Al_2O_3, eluent toluene, Rf ~ 0.75. Red powder. Yield 65% (288 mg). M.p. > 260 °C.

Synthesis of 2,4-di-*tert*-butylbenzo[5,6][1,4]oxazino[2,3-*b*]phenothiazine (**10c**)

309 mg (1 mmol) of 6,8-di-*tert*-butyl-3*H*-phenoxazin-3-one (**1**) powder was mixed with 375 mg (3 mmol) of 2-aminobenzenethiol powder, placed in a round-bottom flask, and heated in a glycerin bath for 30 minutes at a temperature of 220 °C, stirring the melt with a spatula every 10 minutes. The reaction mixture was separated by column chromatography (Al_2O_3, eluent dichloromethane, *l* = 40 cm, *d* = 15 mm). Violet powder with m.p. 211-215 °C. Yield 36% (149 mg). Rf ~ 0.80. IR (neat, cm^{-1}): 2943 (t-Bu), 2867 (t-Bu), 1569 (C=N), 1516 (C=N), 1333 (C-N), 1310 (C-N), 1267 (C-N), 1241 (C-N), 1141 (C-O), 1122 (C-O), 569 (C-S). ^{1}H NMR (600 MHz, $CDCl_3$) δ 7.43 (d, *J* = 7.8 Hz, 1H), 7.28 (d, *J* = 2.3 Hz, 1H), 7.24 (d, *J* = 2.3 Hz, 1H), 7.23 – 7.19 (m, 1H), 7.13 – 7.07 (m, 2H), 6.76 (s, 1H), 6.57 (s, 1H), 1.43 (s, 9H), 1.30 (s, 9H). ^{13}C NMR (151 MHz, $CDCl_3$) δ 151.00, 149.15, 148.33, 147.26, 141.26, 141.17, 136.09, 135.39, 132.07, 131.27, 127.73, 127.70, 124.78, 124.71, 123.75, 121.77, 118.80, 108.44, 34.90, 34.62, 31.30, 29.89. HRMS (ESI) m/z: $[M+H]^+$ Calcd. for $C_{26}H_{27}N_2OS$ 415.1839; Found 415.1845.

Table S1: Compounds and reaction conditions (yield, temperature, solvent, time).

compound	yield %, *T*, 30 min	yield %, 144 °C, *o*-xylene, 120 min	yield %, 144 °C, *o*-xylene, *p*-TSA, 120 min	yield %, 110 °C, toluene, 120 min	yield %, 110 °C, toluene, p-TSA, 120 min	yield %, 82 °C, isopropanol, 120 min	yield %, 82 °C, isopropanol, CF_3CO_2H, 120 min
**5a**	93, 250 °C	—	—	—	—	—	—
**5b**	69, 250 °C	—	—	—	—	—	—
**5c**	63, 250 °C	—	—	—	—	—	—
**10a**	55, 250 °C	—	11	—	7	3	5
**10b**	65, 250 °C	—	10	—	9	2	7
**10c**	36, 220 °C	—	8	—	6	—	—

2. XRD study. Molecular geometries. Crystallographic parameters.

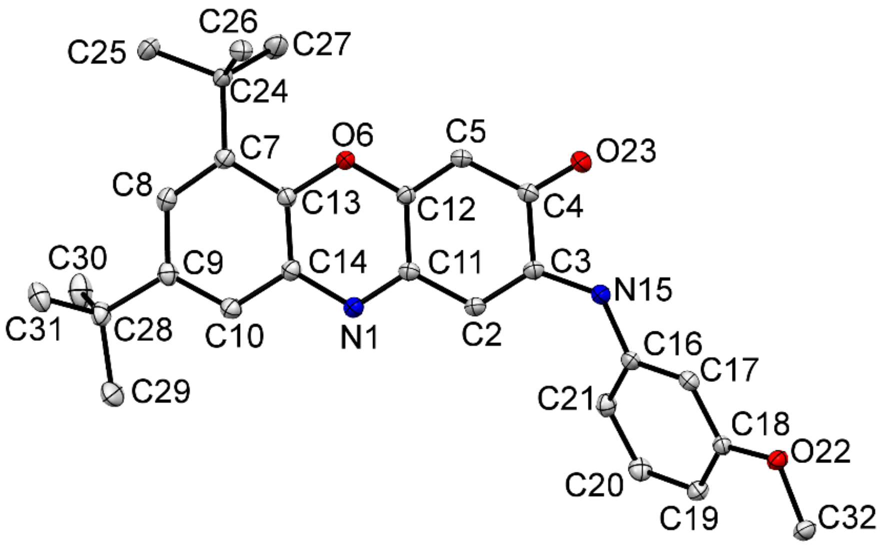

Figure S1: Molecular structure of 6,8-di-*tert*-butyl-2-((3-methoxyphenyl)amino)-3*H*-phenoxazin-3-one (**4c**).

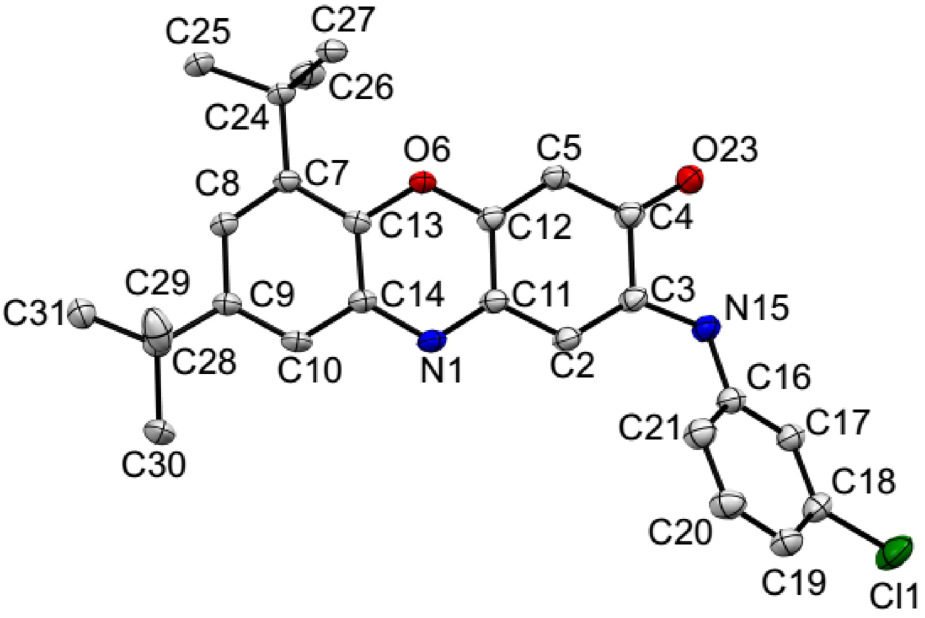

Figure S2: Molecular structure of 6,8-di-*tert*-butyl-2-((3-chlorophenyl)amino)-3*H*-phenoxazin-3-one (**4d**).

Table S2: Crystal data and structure refinement for **4c**,**d**,**f**.

parameter	**4c**	**4d**	**4f**
CCDC Number	2292841	2292840	2292847
Empirical formula	$C_{27}H_{30}N_2O_3$	$C_{26}H_{27}ClN_2O_2$	$C_{26}H_{27}N_3O_4$
Formula weight	430.53	434.94	445.50
Temperature/K	100.01(10)	100.01(13)	100.00(10)
Crystal system	monoclinic	monoclinic	orthorhombic
Space group	$P2_1/c$	$P2_1/n$	$P2_12_12_1$
a/Å	16.93490(10)	18.2211(3)	7.06800(10)
b/Å	15.04900(10)	18.0052(3)	16.9207(2)
c/Å	19.7911(2)	21.6236(4)	18.5820(2)
α/°	90	90	90
β/°	114.6770(10)	105.415(2)	90
γ/°	90	90	90
Volume/Å^3	4583.20(7)	6838.9(2)	2222.32(5)
Z	8	12	4
ρ_{calc}g/cm^3	1.248	1.267	1.332
μ/mm^{-1}	0.647	1.676	0.736
F(000)	1840.0	2760.0	944.0
Crystal size/mm^3	0.262 × 0.179 × 0.092	0.298 × 0.096 × 0.069	0.344 × 0.124 × 0.112
Radiation	CuKα (λ = 1.54184)	CuKα (λ = 1.54184)	CuKα (λ = 1.54184)
2Θ range for data collection/°	7.66 to 152.642	7.03 to 152.84	7.066 to 152.536
Index ranges	-21 ≤ h ≤ 21, -15 ≤ k ≤ 18, -24 ≤ l ≤ 24	-22 ≤ h ≤ 14, -22 ≤ k ≤ 22, -23 ≤ l ≤ 27	-8 ≤ h ≤ 8, -21 ≤ k ≤ 21, -23 ≤ l ≤ 23
Reflections collected	50573	60385	13501
Independent reflections	9568 [R_{int} = 0.0290, R_{sigma} = 0.0198]	14134 [R_{int} = 0.0376, R_{sigma} = 0.0291]	4476 [R_{int} = 0.0183, R_{sigma} = 0.0203]
Data/restraints/parameters	9568/0/599	14134/99/887	4476/0/353
Goodness-of-fit on F^2	1.036	1.069	1.032
Final R indexes [I>=2σ (I)]	R_1 = 0.0358, wR_2 = 0.0908	R_1 = 0.0676, wR_2 = 0.1835	R_1 = 0.0286, wR_2 = 0.0774
Final R indexes [all data]	R_1 = 0.0401, wR_2 = 0.0949	R_1 = 0.0805, wR_2 = 0.1947	R_1 = 0.0298, wR_2 = 0.0786
Largest diff. peak/hole / e Å^{-3}	0.23/-0.27	1.09/-0.70	0.26/-0.19
	-	-	0.65(6)

Table S3: X-ray-determined bond lengths and valence angles of **4c**. Bond lengths, Å.

O6-C12	1.3526(12)	C21-C20	1.3874(15)
O6-C13	1.3779(12)	C9-C28	1.5374(14)
O22-C18	1.3676(12)	C24-C26	1.5393(15)
O22-C32	1.4291(12)	C24-C25	1.5356(14)
O23-C4	1.2370(13)	C24-C27	1.5420(14)
N15-C3	1.3584(13)	C20-C19	1.3946(14)
N15-C16	1.4027(13)	C28-C30	1.5332(15)
N1-C11	1.3117(13)	C28-C31	1.5361(15)
N1-C14	1.3852(13)	C28-C29	1.5312(16)
C18-C17	1.3895(14)	C10-C14	1.4032(14)
C18-C19	1.3935(14)	C10-C9	1.3803(14)
C3-C2	1.3651(14)	C5-C4	1.4361(14)
C3-C4	1.5048(13)	C14-C13	1.3982(14)
C2-C11	1.4295(14)	C7-C8	1.3953(14)
C11-C12	1.4619(13)	C7-C13	1.4058(14)
C16-C17	1.3929(14)	C7-C24	1.5414(13)
C16-C21	1.3996(14)	C8-C9	1.4133(14)
C12-C5	1.3494(14)		

Valence angles of **4c**, deg.

C12-O6-C13	119.82(8)	C10-C9-C8	118.01(9)
C18-O22-C32	116.75(8)	C10-C9-C28	121.86(9)
C3-N15-C16	130.41(9)	C8-C9-C28	120.11(9)
C11-N1-C14	117.53(9)	C7-C24-C27	109.08(9)
O22-C18-C17	115.22(9)	C26-C24-C7	111.53(8)
O22-C18-C19	124.35(9)	C26-C24-C27	109.95(9)
C17-C18-C19	120.41(9)	C25-C24-C7	111.17(9)
N15-C3-C2	128.42(9)	C25-C24-C26	107.54(9)
N15-C3-C4	111.18(9)	C25-C24-C27	107.48(9)
C2-C3-C4	120.39(9)	C21-C20-C19	121.85(9)
C3-C2-C11	120.77(9)	C18-C19-C20	118.53(9)
N1-C11-C2	119.97(9)	C30-C28-C9	109.11(9)
N1-C11-C12	121.94(9)	C30-C28-C31	109.24(9)
C2-C11-C12	118.08(9)	C31-C28-C9	110.00(8)
C17-C16-N15	116.78(9)	C29-C28-C9	111.73(9)
C17-C16-C21	119.78(9)	C29-C28-C30	108.91(10)
C21-C16-N15	123.41(9)	C29-C28-C31	107.81(10)
O6-C12-C11	118.93(9)	C8-C7-C24	123.13(9)
C5-C12-O6	118.44(9)	C13-C7-C24	121.77(9)
C5-C12-C11	122.62(9)	C7-C8-C9	124.16(9)
C9-C10-C14	120.63(10)	O23-C4-C3	119.02(9)
C12-C5-C4	120.15(9)	O23-C4-C5	123.27(9)
N1-C14-C10	118.16(9)	C5-C4-C3	117.70(9)
N1-C14-C13	122.72(9)	O6-C13-C14	118.85(9)
C13-C14-C10	119.12(9)	O6-C13-C7	118.24(9)
C18-C17-C16	120.47(9)	C14-C13-C7	122.92(9)
C8-C7-C13	115.09(9)	C20-C21-C16	118.94(9)

Table S4: X-ray-determined bond lengths and valence angles of **4d**. Bond lengths, Å.

Cl1-C18	1.734(3)	C17-C16	1.389(3)
O6-C12	1.353(3)	C17-C18	1.386(4)
O6-C13	1.378(3)	C28-C31	1.535(4)
O23-C4	1.233(3)	C28-C29	1.519(4)
N1-C11	1.311(3)	C28-C30	1.536(4)
N1-C14	1.387(3)	C28-C34	1.501(19)
N15-C3	1.365(3)	C28-C33	1.559(18)
N15-C16	1.400(3)	C28-C32	1.546(19)
C12-C11	1.465(3)	C18-C19	1.379(4)
C12-C5	1.345(3)	C19-C20	1.385(4)
C13-C14	1.395(3)	C21-C20	1.387(4)
C13-C7	1.403(3)	C5-C4	1.442(3)
C11-C2	1.423(3)	C4-C3	1.496(3)
C9-C8	1.407(3)	C7-C24	1.537(3)
C9-C10	1.381(3)	C2-C3	1.362(3)
C9-C28	1.531(3)	C24-C27	1.540(3)
C14-C10	1.403(3)	C24-C25	1.526(3)
C7-C8	1.391(3)	C24-C26	1.542(3)

Valence angles of **4d**, deg.

C12-O6-C13	119.85(18)	N15-C16-C21	122.0(2)
C11-N1-C14	117.3(2)	C17-C16-N15	118.5(2)
C3-N15-C16	127.3(2)	C17-C16-C21	119.4(2)
O6-C12-C11	118.9(2)	C9-C28-C31	111.4(2)
C5-C12-O6	118.9(2)	C9-C28-C30	111.1(2)
C5-C12-C11	122.2(2)	C9-C28-C33	113.5(12)
O6-C13-C14	118.8(2)	C9-C28-C32	109.6(19)
O6-C13-C7	118.7(2)	C31-C28-C30	107.6(2)
C14-C13-C7	122.5(2)	C29-C28-C9	107.9(2)
N1-C11-C12	122.0(2)	C29-C28-C31	108.4(3)
N1-C11-C2	119.6(2)	C29-C28-C30	110.5(3)
C2-C11-C12	118.4(2)	C34-C28-C9	105.1(18)
C8-C9-C28	120.5(2)	C34-C28-C33	112(2)
C10-C9-C8	117.8(2)	C34-C28-C32	117(3)
C10-C9-C28	121.5(2)	C32-C28-C33	99(2)
N1-C14-C13	123.0(2)	C17-C18-Cl1	119.5(2)
N1-C14-C10	117.8(2)	C19-C18-Cl1	117.7(2)
C13-C14-C10	119.1(2)	C19-C18-C17	122.8(2)
C13-C7-C24	122.6(2)	C18-C19-C20	117.7(2)
C8-C7-C13	115.6(2)	C20-C21-C16	119.8(2)
C8-C7-C24	121.7(2)	C19-C20-C21	121.4(2)
C7-C8-C9	124.2(2)	C2-C3-N15	126.9(2)
C3-C2-C11	121.1(2)	C2-C3-C4	119.9(2)
C12-C5-C4	119.9(2)	C9-C10-C14	120.7(2)

O23-C4-C5	122.2(2)	C18-C17-C16	118.9(2)
O23-C4-C3	119.5(2)	C25-C24-C7	112.14(19)
C5-C4-C3	118.4(2)	C25-C24-C27	107.4(2)
C7-C24-C27	108.97(19)	C25-C24-C26	107.7(2)
C7-C24-C26	110.2(2)	N15-C3-C4	113.2(2)
C27-C24-C26	110.4(2)		

Table S5: X-ray-determined bond lengths and valence angles of **4f**. Bond lengths, Å.

O6-C12	1.3539(17)	C13-C7	1.405(2)
O6-C13	1.3813(17)	C13-C14	1.3997(19)
O23-C4	1.2314(19)	C11-C2	1.4374(19)
O31-N22	1.236(2)	C8-C7	1.392(2)
O32-N22	1.2247(19)	C7-C24	1.5444(18)
N15-C3	1.3765(19)	C3-C2	1.359(2)
N15-C16	1.383(2)	C24-C23	1.538(2)
N1-C11	1.3061(19)	C24-C26	1.534(2)
N1-C14	1.3836(18)	C24-C25	1.536(2)
N22-C17	1.458(2)	C16-C21	1.406(2)
C10-C9	1.377(2)	C16-C17	1.420(2)
C10-C14	1.398(2)	C21-C20	1.386(2)
C9-C8	1.413(2)	C17-C18	1.393(2)
C9-C27	1.5304(19)	C30-C27	1.534(2)
C12-C5	1.350(2)	C27-C28	1.537(2)
C12-C11	1.4578(19)	C27-C29	1.536(2)
C4-C5	1.444(2)	C18-C19	1.375(3)
C4-C3	1.508(2)	C20-C19	1.386(3)

Valence angles of **4f**, deg.

C12-O6-C13	119.84(11)	N1-C14-C13	122.55(13)
C3-N15-C16	131.12(14)	C10-C14-C13	119.70(13)
C11-N1-C14	117.55(12)	N15-C3-C4	110.51(13)
O31-N22-C17	119.13(13)	C2-C3-N15	130.07(14)
O32-N22-O31	122.25(15)	C2-C3-C4	119.41(13)
O32-N22-C17	118.61(15)	C23-C24-C7	111.32(12)
C9-C10-C14	120.63(13)	C26-C24-C7	109.04(13)
C10-C9-C8	117.66(13)	C26-C24-C23	107.78(13)
C10-C9-C27	122.71(13)	C26-C24-C25	109.95(13)
C8-C9-C27	119.63(13)	C25-C24-C7	111.14(13)
O6-C12-C11	118.59(13)	C25-C24-C23	107.55(13)
C5-C12-O6	119.59(13)	N15-C16-C21	122.45(14)
C5-C12-C11	121.81(13)	N15-C16-C17	121.93(15)
O23-C4-C5	122.33(14)	C21-C16-C17	115.62(14)
O23-C4-C3	119.34(13)	C3-C2-C11	121.33(14)
C5-C4-C3	118.32(13)	C20-C21-C16	121.71(16)

O6-C13-C7	118.88(12)	C16-C17-N22	122.19(14)
O6-C13-C14	118.84(13)	C18-C17-N22	115.78(14)
C14-C13-C7	122.28(13)	C18-C17-C16	122.02(16)
C12-C5-C4	120.37(14)	C9-C27-C30	109.12(13)
N1-C11-C12	122.56(13)	C9-C27-C28	111.62(12)
N1-C11-C2	118.74(13)	C9-C27-C29	109.58(13)
C2-C11-C12	118.70(13)	C30-C27-C28	108.36(14)
C7-C8-C9	124.54(13)	C30-C27-C29	109.79(13)
C13-C7-C24	122.65(13)	C29-C27-C28	108.35(14)
C8-C7-C13	115.17(13)	C19-C18-C17	120.32(16)
C8-C7-C24	122.17(13)	C21-C20-C19	121.10(17)
N1-C14-C10	117.75(13)	C18-C19-C20	119.06(16)

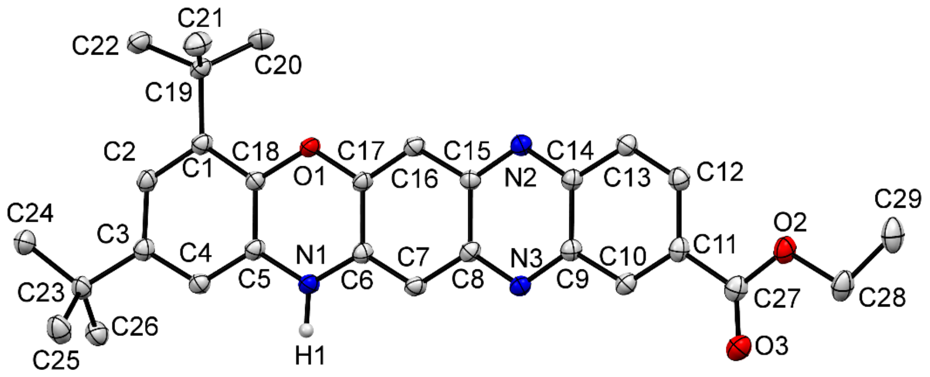

Figure S3: Molecular structure of ethyl 2,4-di-*tert*-butyl-14*H*-quinoxalino[2,3-*b*]phenoxazine-10-carboxylate (**5c**).

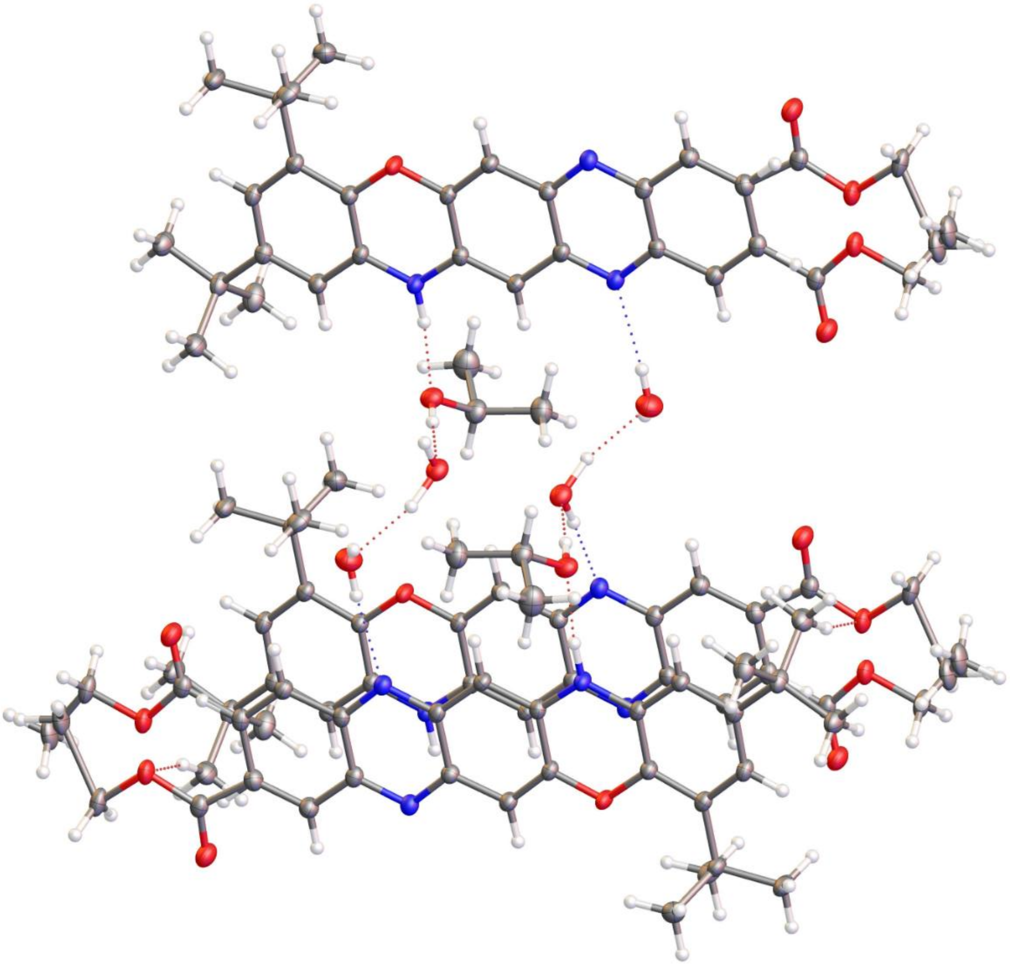

Figure S4: Molecular structure of the solvate of **5c**.

Table S6: Crystal data and structure refinement for **5c**.

parameter	**5c**
CCDC Number	2308520
Empirical formula	$C_{32}H_{43}N_3O_6$
Formula weight	565.69
Temperature/K	100.15
Crystal system	triclinic
Space group	P-1
a/Å	8.8807(3)
b/Å	9.8727(3)
c/Å	17.3935(8)
α/°	99.624(3)
β/°	91.935(3)
γ/°	97.449(3)
Volume/Å^3	1488.46(9)
Z	2
ρ_{calc}g/cm^3	1.262
μ/mm^{-1}	0.705
F(000)	608.0
Crystal size/mm^3	0.24 × 0.09 × 0.04
Radiation	CuKα (λ = 1.54184)

2Θ range for data collection/°	9.172 to 152.93
Index ranges	-11 ≤ h ≤ 11, -12 ≤ k ≤ 12, -21 ≤ l ≤ 21
Reflections collected	11566
Independent reflections	11566 [R_{int} = ?, R_{sigma} = 0.0372]
Data/restraints/parameters	11566/2/414
Goodness-of-fit on F^2	1.026
Final R indexes [I>=2σ (I)]	R_1 = 0.0529, wR_2 = 0.1599
Final R indexes [all data]	R_1 = 0.0693, wR_2 = 0.1735
Largest diff. peak/hole / e $Å^{-3}$	0.56/-0.25

Table S7: X-ray-determined bond lengths and valence angles of **5c**. Bond lengths, Å.

O4-C30	1.442(3)	C19-C22	1.540(3)
O1-C18	1.396(2)	C19-C21	1.533(3)
O1-C17	1.369(2)	C17-C16	1.358(3)
O2-C27	1.348(3)	C1-C2	1.407(3)
O2-C28	1.452(3)	C4-C3	1.393(3)
O3-C27	1.203(3)	C13-C14	1.419(3)
N2-C15	1.336(3)	C13-C12	1.369(3)
N2-C14	1.362(3)	C9-C14	1.426(3)
N3-C9	1.355(3)	C9-C10	1.421(3)
N3-C8	1.337(3)	C30-C32	1.511(3)
N1-C5	1.391(3)	C30-C31	1.516(3)
N1-C6	1.365(3)	C7-C8	1.417(3)
C5-C18	1.394(3)	C12-C11	1.428(3)
C5-C4	1.391(3)	C2-C3	1.392(3)
C18-C1	1.399(3)	C10-C11	1.371(3)
C15-C16	1.432(3)	C3-C23	1.534(3)
C15-C8	1.453(3)	C11-C27	1.490(3)
C6-C17	1.441(3)	C23-C26	1.540(3)
C6-C7	1.373(3)	C23-C25	1.532(3)
C19-C1	1.530(3)	C23-C24	1.527(3)
C19-C20	1.532(3)	C29-C28	1.513(4)

Valence angles of **5c**, deg.

C17-O1-C18	120.09(16)	N3-C9-C10	118.81(19)
C27-O2-C28	114.47(19)	C10-C9-C14	118.94(19)
C15-N2-C14	116.85(18)	N2-C14-C13	119.52(19)
C8-N3-C9	116.60(18)	N2-C14-C9	121.13(18)
C6-N1-C5	122.15(18)	C13-C14-C9	119.35(18)
N1-C5-C18	119.54(18)	O4-C30-C32	106.9(2)
C4-C5-N1	120.06(18)	O4-C30-C31	111.52(19)
C4-C5-C18	120.36(18)	C32-C30-C31	112.3(2)
O1-C18-C1	118.51(18)	C6-C7-C8	120.86(18)
C5-C18-O1	119.85(17)	C13-C12-C11	120.36(19)
C5-C18-C1	121.63(18)	C3-C2-C1	124.22(18)
N2-C15-C16	119.55(18)	C11-C10-C9	120.7(2)
N2-C15-C8	121.71(18)	C4-C3-C23	119.68(19)
C16 C15 C8	118.75(18)	C2-C3-C4	117.61(19)
N1-C6-C17	117.66(18)	C2-C3-C23	122.68(18)

N1-C6-C7	122.79(19)	C12-C11-C27	122.30(19)
C7-C6-C17	119.54(19)	C10-C11-C12	120.16(19)
C1-C19-C20	111.73(17)	C10-C11-C27	117.5(2)
C1-C19-C22	110.66(17)	C17-C16-C15	119.95(18)
C1-C19-C21	109.39(17)	N3-C8-C15	121.45(18)
C20-C19-C22	106.85(18)	N3-C8-C7	119.40(18)
C20-C19-C21	110.27(19)	C7-C8-C15	119.15(17)
C21-C19-C22	107.85(18)	C3-C23-C26	109.57(17)
O1-C17-C6	120.69(18)	C25-C23-C3	109.10(17)
C16-C17-O1	117.59(18)	C25-C23-C26	109.11(19)
C16-C17-C6	121.72(18)	C24-C23-C3	112.71(18)
C18-C1-C19	122.96(18)	C24-C23-C26	108.11(18)
C18-C1-C2	115.73(18)	C24-C23-C25	108.17(19)
C2-C1-C19	121.25(17)	O2-C27-C11	112.50(19)
C5-C4-C3	120.37(19)	O3-C27-O2	123.5(2)
C12-C13-C14	120.5(2)	O3-C27-C11	124.0(2)
N3-C9-C14	122.24(18)	O2-C28-C29	107.5(2)

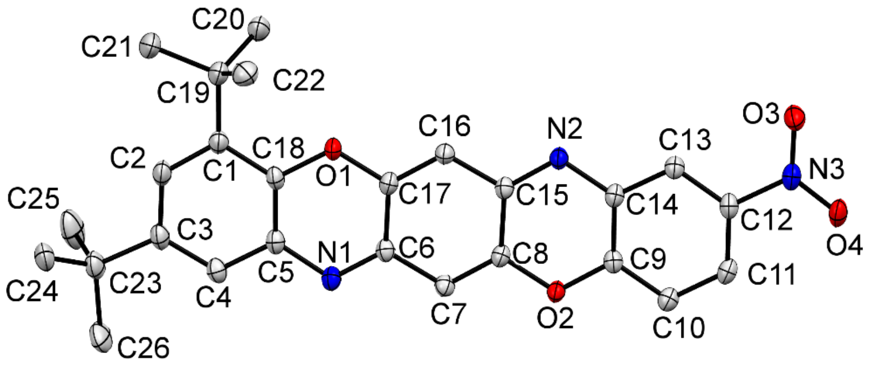

Figure S5: Molecular structure of 2,4-di-*tert*-butyl-9-nitrobenzo[5,6][1,4]oxazino[2,3-*b*]phenoxazine **10b**.

Table S8: Crystal data and structure refinement for **10b**.

parameter	**10b**
CCDC Number	2292848
Empirical formula	$C_{26}H_{25}N_3O_4$
Formula weight	443.49
Temperature/K	100.00(13)
Crystal system	monoclinic
Space group	$P2_1/c$
a/Å	18.1069(4)
b/Å	6.68620(10)
c/Å	18.7628(3)
α/°	90
β/°	96.324(2)
γ/°	90
Volume/Å^3	2257.72(7)
Z	4
ρ_{calc}g/cm^3	1.305
μ/mm^{-1}	0.724

F(000)	936.0
Crystal size/mm^3	0.339 × 0.182 × 0.07
Radiation	Cu Kα (λ = 1.54184)
2Θ range for data collection/°	9.486 to 152.62
Index ranges	-22 ≤ h ≤ 22, -8 ≤ k ≤ 8, -19 ≤ l ≤ 23
Reflections collected	23366
Independent reflections	4719 [R_{int} = 0.0332, R_{sigma} = 0.0229]
Data/restraints/parameters	4719/0/304
Goodness-of-fit on F^2	1.037
Final R indexes [I>=2σ (I)]	R_1 = 0.0464, wR_2 = 0.1296
Final R indexes [all data]	R_1 = 0.0545, wR_2 = 0.1375
Largest diff. peak/hole / e Å^{-3}	0.29/-0.28

Table S9: X-ray-determined bond lengths and valence angles of **10b**. Bond lengths, Å.

O1-C18	1.3832(15)	C18-C1	1.4014(18)
O1-C17	1.3648(16)	C9-C14	1.4033(19)
O2-C8	1.3681(15)	C17-C16	1.3526(18)
O2-C9	1.3695(15)	C14-C13	1.3977(17)
O3-N3	1.2293(16)	C13-C12	1.3851(18)
O4-N3	1.2306(16)	C5-C4	1.4022(18)
N2-C15	1.3119(17)	C12-C11	1.389(2)
N2-C14	1.3926(16)	C1-C2	1.3982(19)
N1-C6	1.3107(17)	C1-C19	1.534(2)
N1-C5	1.3909(17)	C2-C3	1.405(2)
N3-C12	1.4650(16)	C4-C3	1.381(2)
C8-C15	1.4595(18)	C3-C23	1.5367(18)
C8-C7	1.3491(18)	C19-C22	1.539(2)
C6-C17	1.4549(19)	C19-C20	1.538(2)
C6-C7	1.4349(18)	C19-C21	1.5370(19)
C15-C16	1.4331(18)	C23-C24	1.536(2)
C10-C9	1.3881(18)	C23-C26	1.526(2)
C10-C11	1.3848(18)	C23-C25	1.534(2)
C18-C5	1.400(2)		

Valence angles of **10b**, deg.

C17-O1-C18	119.12(11)	C8-C7-C6	119.98(12)
C8-O2-C9	118.37(10)	N1-C5-C18	122.58(12)
C15-N2-C14	116.19(11)	N1-C5-C4	118.23(12)
C6-N1-C5	116.96(12)	C18-C5-C4	119.19(12)
O3-N3-O4	123.65(11)	C13-C12-N3	118.70(12)
O3-N3-C12	118.56(11)	C13-C12-C11	123.03(12)
O4-N3-C12	117.79(12)	C11-C12-N3	118.26(11)
O2-C8-C15	118.82(11)	C17-C16-C15	120.18(12)
C7-C8-O2	118.02(12)	C10-C11-C12	118.57(12)
C7-C8-C15	123.16(12)	C18-C1-C19	122.29(12)
N1-C6-C17	123.07(12)	C2-C1-C18	115.45(13)
N1-C6-C7	119.98(12)	C2-C1-C19	122.26(12)
C7-C6-C17	116.95(11)	C1-C2-C3	124.09(13)
N2-C15-C8	123.57(12)	C3-C4-C5	120.69(13)

N2-C15-C16	119.75(12)	C2-C3-C23	119.49(13)
C16-C15-C8	116.67(11)	C4-C3-C2	118.01(12)
C11-C10-C9	118.99(12)	C4-C3-C23	122.49(14)
O1-C18-C5	119.42(11)	C1-C19-C22	110.17(12)
O1-C18-C1	118.05(12)	C1-C19-C20	110.08(12)
C5-C18-C1	122.52(12)	C1-C19-C21	111.42(12)
O2-C9-C10	116.70(12)	C20-C19-C22	110.34(12)
O2-C9-C14	120.59(11)	C21-C19-C22	107.30(13)
C10-C9-C14	122.70(12)	C21-C19-C20	107.46(12)
O1-C17-C6	118.76(11)	C24-C23-C3	110.25(13)
C16-C17-O1	118.28(12)	C26-C23-C3	111.54(12)
C16-C17-C6	122.96(12)	C26-C23-C24	107.80(13)
N2-C14-C9	122.40(11)	C26-C23-C25	109.64(15)
N2-C14-C13	119.77(12)	C25-C23-C3	108.43(12)
C13-C14-C9	117.82(12)	C25-C23-C24	109.16(14)
C12-C13-C14	118.86(12)		

3. DFT study

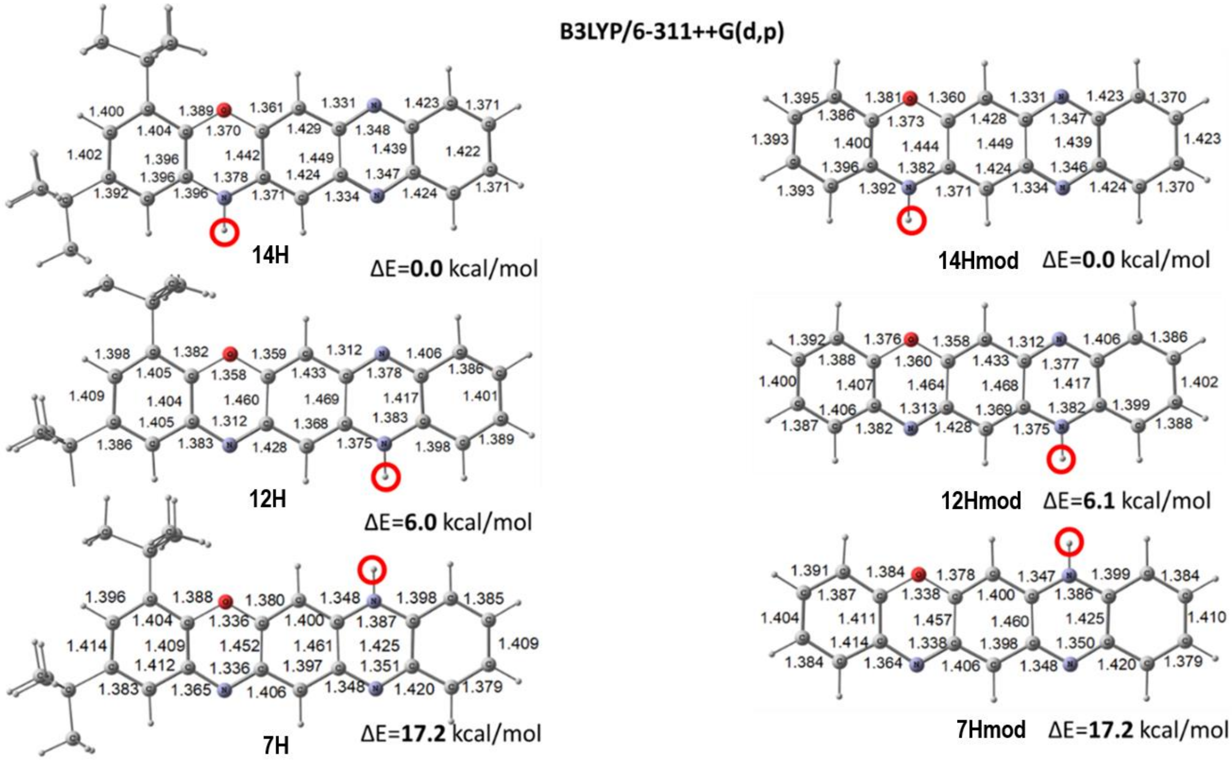

Figure S6: Geometry and relative stability of the tautomeric forms of quinoxaline[2,3-*b*]phenoxazine and corresponding 6,8-di-*tert*-butyl derivatives calculated using the DFT B3LYP/6-311++G(d,p) method.

4. UV–vis and luminescence spectra

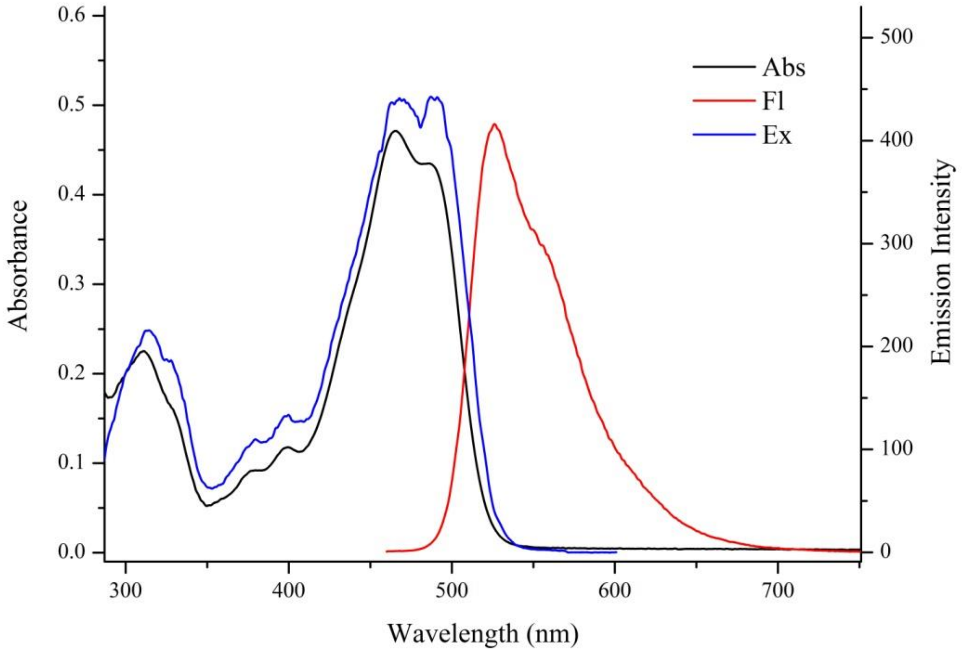

Figure S7: UV–vis, fluorescence emission (λ_{ex} = 365 nm), and fluorescence excitation (λ_{obs} = 610 nm) spectra of compound **5a** (toluene, *c* = 2·10^{-5} M, *l* = 1 cm, *T* = 293 K).

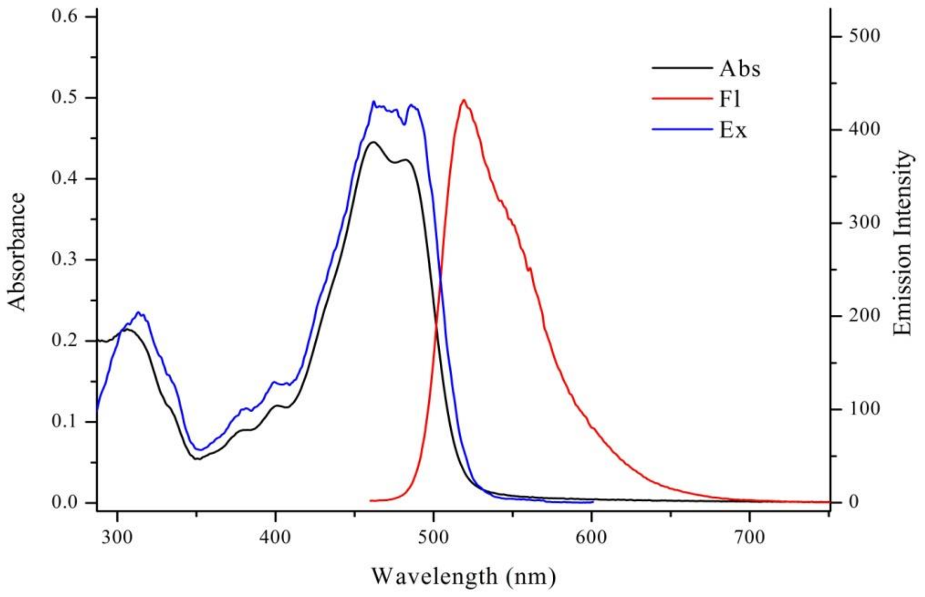

Figure S8: UV–vis, fluorescence emission (λ_{ex} = 365 nm) and fluorescence excitation (λ_{obs} = 610 nm) spectra of compound **5b** (toluene, *c* = 2·10^{-5} M, *l* = 1 cm, *T* = 293 K).

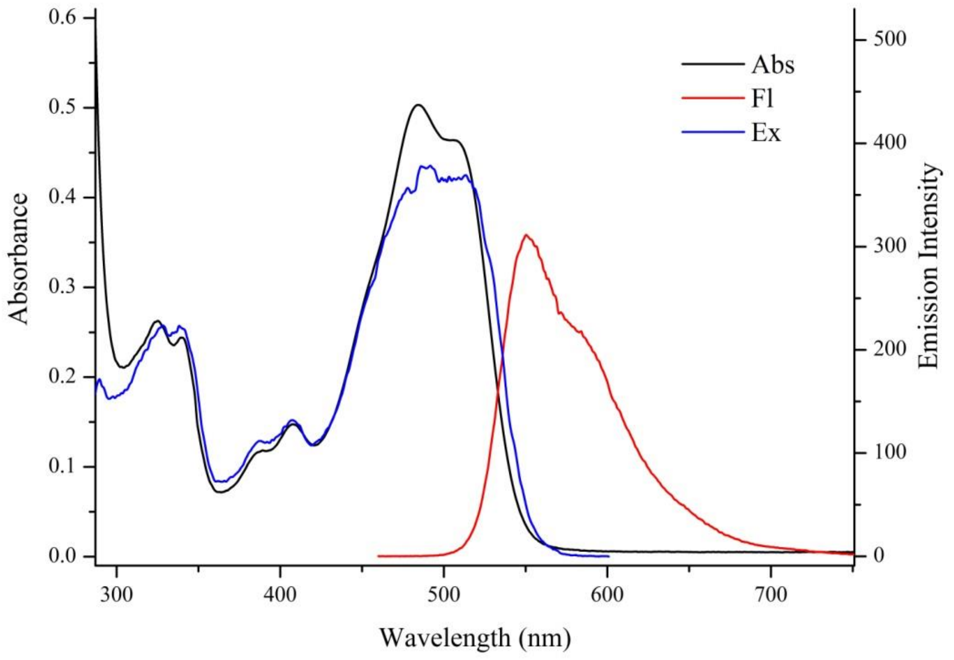

Figure S9: UV–vis, fluorescence emission (λ_{ex} = 365 nm) and fluorescence excitation (λ_{obs} = 630 nm) spectra of compound **5c** (toluene, $c = 2 \cdot 10^{-5}$ M, l = 1 cm, T = 293 K).

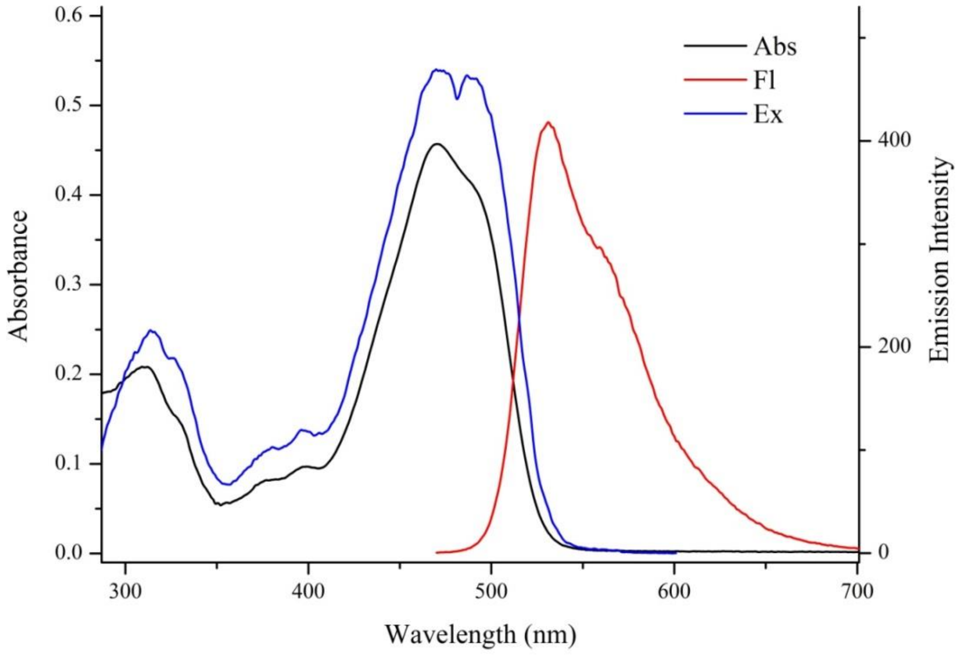

Figure S10: UV–vis, fluorescence emission (λ_{ex} = 365 nm) and fluorescence excitation (λ_{obs} = 610 nm) spectra of compound **6a** (toluene, $c = 2 \cdot 10^{-5}$ M, l = 1 cm, T = 293 K).

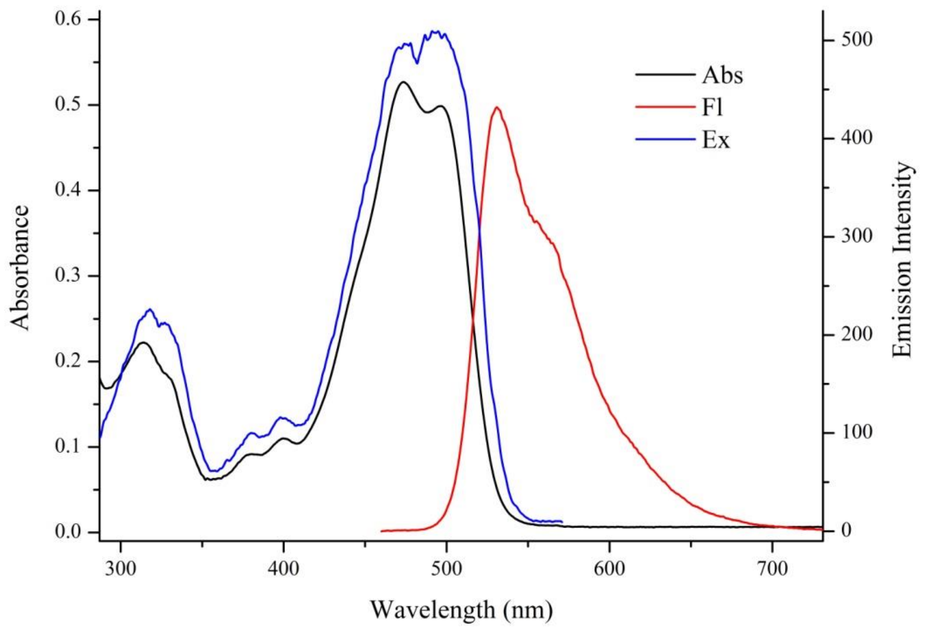

Figure S11: UV–vis, fluorescence emission (λ_{ex} = 365 nm) and fluorescence excitation (λ_{obs} = 610 nm) spectra of compound **6b** (toluene, $c = 2 \cdot 10^{-5}$ M, l = 1 cm, T = 293).

5. Cyclic voltammetry

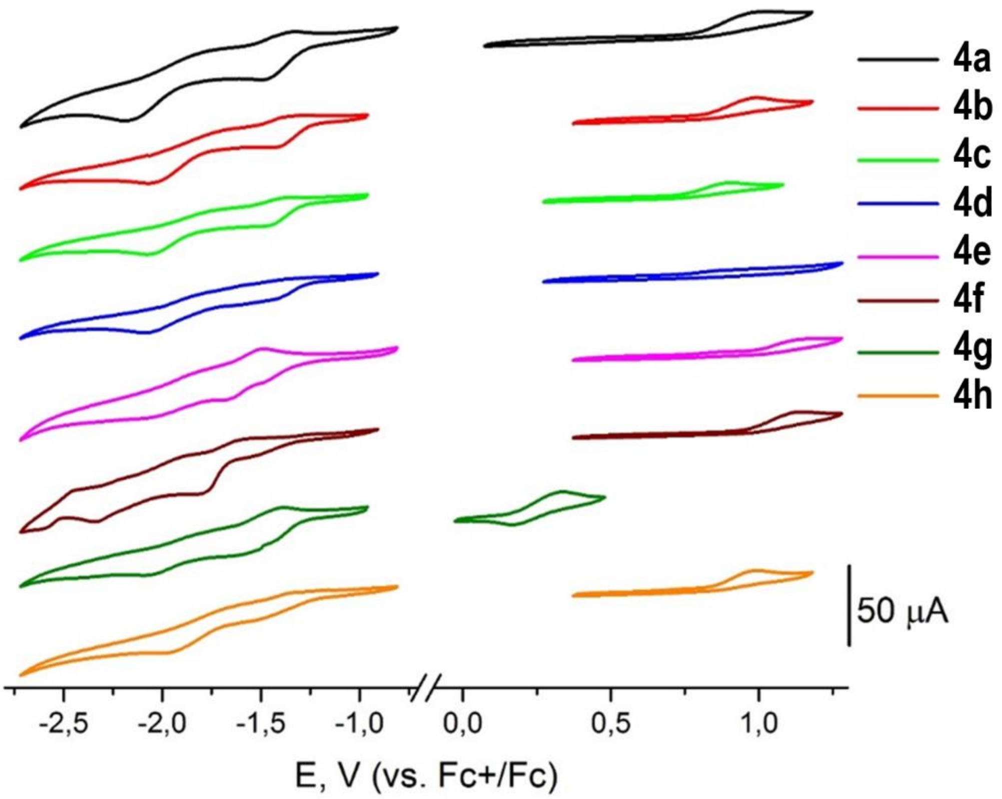

Figure S12: Cyclic voltammetry curves of **4a–h** (CH_2Cl_2, 50 mV/sec, *c* = 5 mM).

6. NMR spectra

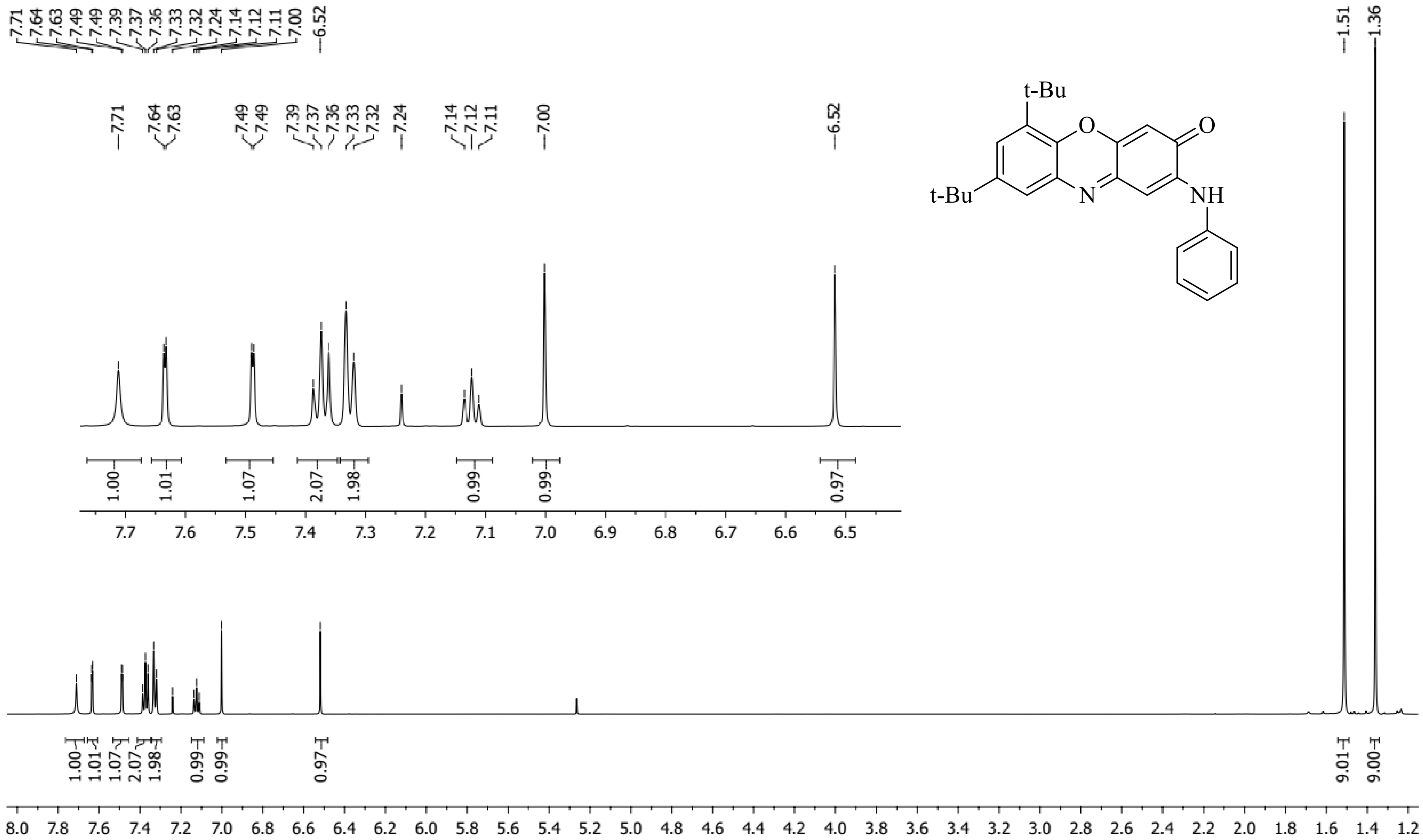

Figure S13: ^{1}H NMR spectrum of 6,8-di-*tert*-butyl-2-(phenylamino)-3*H*-phenoxazin-3-one (**4a**).

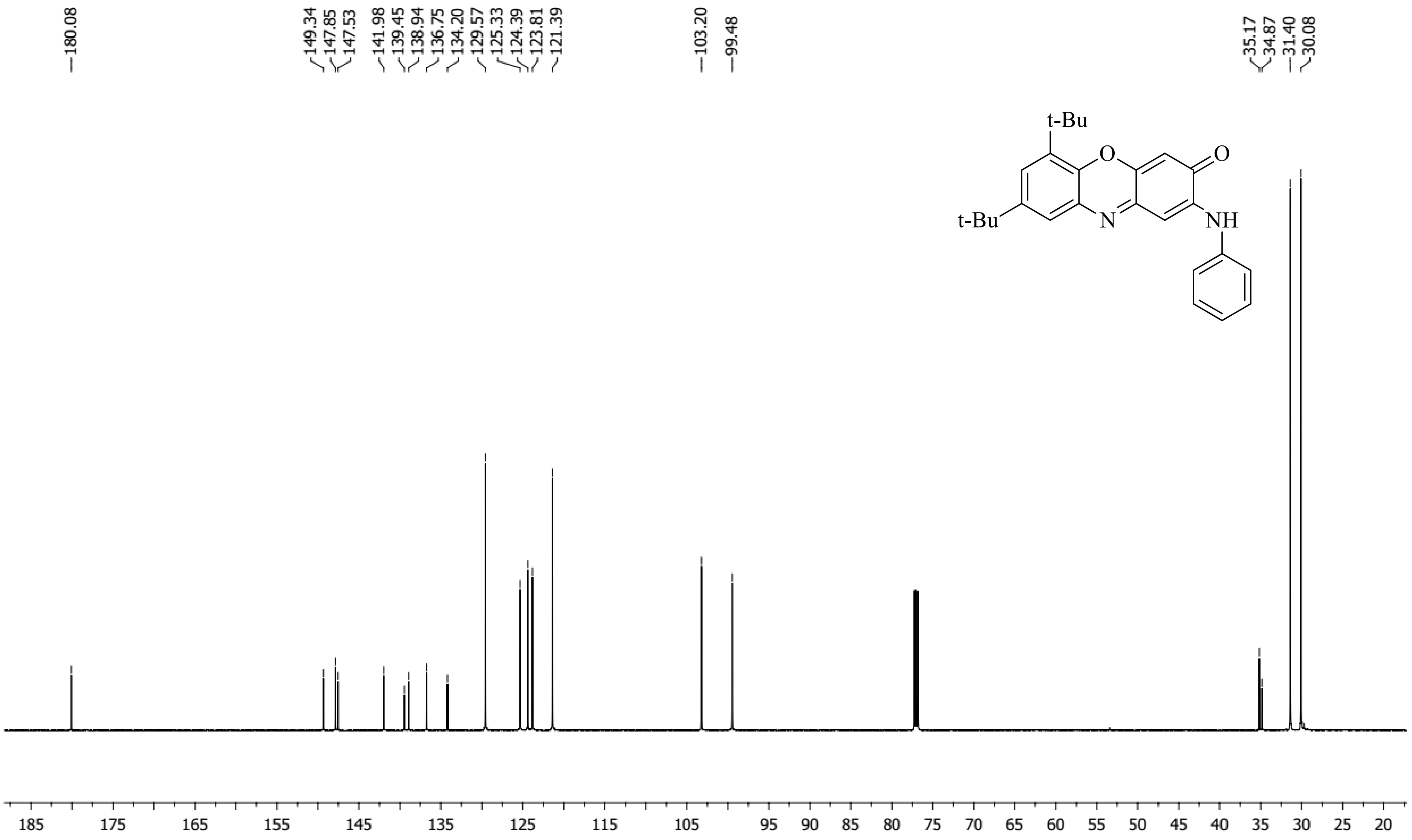

Figure S14: ^{13}C NMR spectrum of 6,8-di-*tert*-butyl-2-(phenylamino)-3*H*-phenoxazin-3-one (**4a**).

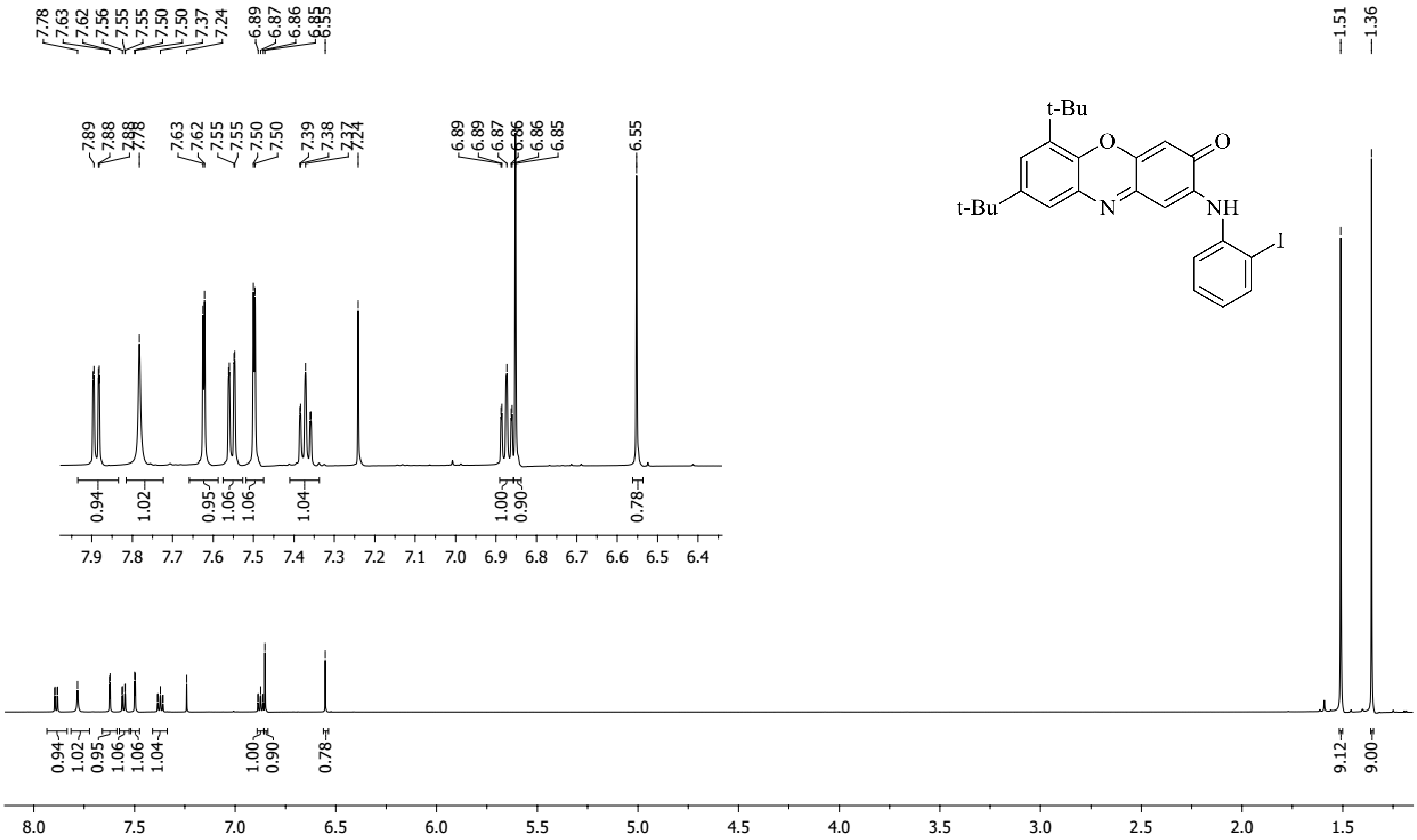

Figure S15: ^{1}H NMR spectrum of 6,8-di-*tert*-butyl-2-((2-iodophenyl)amino)-3*H*-phenoxazin-3-one (**4b**).

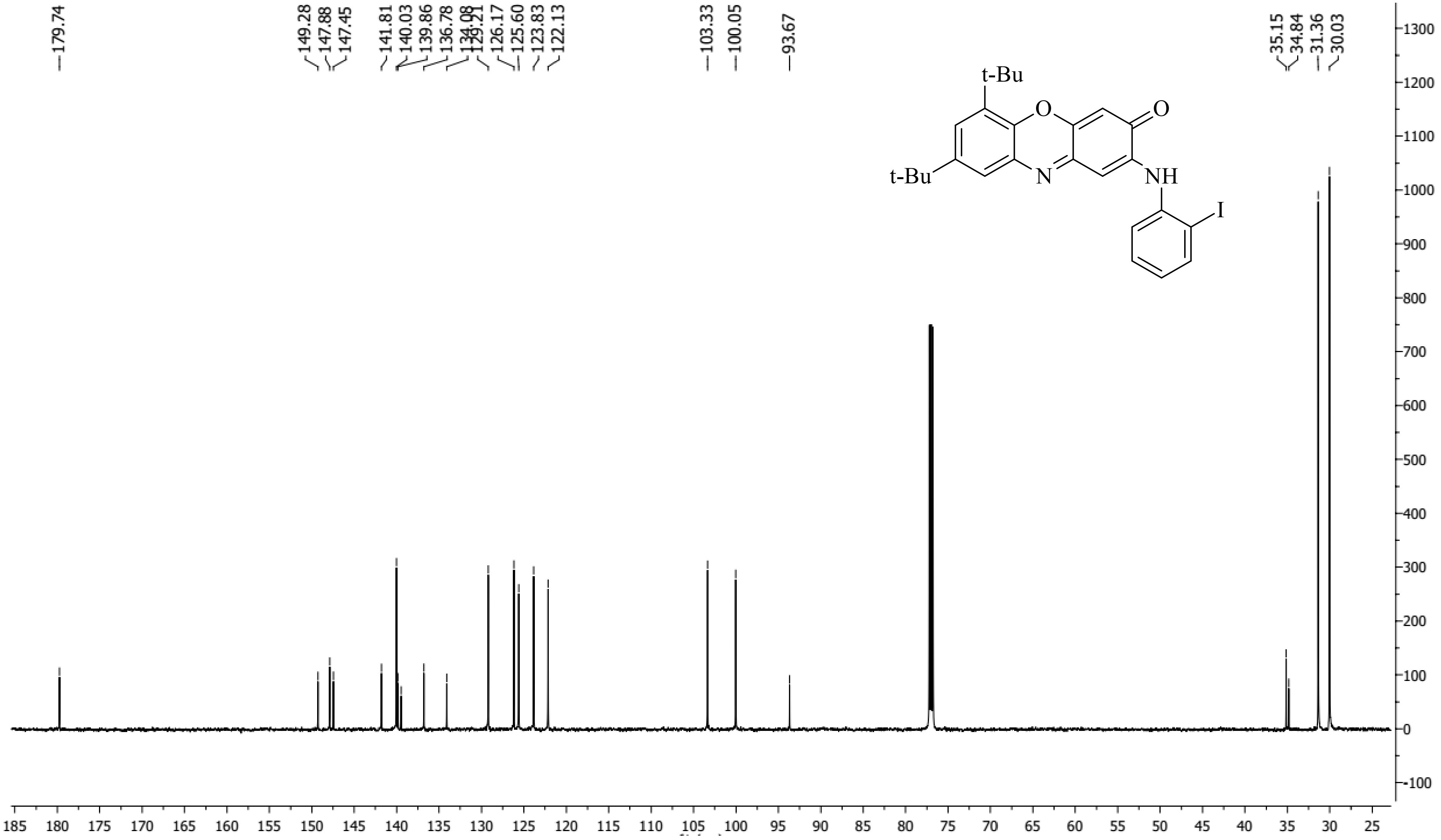

Figure S16: ^{13}C NMR spectrum of 6,8-di-*tert*-butyl-2-((2-iodophenyl)amino)-3*H*-phenoxazin-3-one (**4b**).

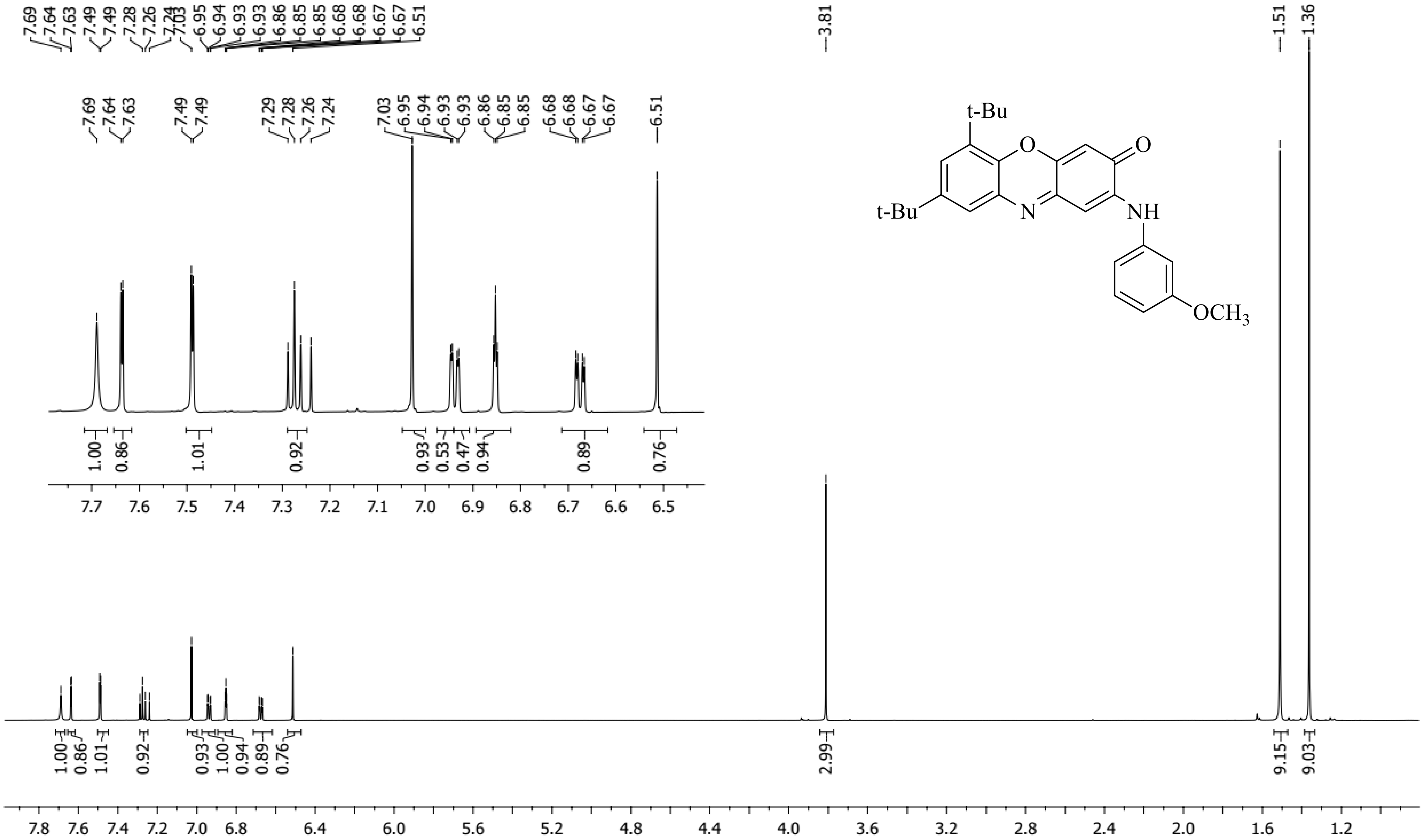

Figure S17: ^{1}H NMR spectrum of 6,8-di-*tert*-butyl-2-((3-methoxyphenyl)amino)-3*H*-phenoxazin-3-one (**4c**).

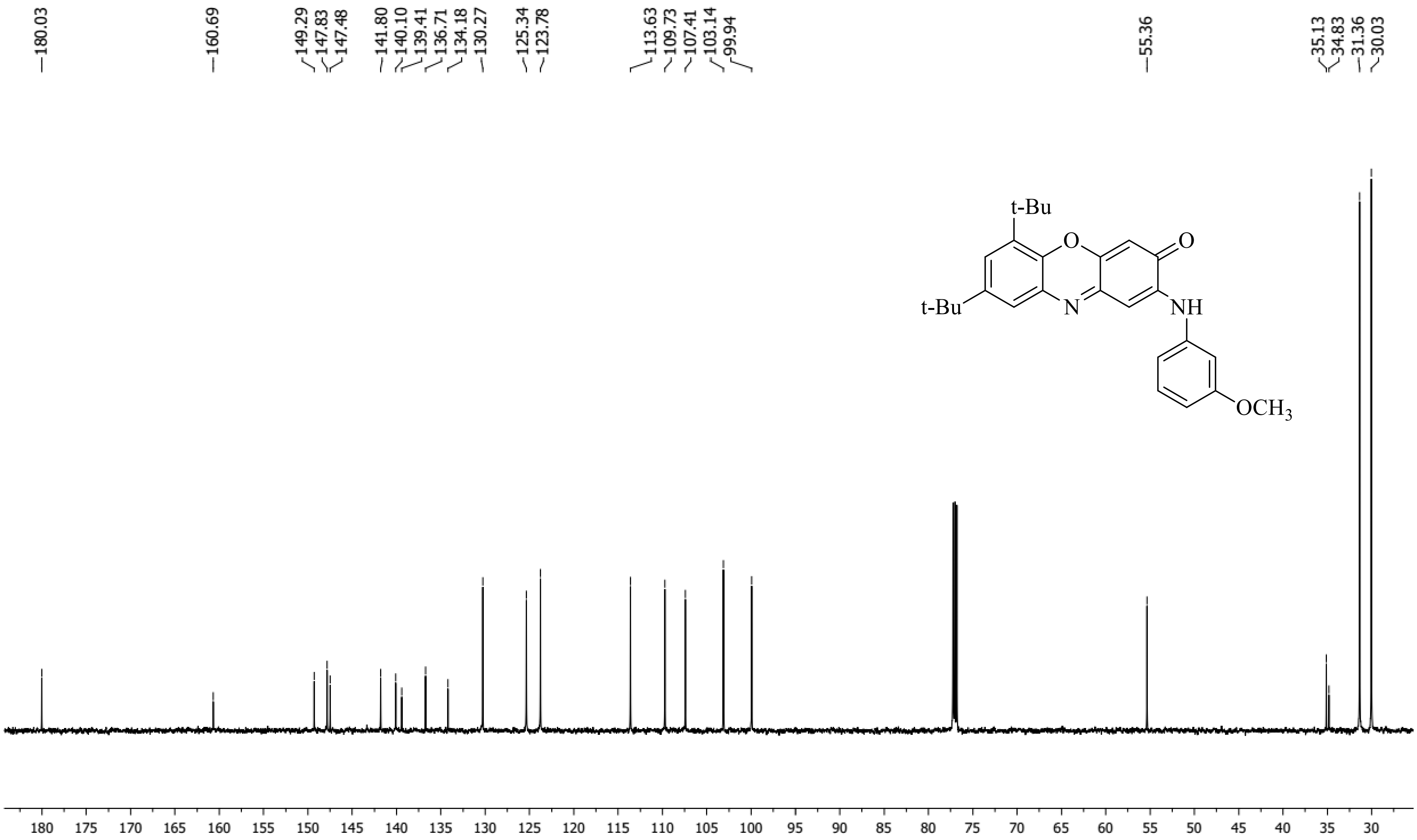

Figure S18: ^{13}C NMR spectrum of 6,8-di-*tert*-butyl-2-((3-methoxyphenyl)amino)-3*H*-phenoxazin-3-one (**4c**).

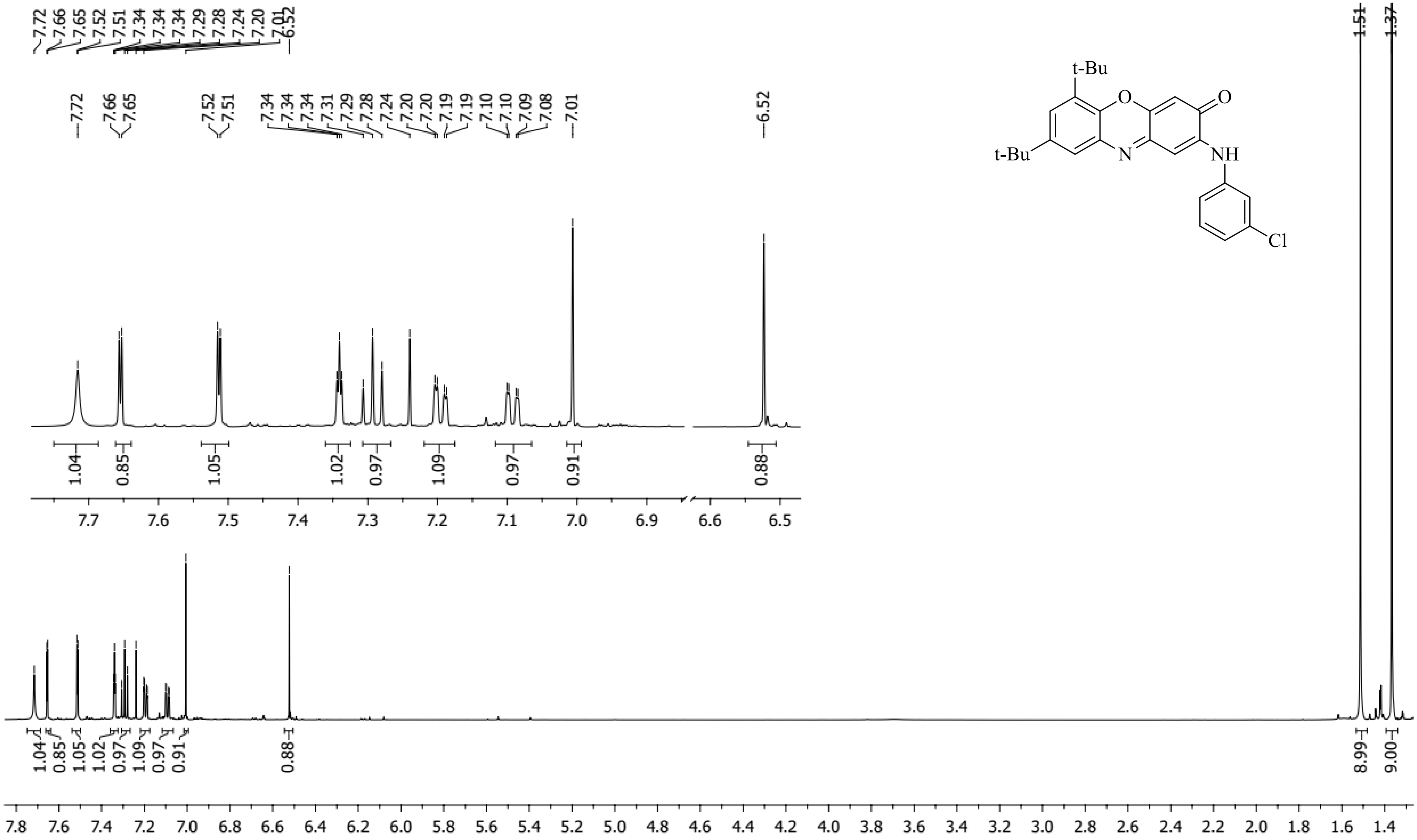

Figure S19: ^{1}H NMR spectrum of 6,8-di-*tert*-butyl-2-((3-chlorophenyl)amino)-3*H*-phenoxazin-3-one (**4d**).

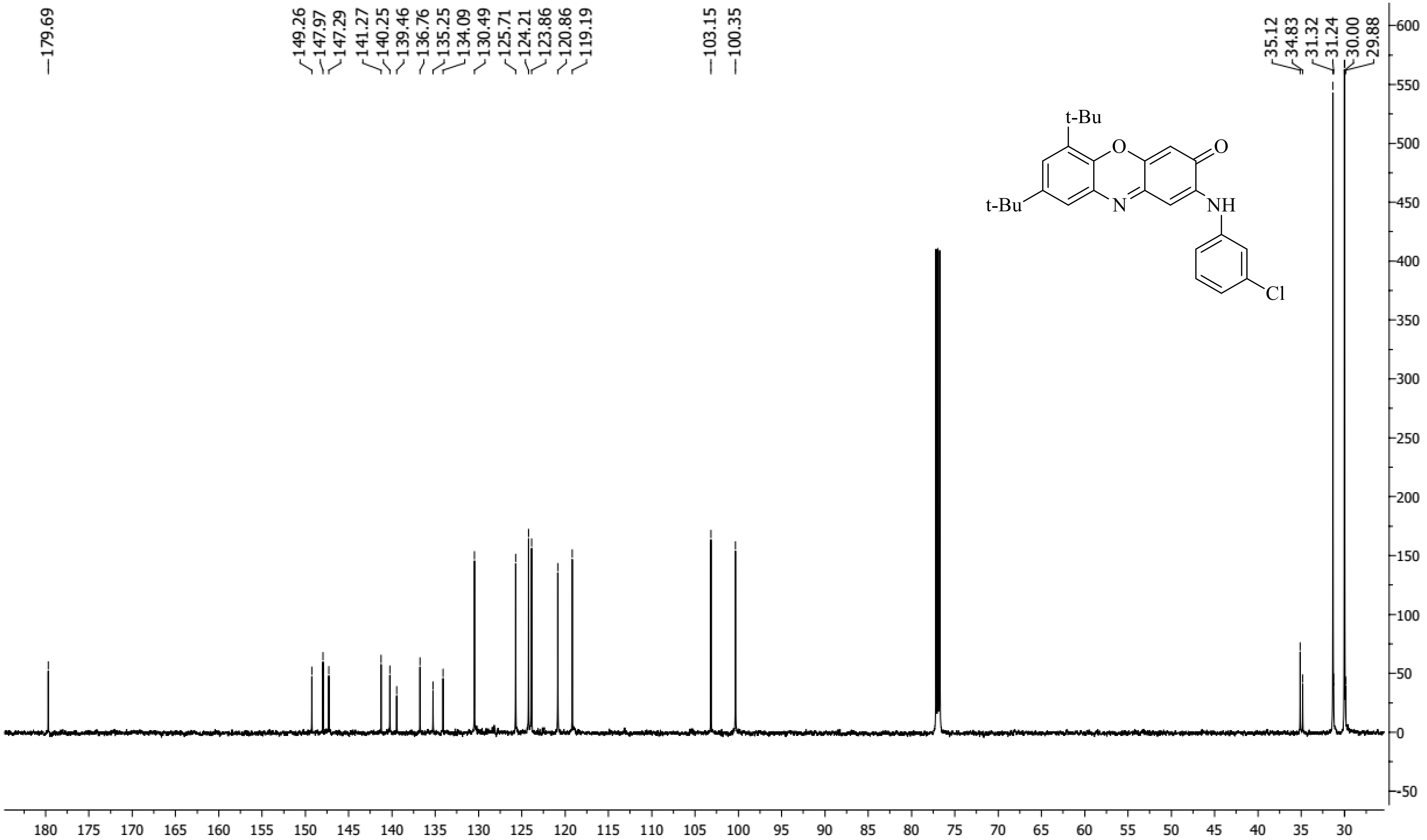

Figure S20: ^{13}C NMR spectrum of 6,8-di-*tert*-butyl-2-((3-chlorophenyl)amino)-3*H*-phenoxazin-3-one (**4d**).

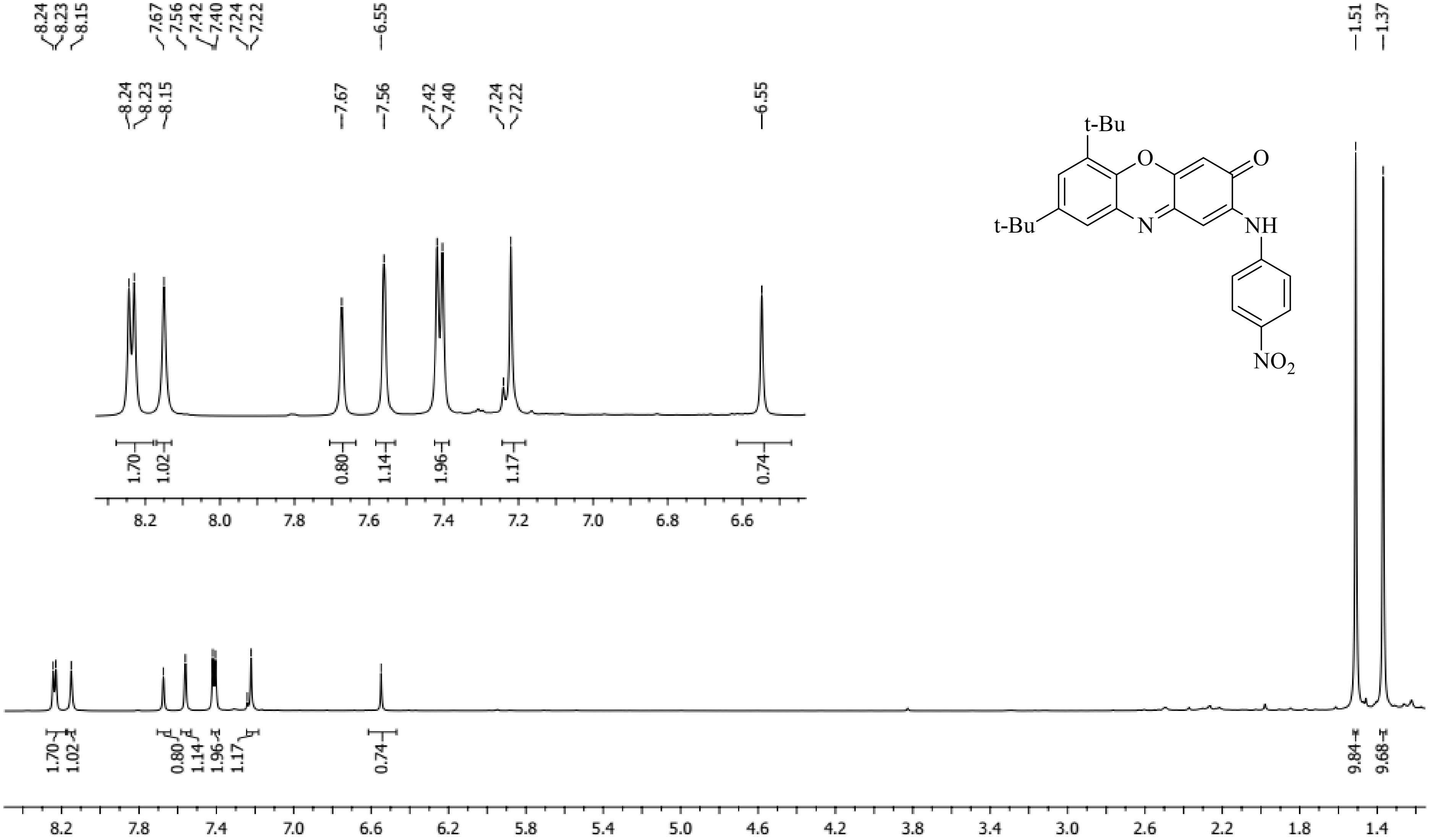

Figure S21: ^{1}H NMR spectrum of 6,8-di-*tert*-butyl-2-((4-nitrophenyl)amino)-3*H*-phenoxazin-3-one (**4e**).

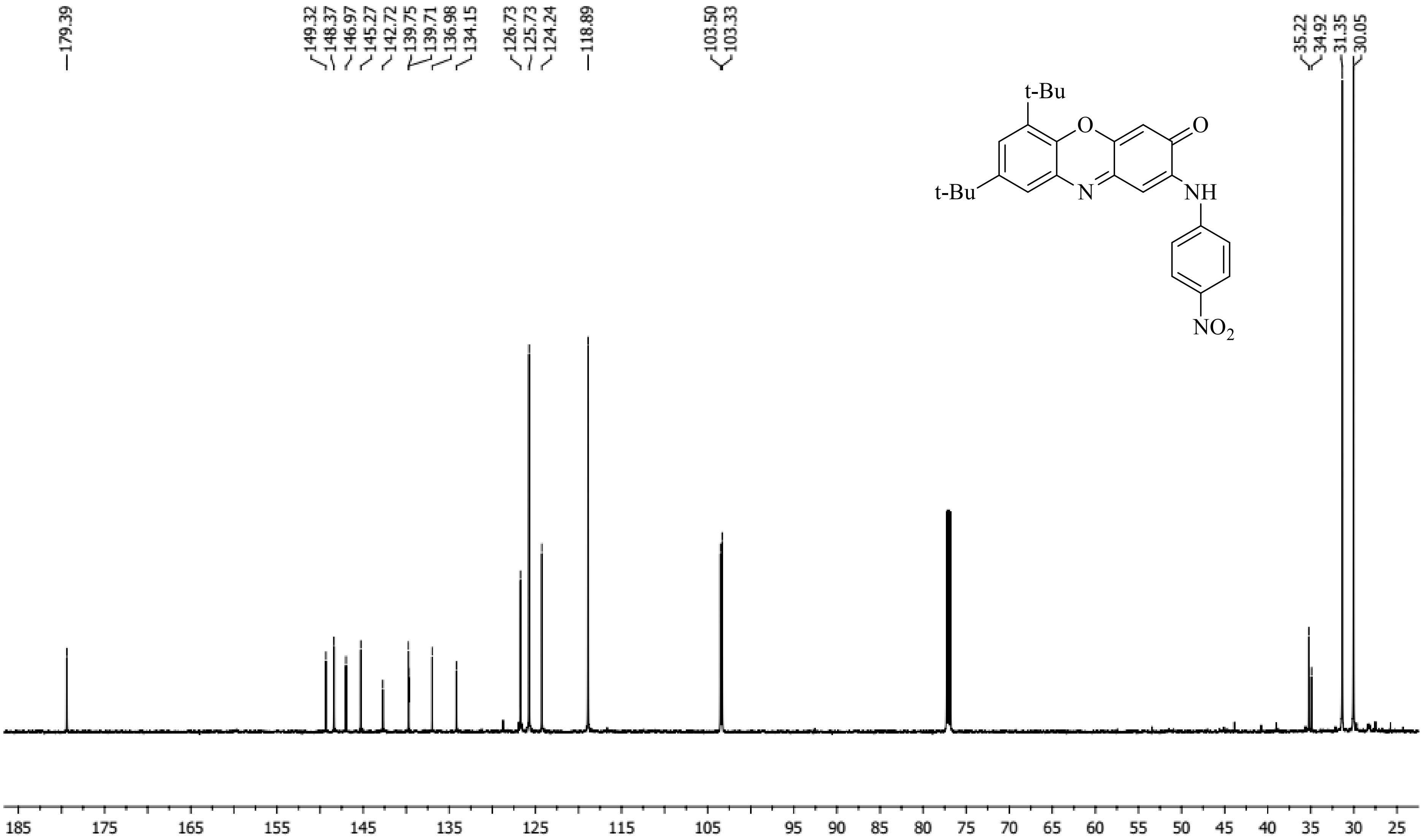

Figure S22: ^{13}C NMR spectrum of 6,8-di-*tert*-butyl-2-((4-nitrophenyl)amino)-3*H*-phenoxazin-3-one (**4e**).

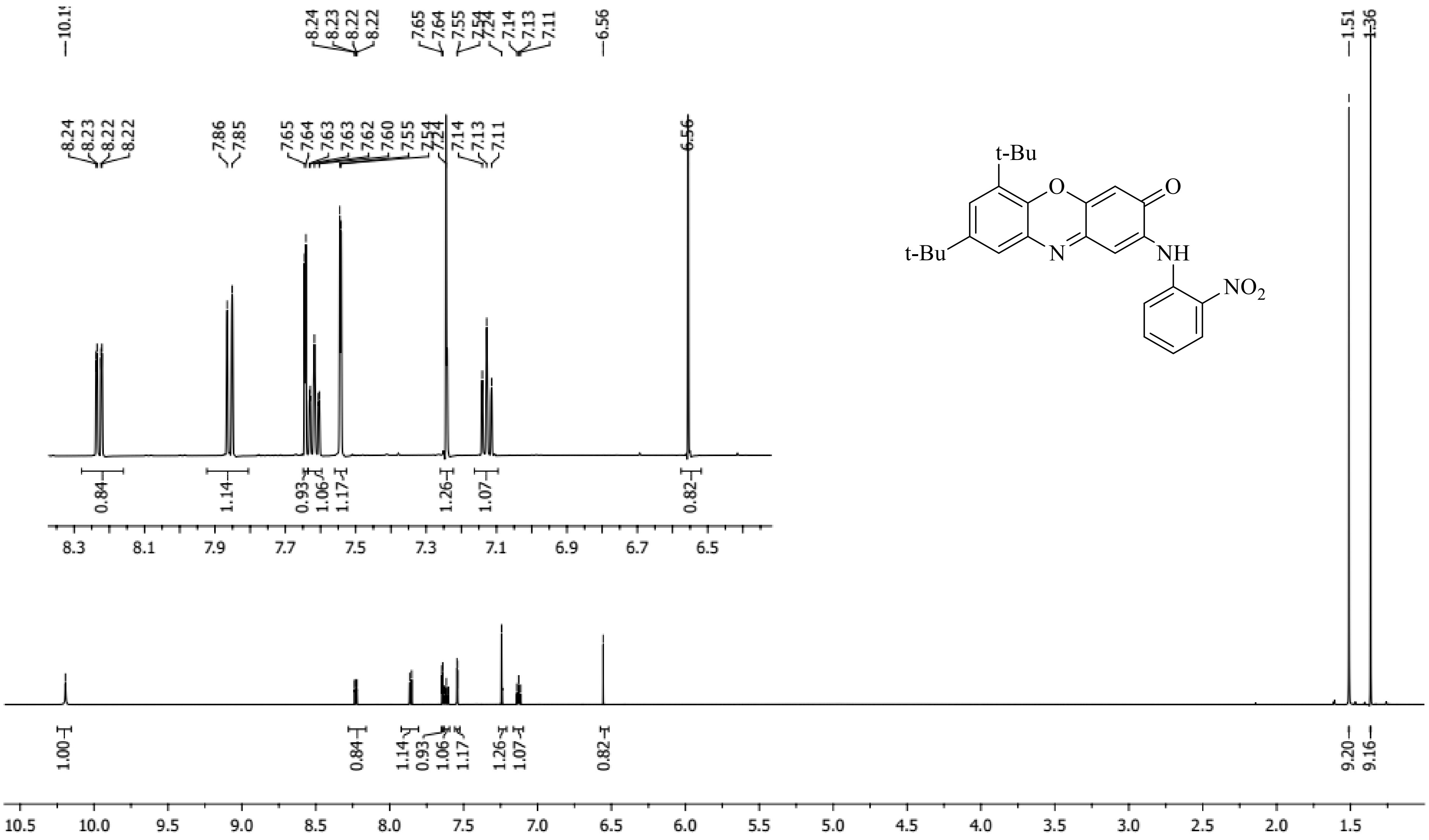

Figure S23: ^{1}H NMR spectrum of 6,8-di-*tert*-butyl-2-((2-nitrophenyl)amino)-3*H*-phenoxazin-3-one (**4f**).

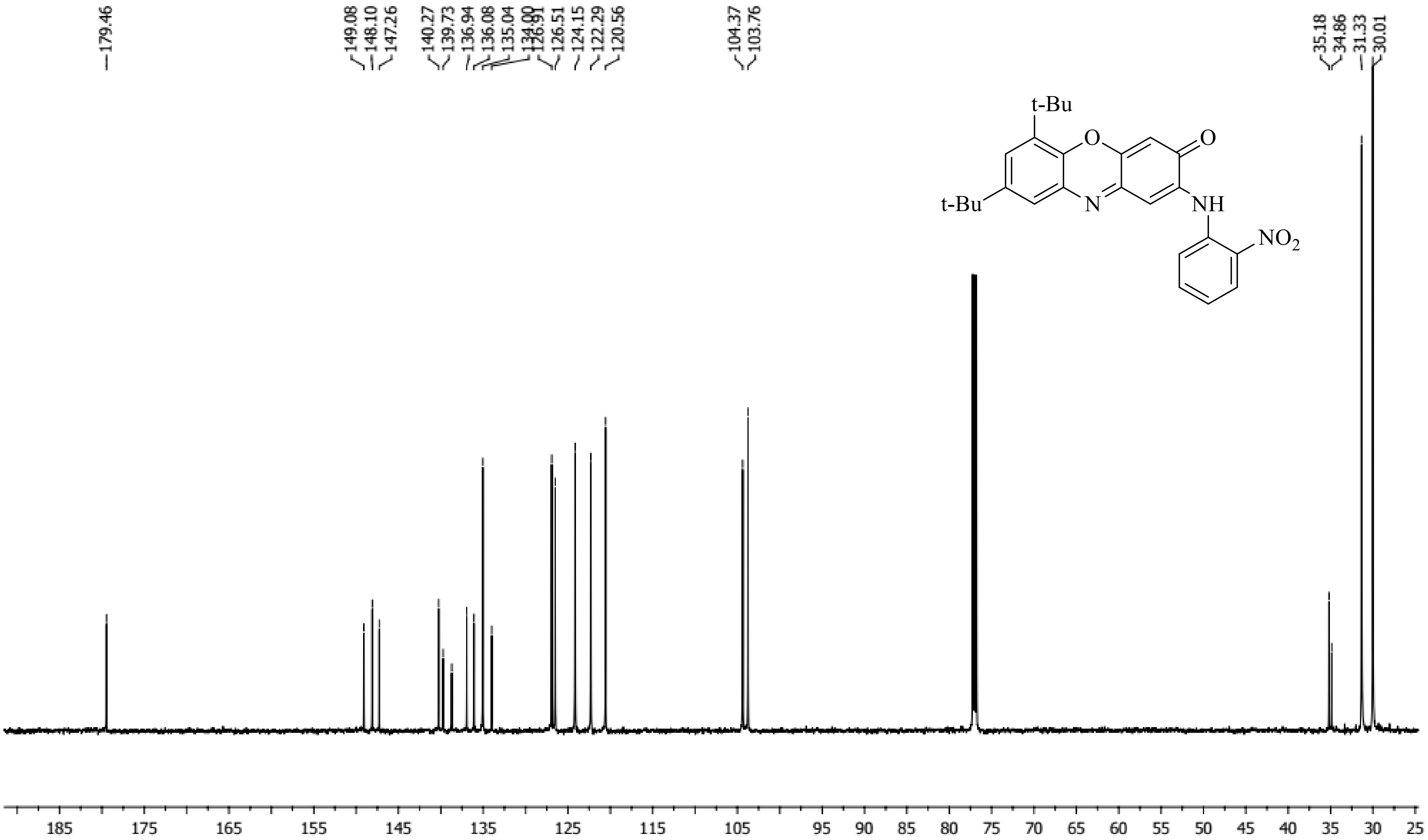

Figure S24: ^{13}C NMR spectrum of 6,8-di-*tert*-butyl-2-((2-nitrophenyl)amino)-3*H*-phenoxazin-3-one (**4f**).

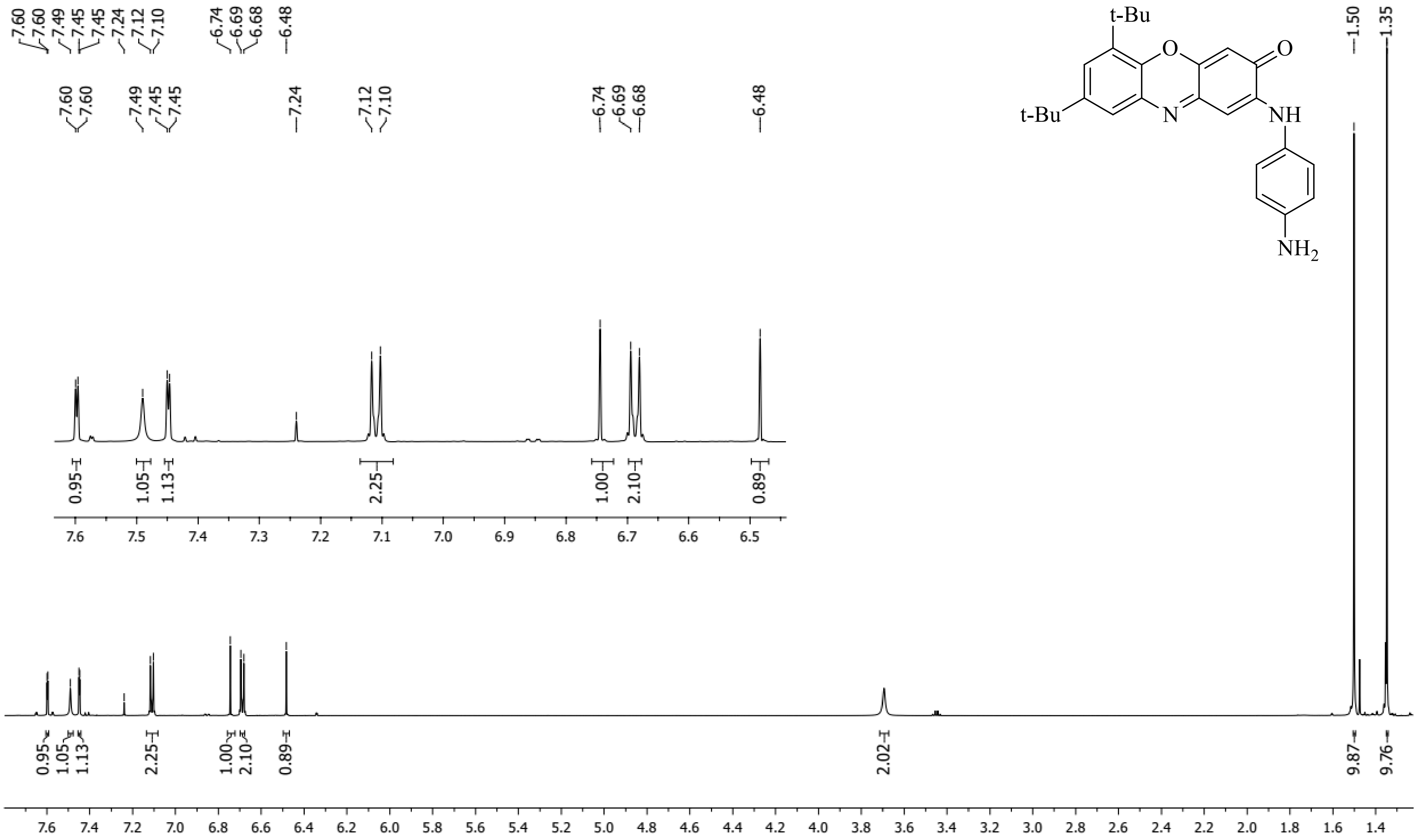

Figure S25: ^{1}H NMR spectrum of 2-((4-aminophenyl)amino)-6,8-di-*tert*-butyl-3*H*-phenoxazin-3-one (**4g**).

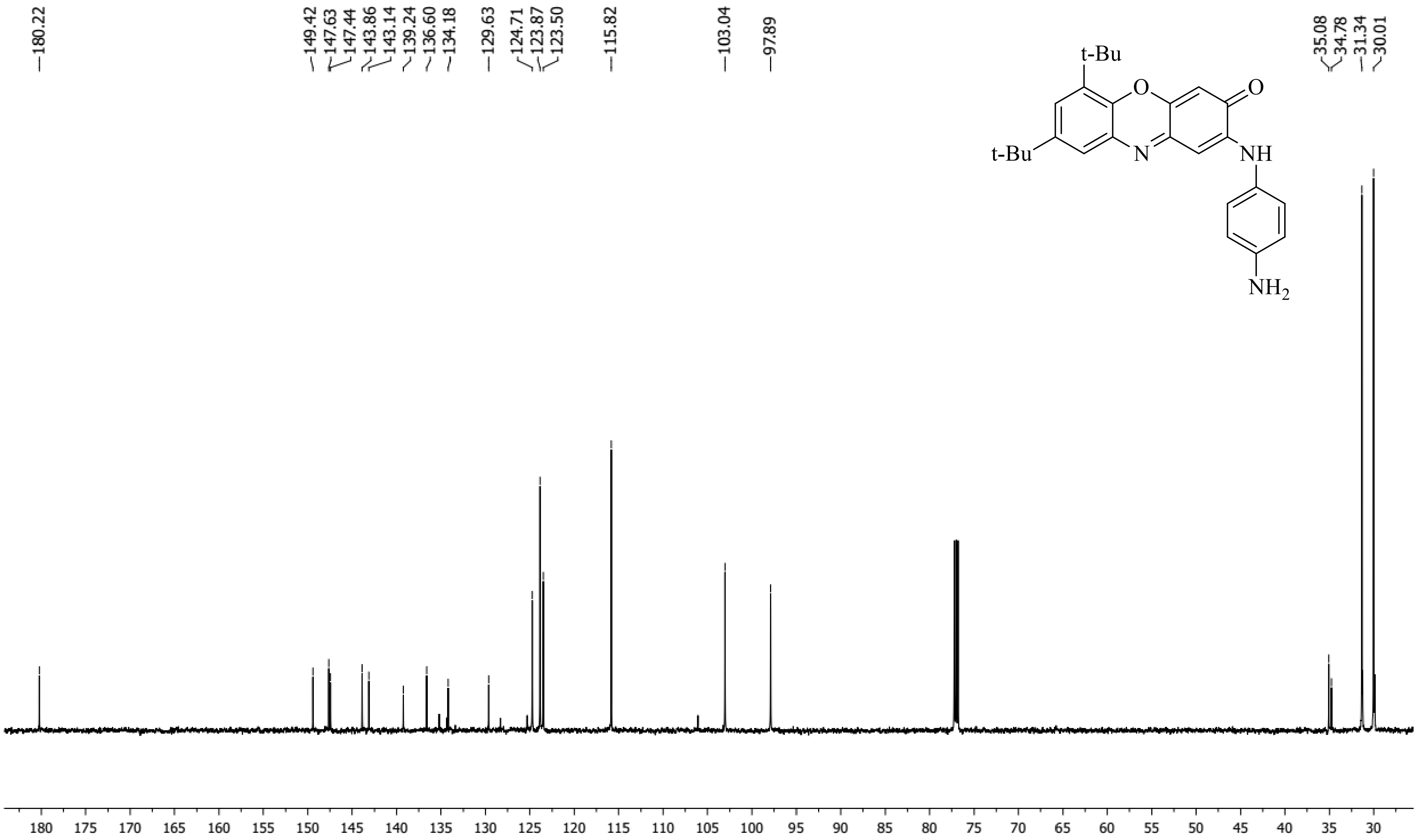

Figure S26: ^{13}C NMR spectrum of 2-((4-aminophenyl)amino)-6,8-di-*tert*-butyl-3*H*-phenoxazin-3-one (**4g**).

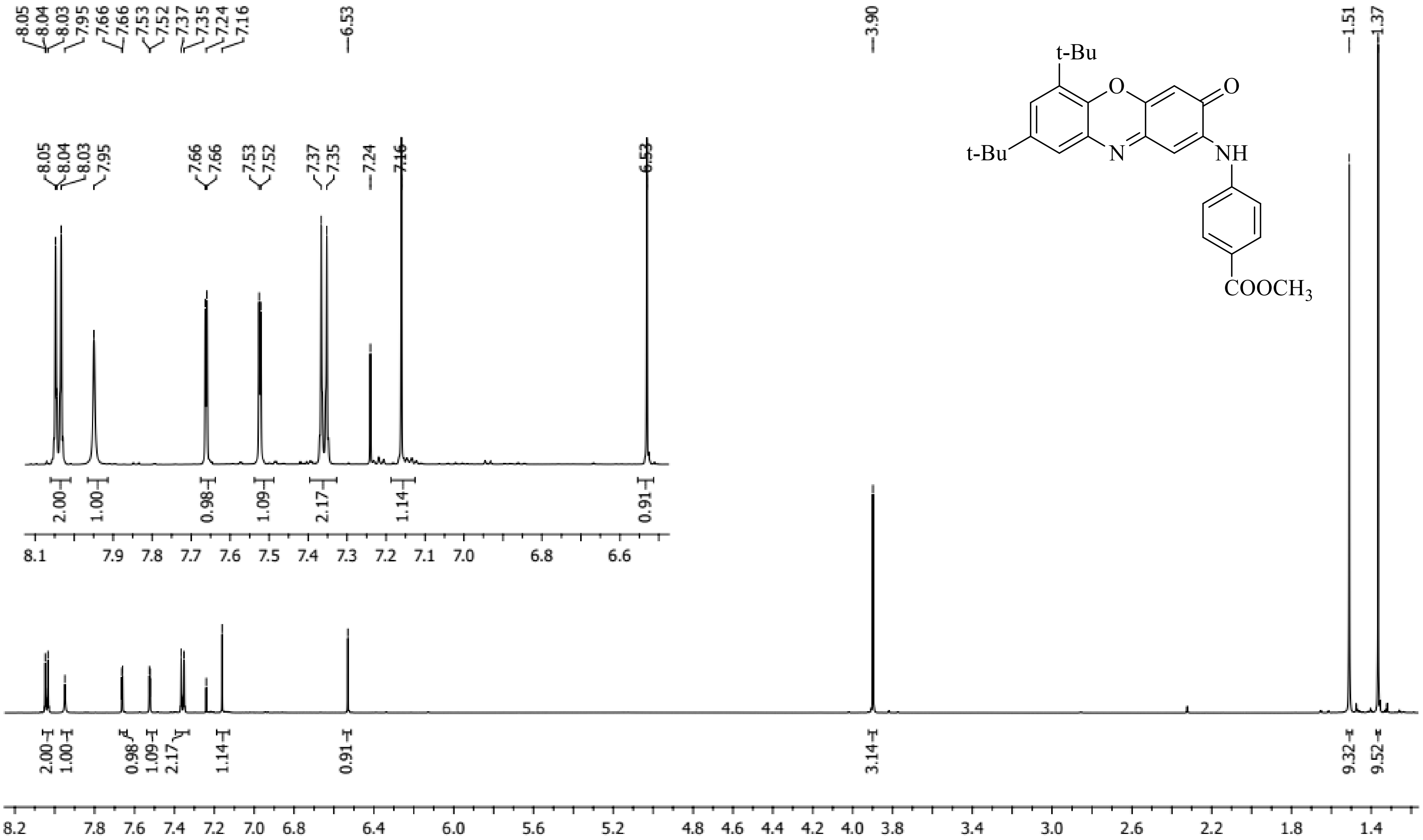

Figure S27: ^{1}H NMR spectrum of methyl 4-((6,8-di-*tert*-butyl-3-oxo-3*H*-phenoxazin-2-yl)amino)benzoate (**4h**).

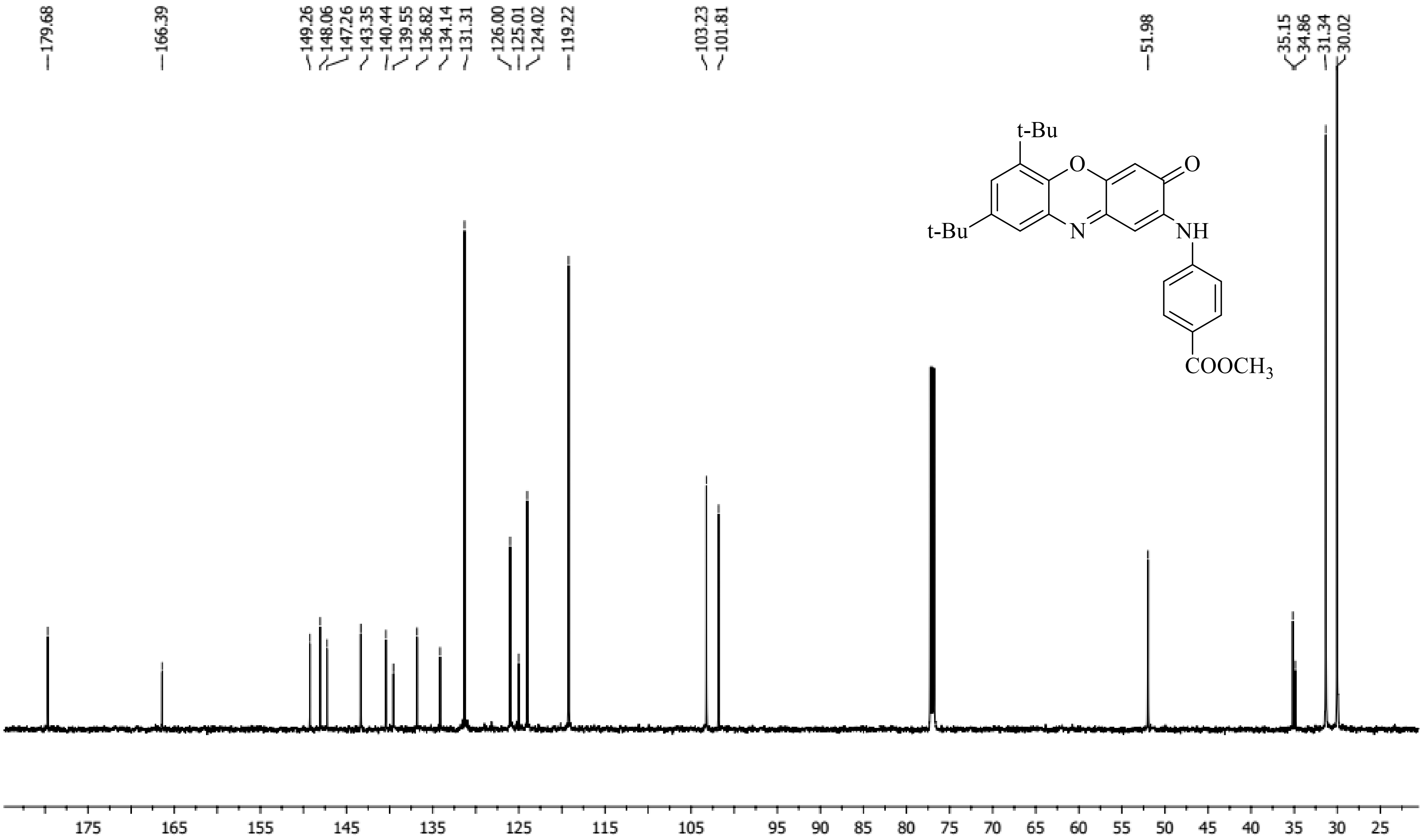

Figure S28: ^{13}C NMR spectrum of methyl 4-((6,8-di-*tert*-butyl-3-oxo-3*H*-phenoxazin-2-yl)amino)benzoate (**4h**).

2,4-Di-*tert*-butyl-14*H*-quinoxalino[2,3-*b*]phenoxazine (**5a**)

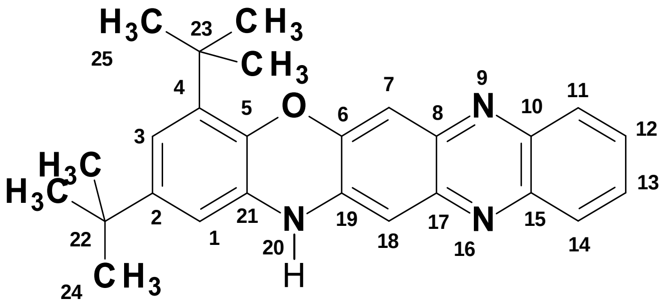

Table S9: Chemical shifts ^{1}H, ^{13}C, and ^{15}N of compound **5a** in $CDCl_3$ at 30°C δ (ppm) and spin–spin coupling constants *J* (Hz).

Compound	**7.** Nucleus	1	2	3	4	5	6	7	8	9(^{15}N)	10	11	12	13	14
**5a**	^{1}H	6.42		6.74				7.26				7.96	7.48	7.48	7.87
	$J_{1H\text{-}1H}$	2.4		2.4								7.8	7.8	7.8	7.8
	**8.** $^{13}C/^{15}N$	109.64	136.94	117.04	146.46	138.09	144.11	109.69	143.58	314.95	141.67	128.98	128.02	129.10	127.28

Compound	**9.** Nucleus	15	16(^{15}N)	17	18	19	20(^{15}N)	21	22	23	24	25
**5a**	^{1}H				6.85						1.46	1.13
	$J_{1H\text{-}1H}$											
	**10.** $^{13}C/^{15}N$	142.19	296.56	148.97	102.35	136.53	92.79	127.80	34.93	34.38	29.98	31.20

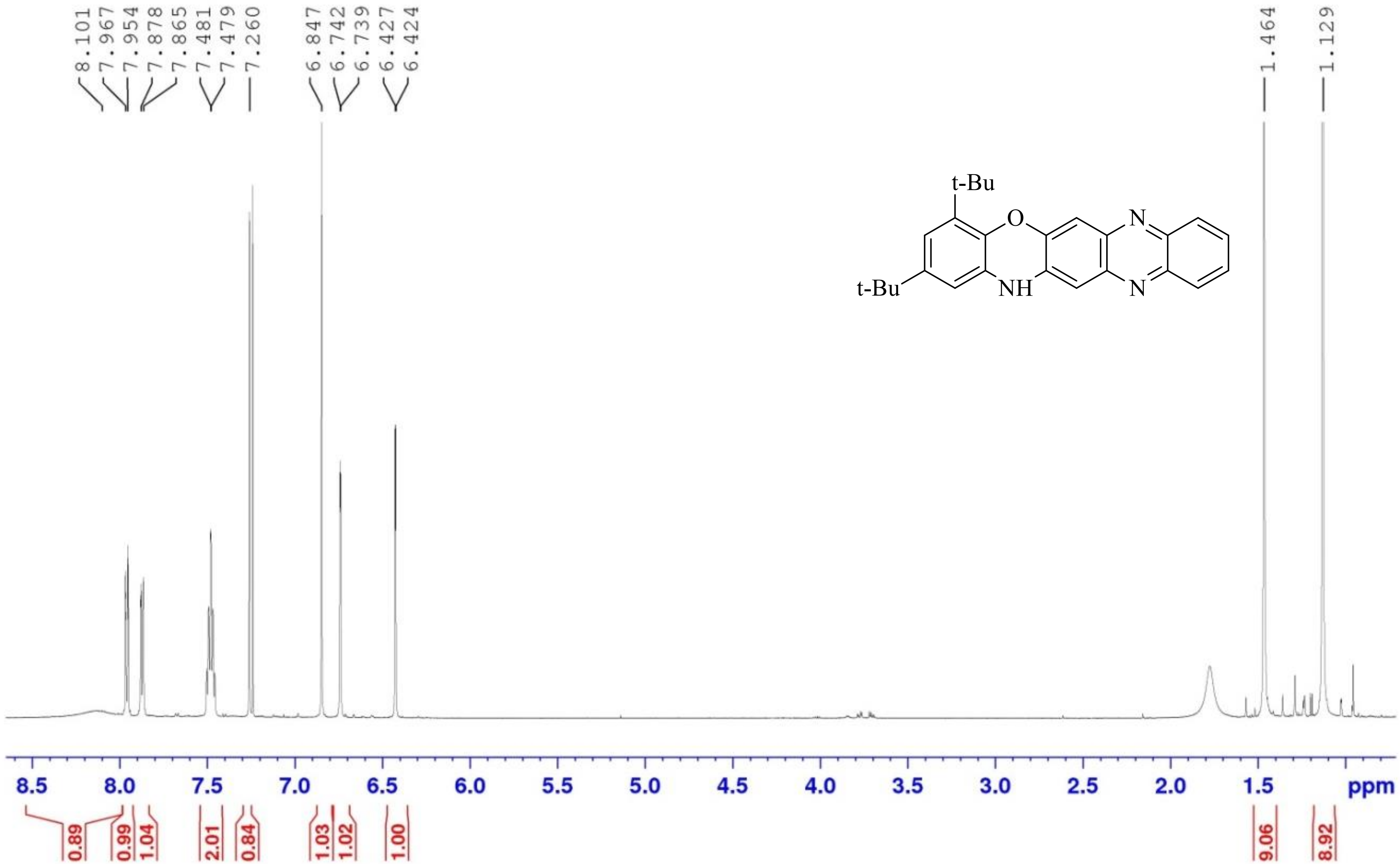

Figure S29: ^{1}H NMR spectrum of 2,4-di-*tert*-butyl-14*H*-quinoxalino[2,3-*b*]phenoxazine (**5a**).

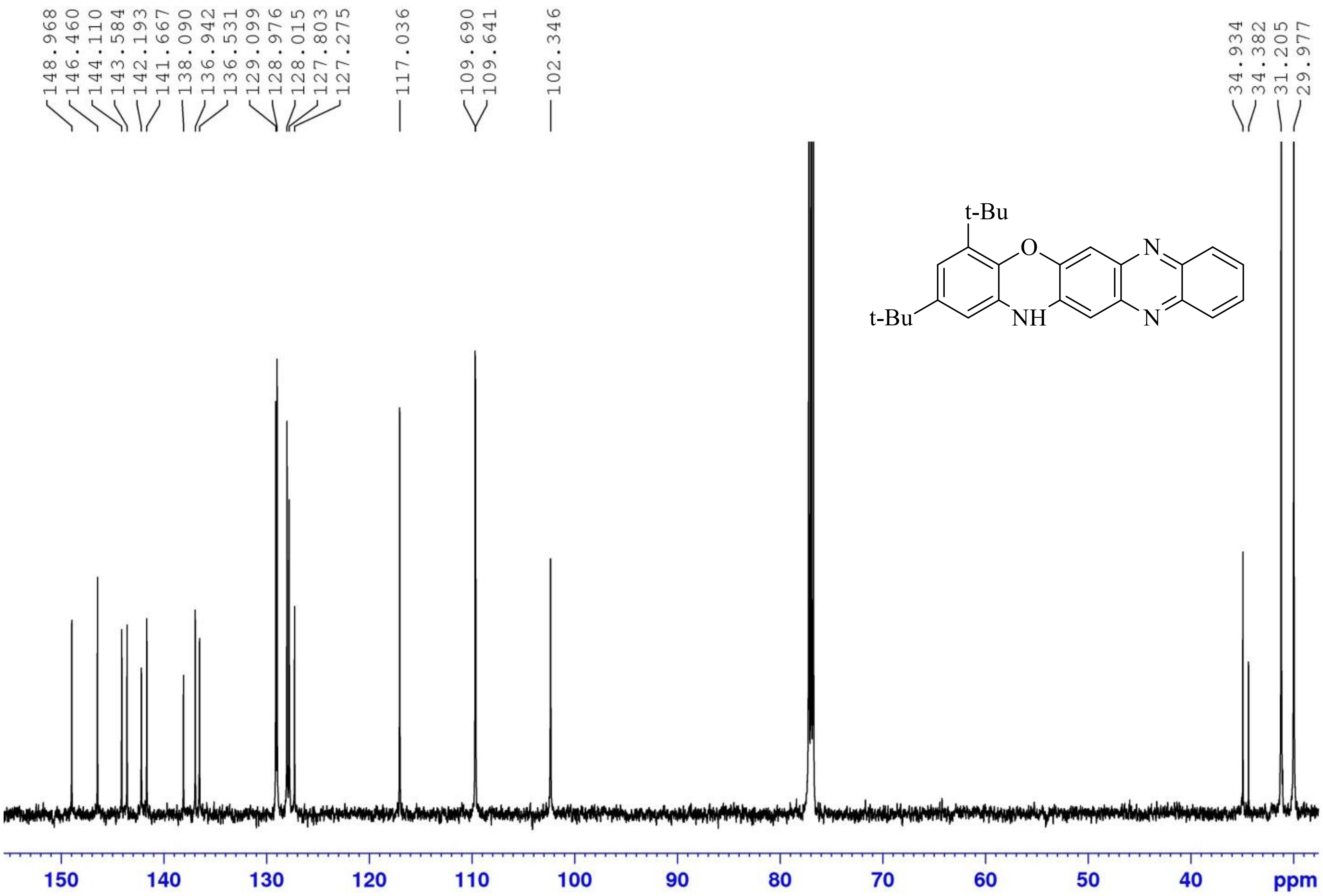

Figure S30: ^{13}C NMR spectrum of 2,4-di-*tert*-butyl-14*H*-quinoxalino[2,3-*b*]phenoxazine (**5a**).

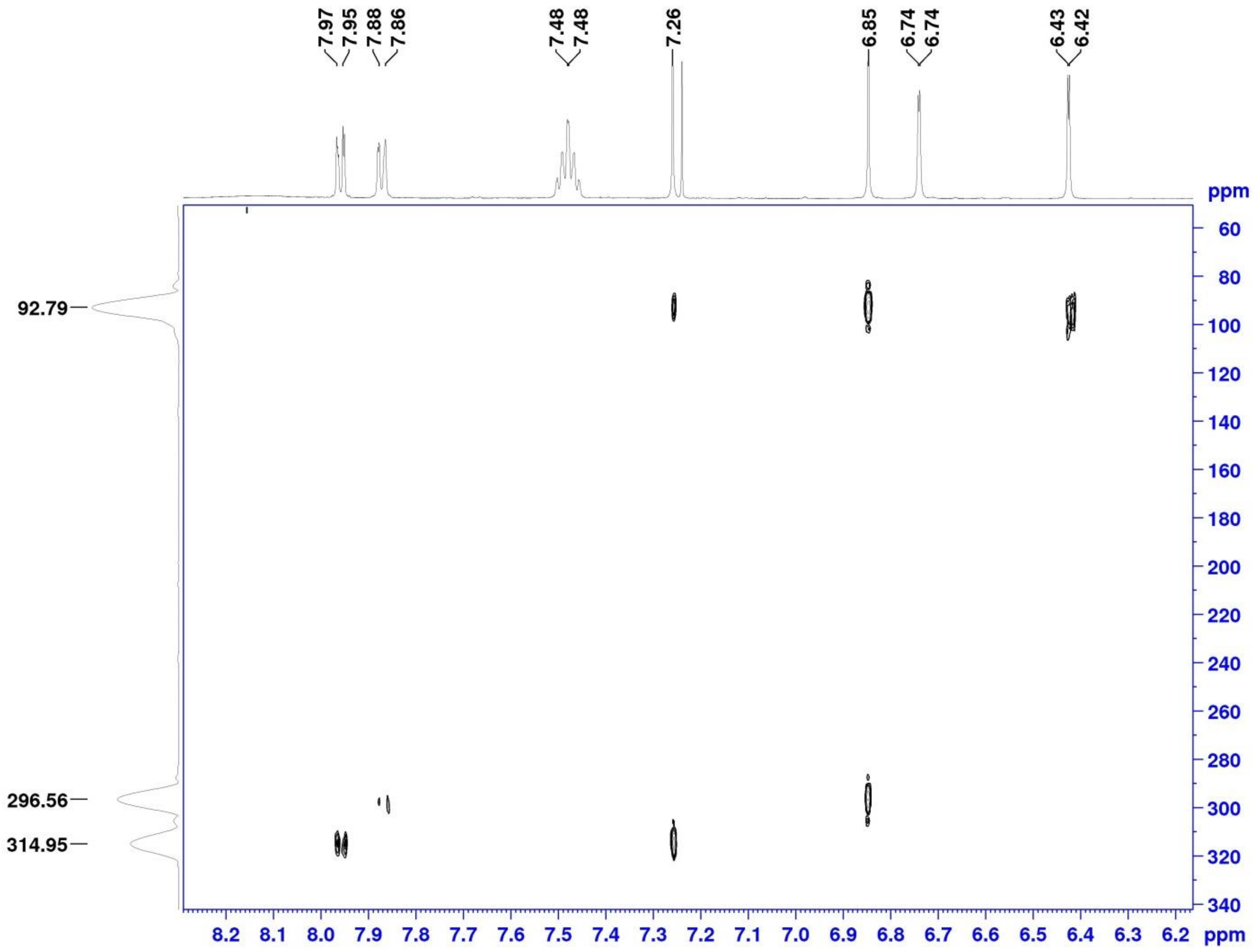

Figure S31: HMBC ^{1}H,^{15}N NMR spectrum of 2,4-di-*tert*-butyl-14*H*-quinoxalino[2,3-*b*]phenoxazine (**5a**).

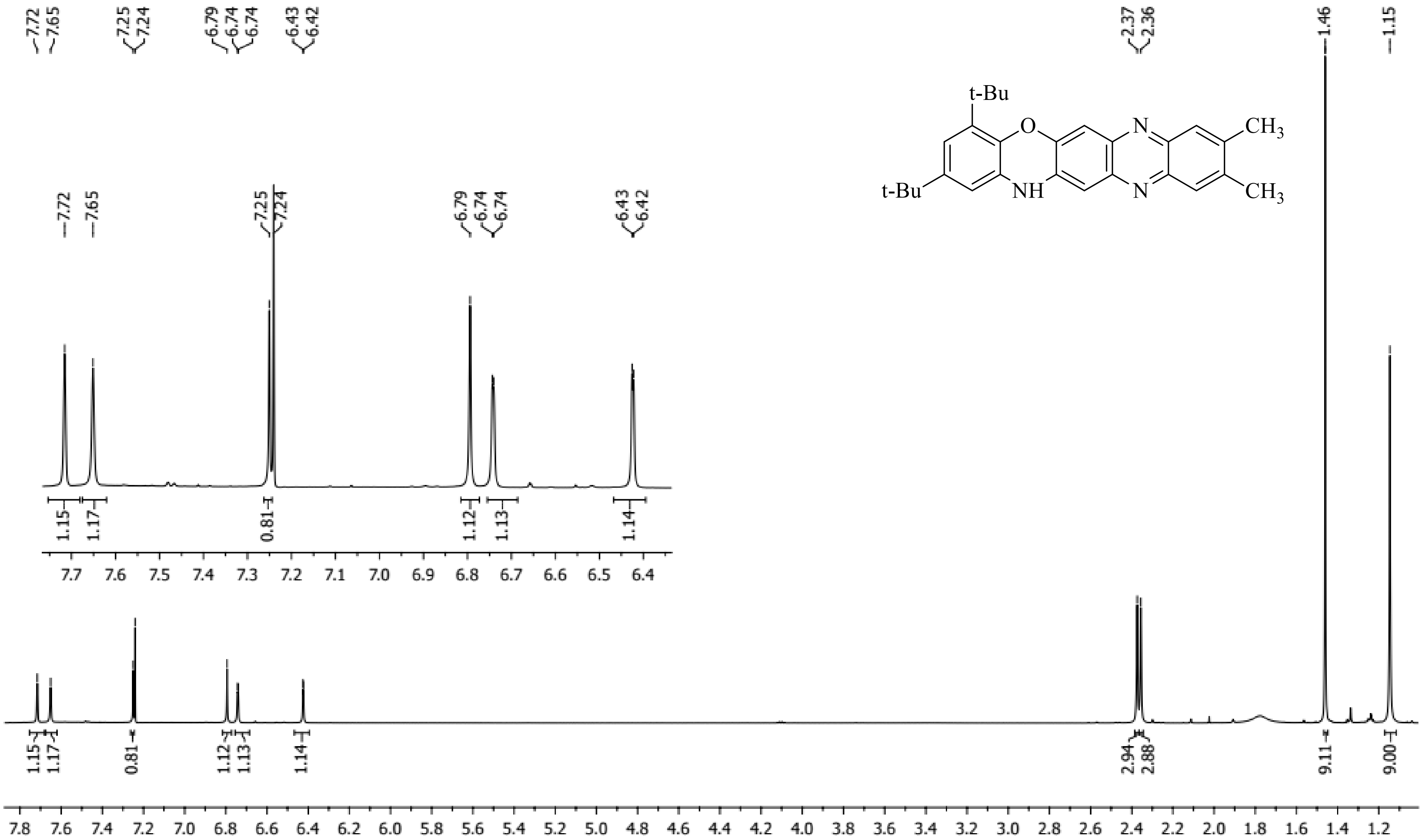

Figure S32: ^{1}H NMR spectrum of 2,4-di-*tert*-butyl-9,10-dimethyl-14*H*-quinoxalino[2,3-*b*]phenoxazine (**5b**).

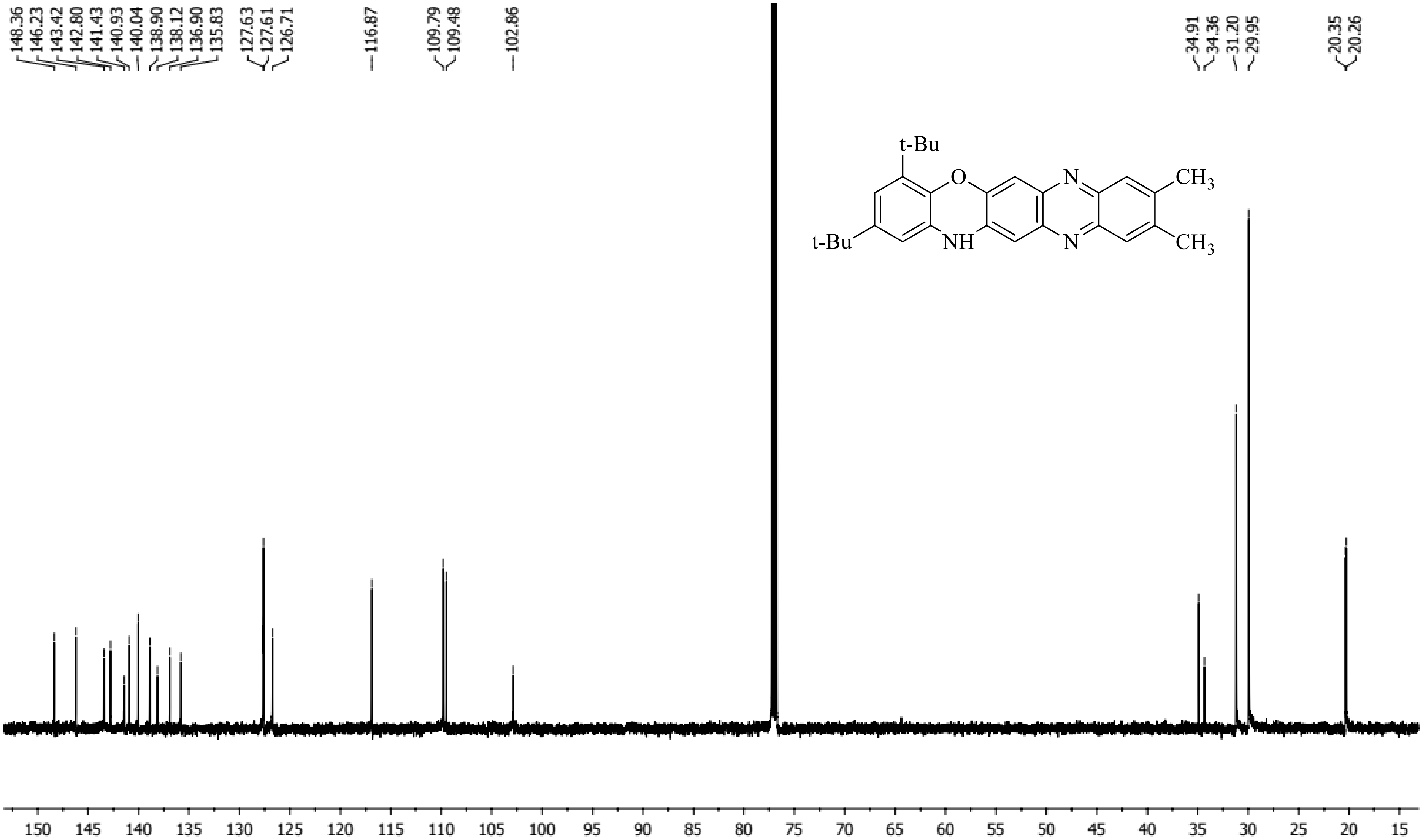

Figure S33: ^{13}C NMR spectrum of 2,4-di-*tert*-butyl-9,10-dimethyl-14*H*-quinoxalino[2,3-*b*]phenoxazine (**5b**).

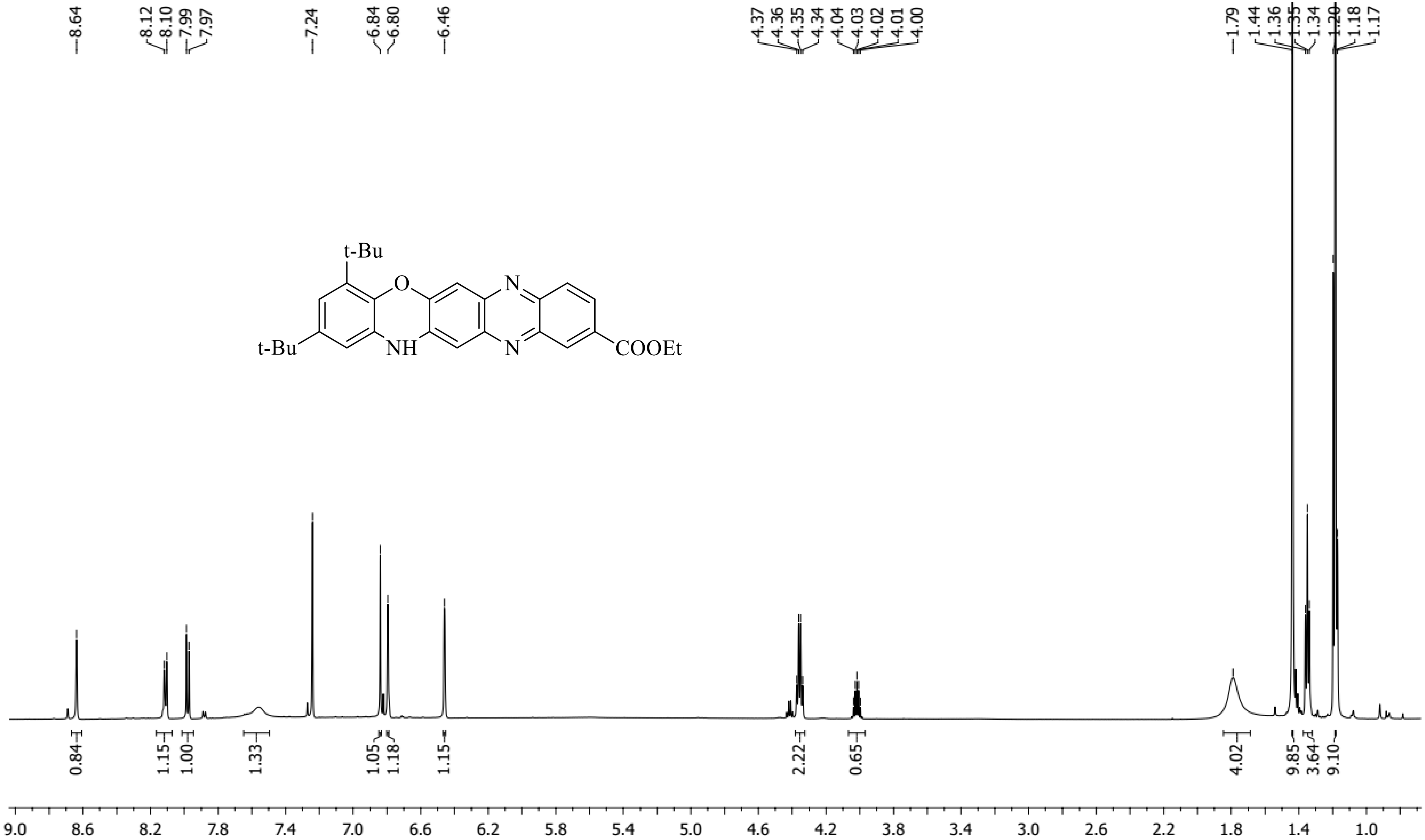

Figure S34: ^{1}H NMR spectrum of ethyl 2,4-di-*tert*-butyl-14*H*-quinoxalino[2,3-*b*]phenoxazine-10-carboxylate (**5c**).

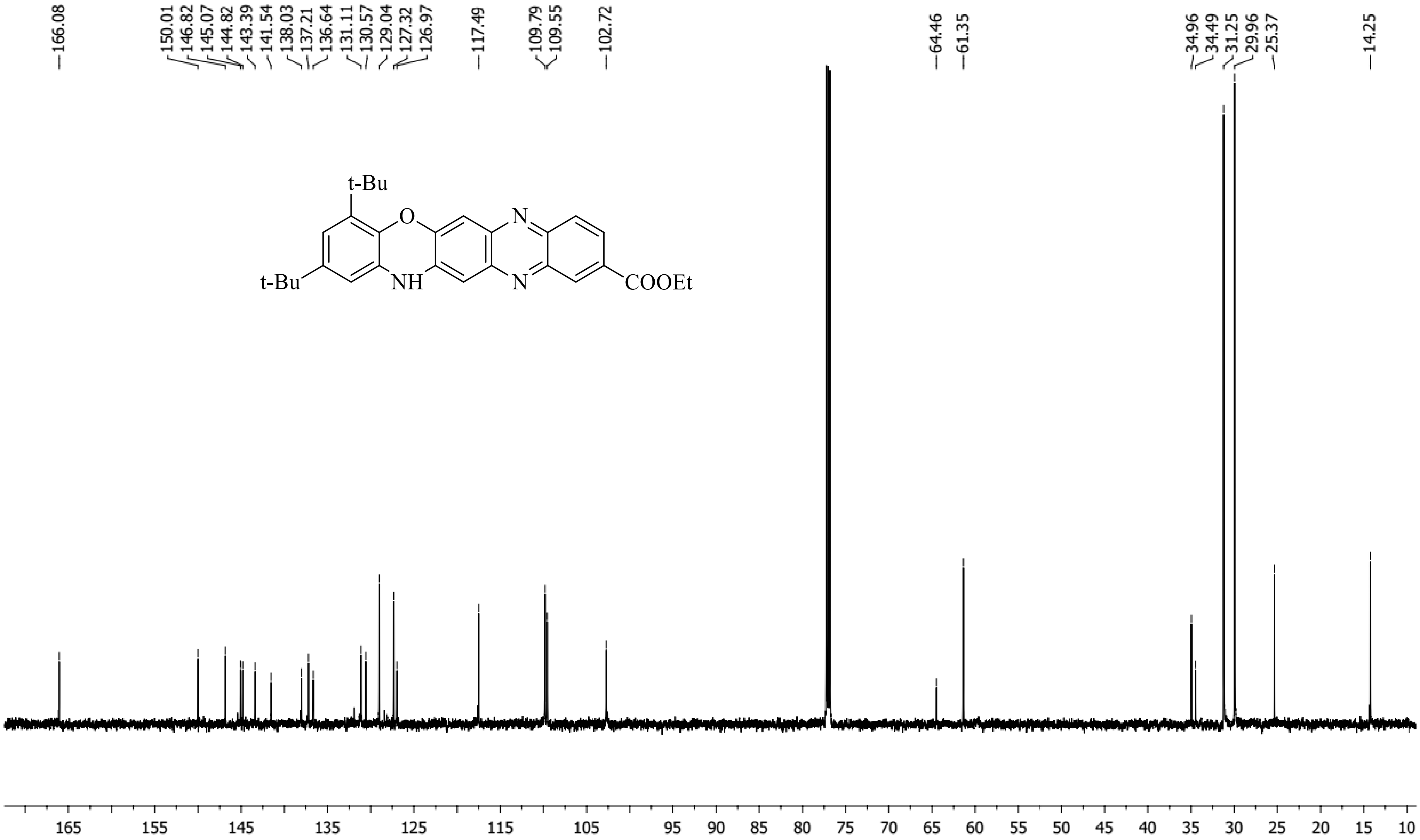

Figure S35: ^{13}C NMR spectrum of ethyl 2,4-di-*tert*-butyl-14*H*-quinoxalino[2,3-*b*]phenoxazine-10-carboxylate (**5c**).

2,4-Di-*tert*-butyl-14-methyl-14H-quinoxalino[2,3-*b*]phenoxazine (**6a**)

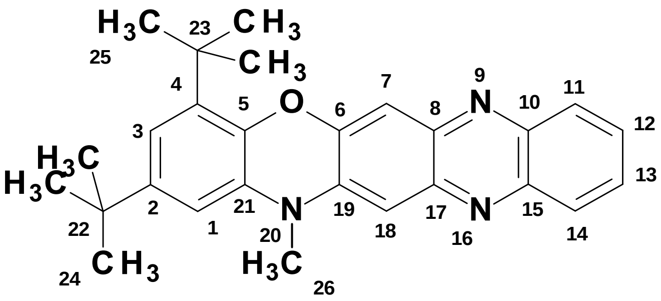

Table S10: Chemical shifts ^{1}H, ^{13}C, and ^{15}N of compound **6a** in $CDCl_3$ at 30°C δ (ppm) and spin–spin coupling constants *J* (Hz).

Compound	**11.** Nucleus	1	2	3	4	5	6	7	8	9(^{15}N)	10	11	12	13	14
**6a**	^{1}H	6.71		6.93				7.34				8.01	7.60	7.63	7.98
	J_{1H-1H}	2.1		2.1								8.1	8.1	8.1	8.1
	**12.** $^{13}C/^{15}N$	108.55	136.92	117.35	146.30	140.04	144.48	109.17	143.17	311.64	141.90	128.90	128.31	129.07	128.49

Compound	**13.** Nucleus	15	16(^{15}N)	17	18	19	20(^{15}N)	21	22	23	24	25	26
**6a**	^{1}H				6.94						1.46	1.31	3.37
	J_{1H-1H}												
	**14.** $^{13}C/^{15}N$	142.70	305.64	150.45	103.54	139.19	83.69	130.84	34.83	34.97	30.03	31.45	32.45

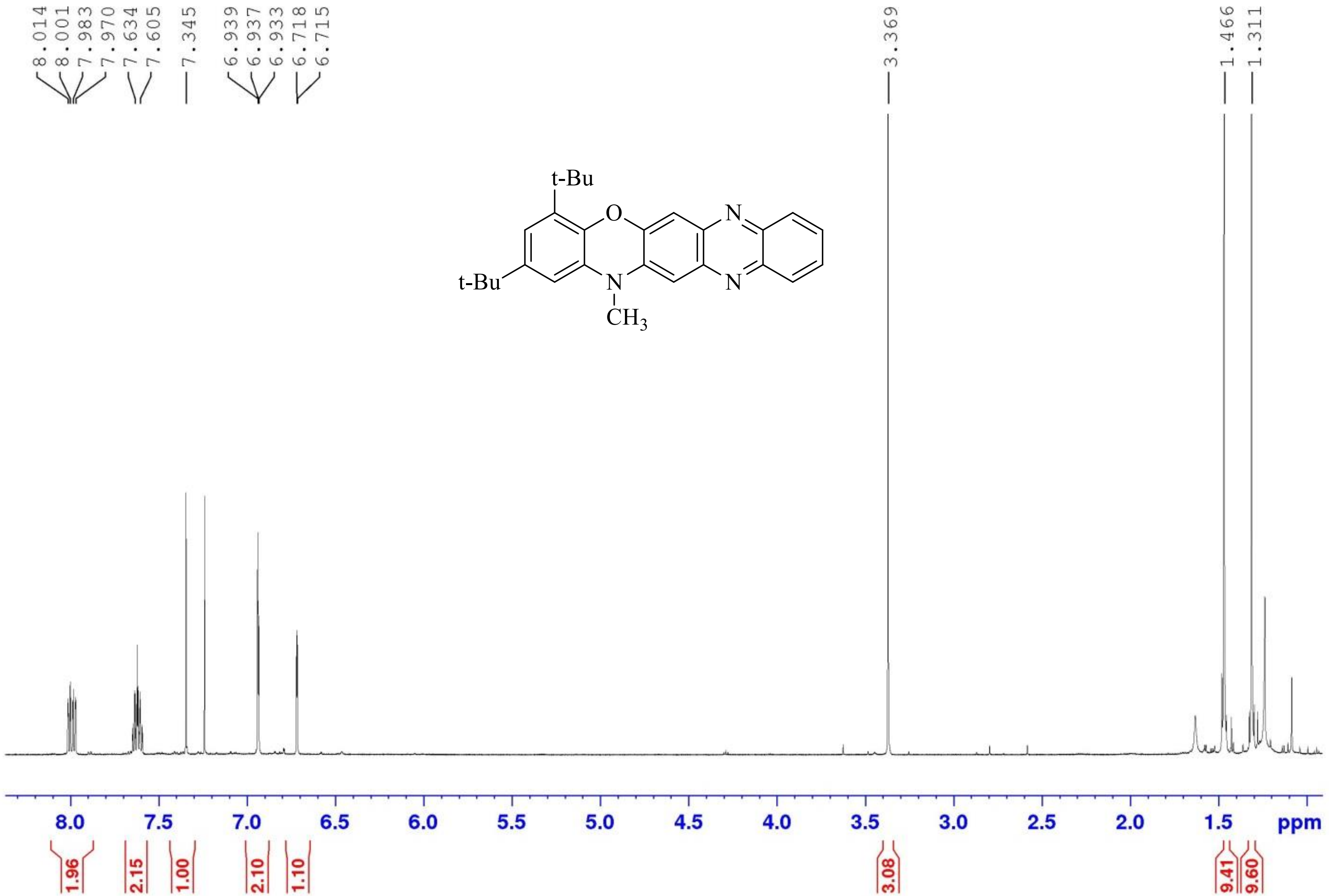

Figure S36: ^{1}H NMR spectrum of 2,4-di-*tert*-butyl-14-methyl-14*H*-quinoxalino[2,3-*b*]phenoxazine (**6a**).

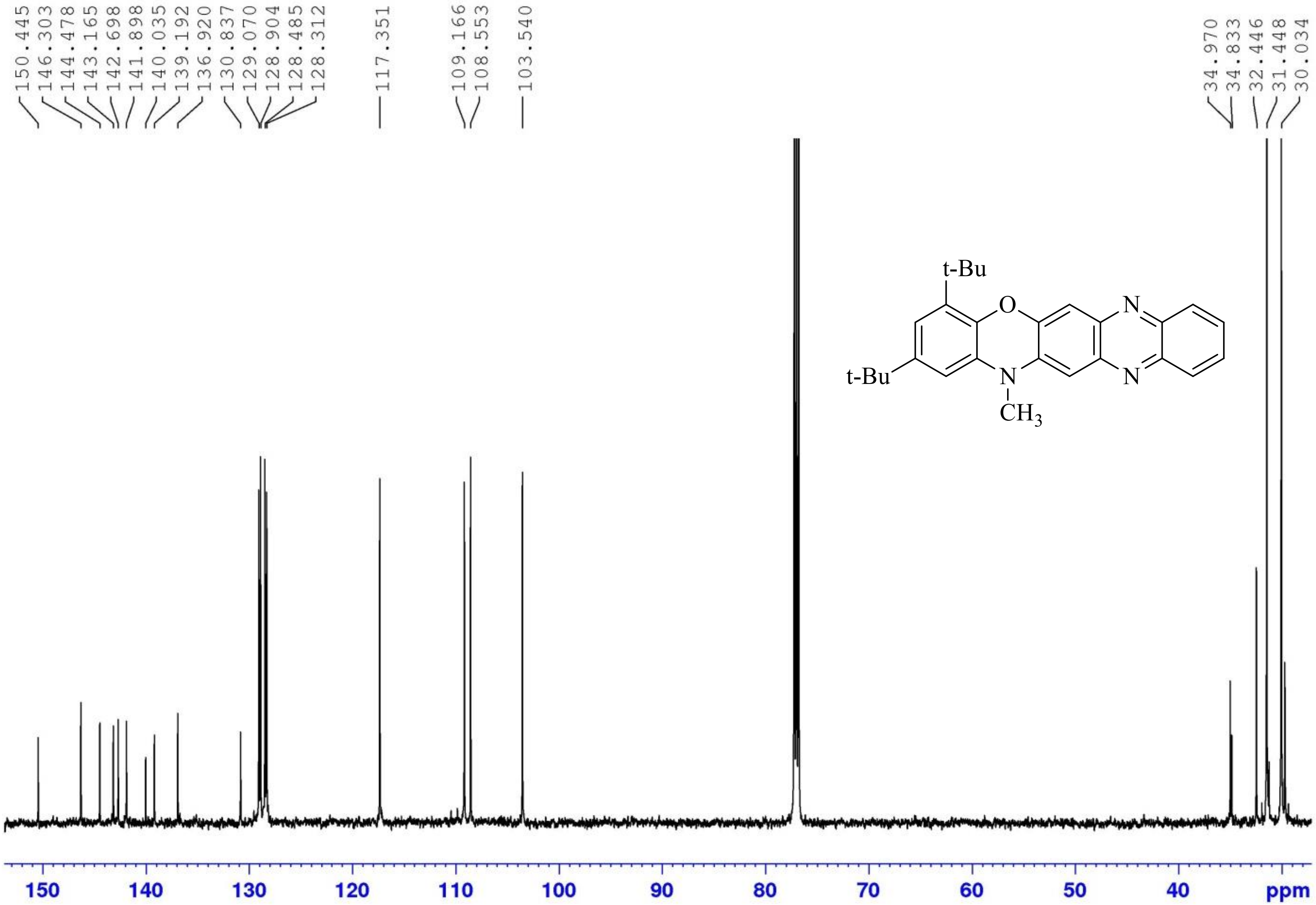

Figure S37: ^{13}C NMR spectrum of 2,4-di-*tert*-butyl-14-methyl-14*H*-quinoxalino[2,3-*b*]phenoxazine (**6a**).

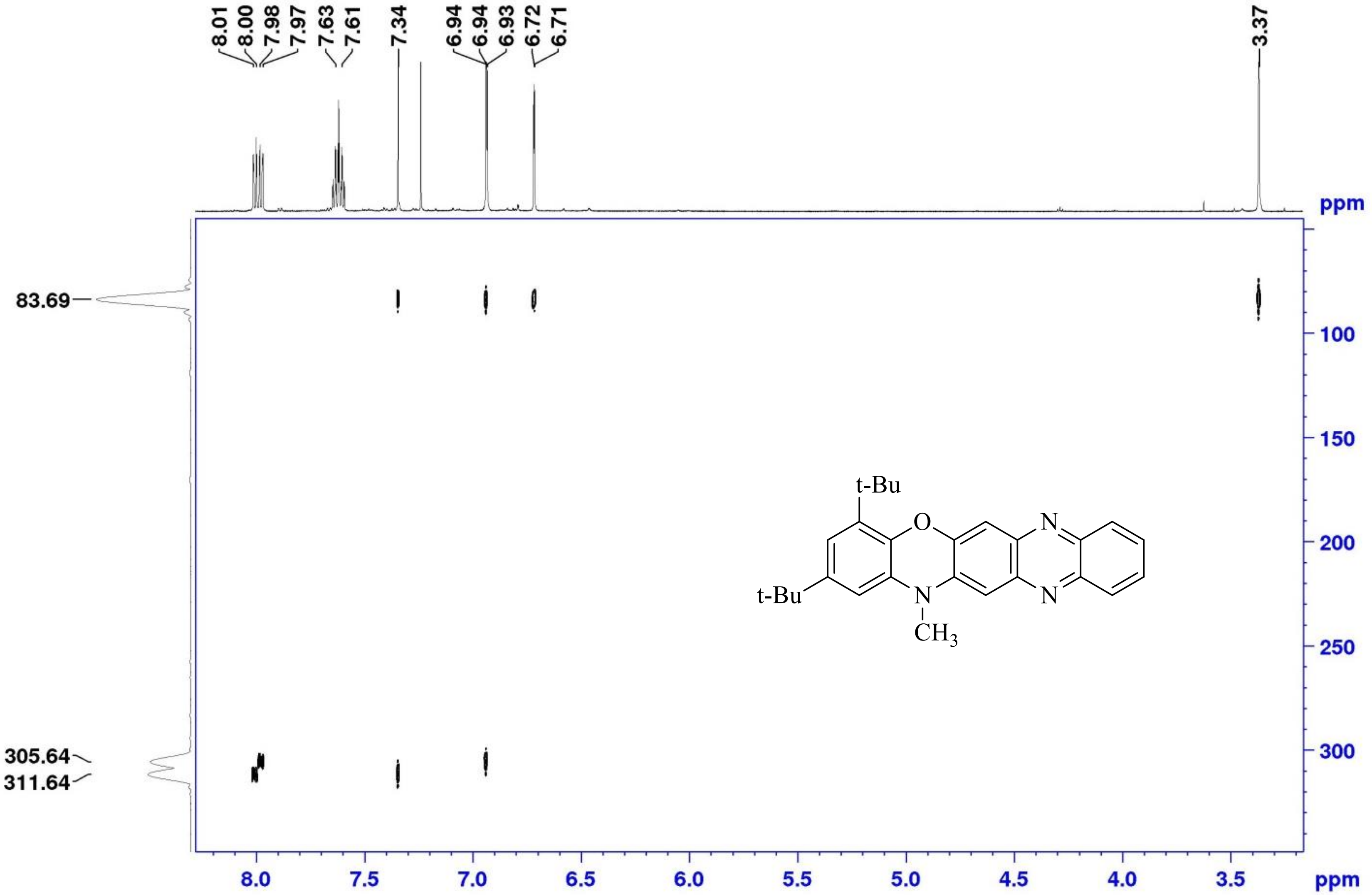

Figure S38: HMBC $^{1}H,^{15}N$ NMR spectrum of 2,4-di-*tert*-butyl-14-methyl-14*H*-quinoxalino[2,3-*b*]phenoxazine (**6a**).

2,4-Di-*tert*-butyl-14-nonyl-14*H*-quinoxalino[2,3-*b*]phenoxazine (**6b**)

2,4-Di-*tert*-butyl-14-nonyl-14*H*-quinoxalino[2,3-*b*]phenoxazine (**6b**)

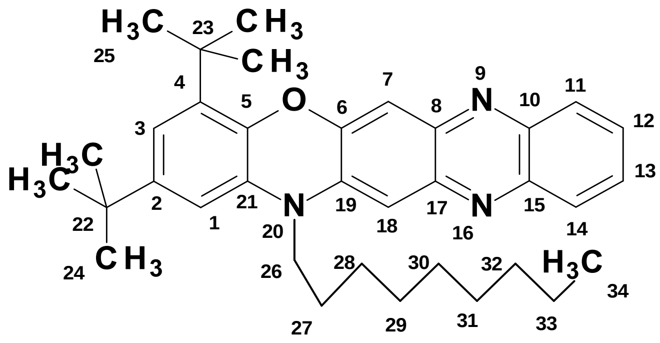

Table S11: Chemical shifts ^{1}H, ^{13}C, and ^{15}N of compound **6b** in $CDCl_3$ at 30°C δ (ppm) and spin–spin coupling constants *J* (Hz).

Compound	**15.** Nucleus	1	2	3	4	5	6	7	8	9(^{15}N)	10	11	12	13	14
**6b**	^{1}H	6.70		6.90				7.29				7.98	7.59	7.63	7.97
	J_{1H-1H}	2.1		2.1								8.1	8.1	8.1	8.1
	**16.** $^{13}C/^{15}N$	108.41	136.82	117.25	146.04	139.44	144.76	108.99	143.16	310.36	141.91	128.89	128.17	129.08	129.02

Compound	**17.** Nucleus	15	16(^{15}N)	17	18	19	20(^{15}N)	21	22	23	24	25
**6b**	^{1}H				6.91						1.45	1.31
	J_{1H-1H}											
	**18.** $^{13}C/^{15}N$	142.74	303.85	149.87	102.76	137.50	96.38	129.19	34.77	34.99	29.99	31.41

Compound	**19.** Nucleus	26	27	28	29	30	31	32	33	34
**6b**	^{1}H	3.79	1.85	1.53	1.50	1.42	1.35	1.32	1.28	0.89
	J_{1H-1H}	t,8.3	qvin,7.6							t,7.0
	**20.** $^{13}C/^{15}N$	45.61	24.74	26.97	31.83	29.54	29.39	29.21	22.65	14.07

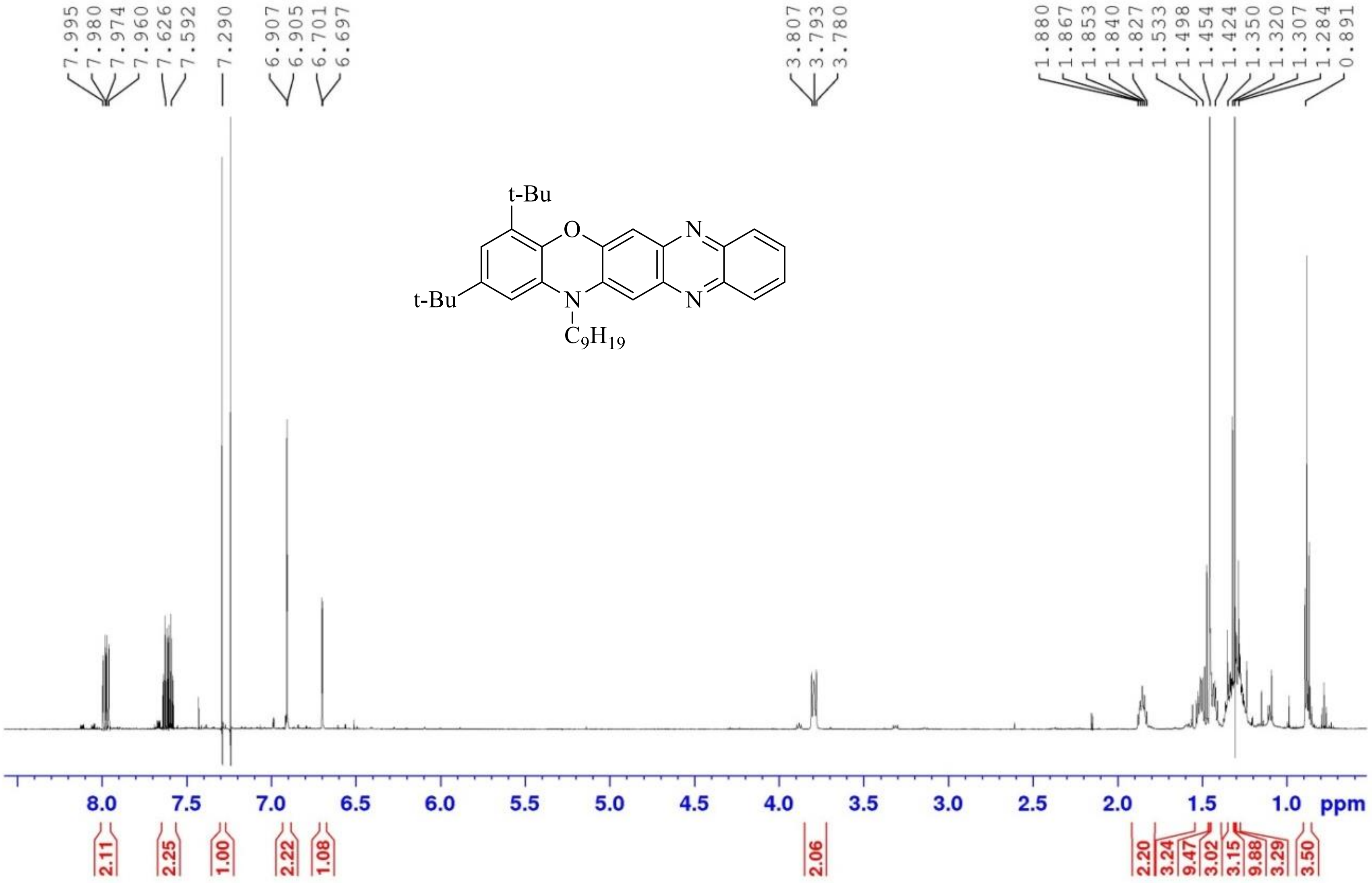

Figure S39: ^{1}H NMR spectrum of 2,4-di-*tert*-butyl-14-nonyl-14*H*-quinoxalino[2,3-*b*]phenoxazine (**6b**).

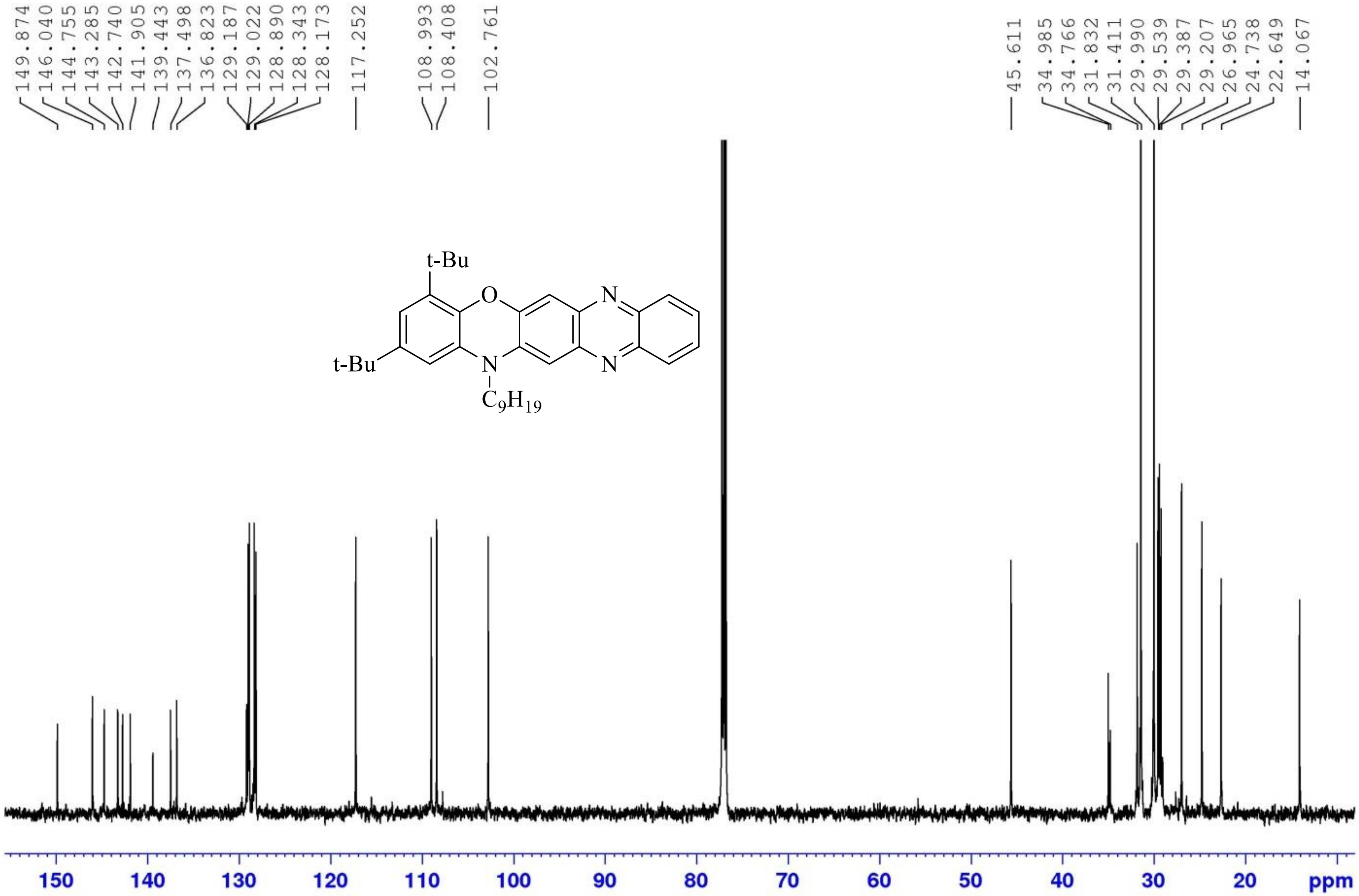

Figure S40: ^{13}C NMR spectrum of 2,4-di-*tert*-butyl-14-nonyl-14*H*-quinoxalino[2,3-*b*]phenoxazine (**6b**).

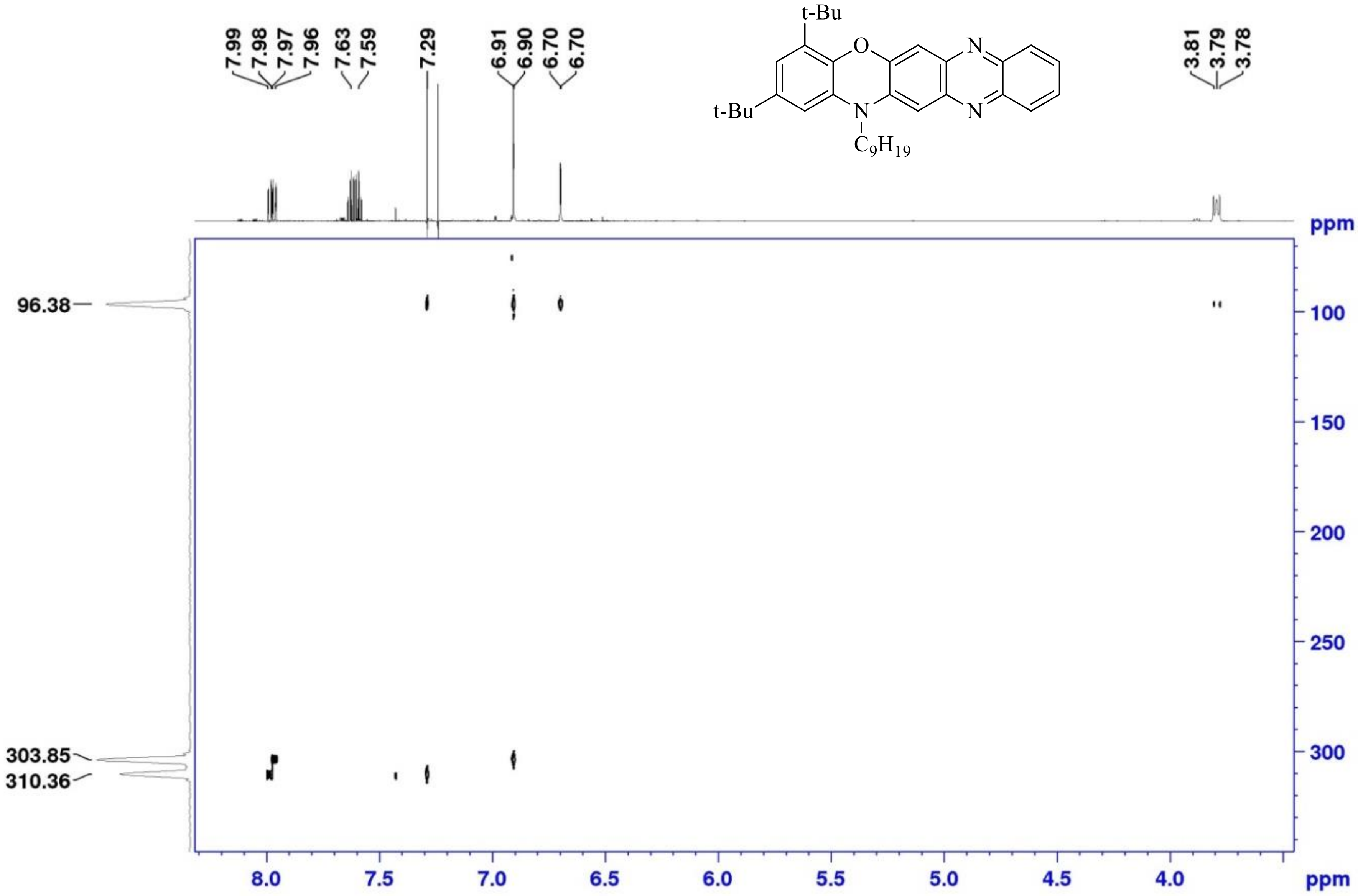

Figure S41: HMBC $^{1}H,^{15}N$ NMR spectrum of 2,4-di-*tert*-butyl-14-nonyl-14*H*-quinoxalino[2,3-*b*]phenoxazine (**6b**).

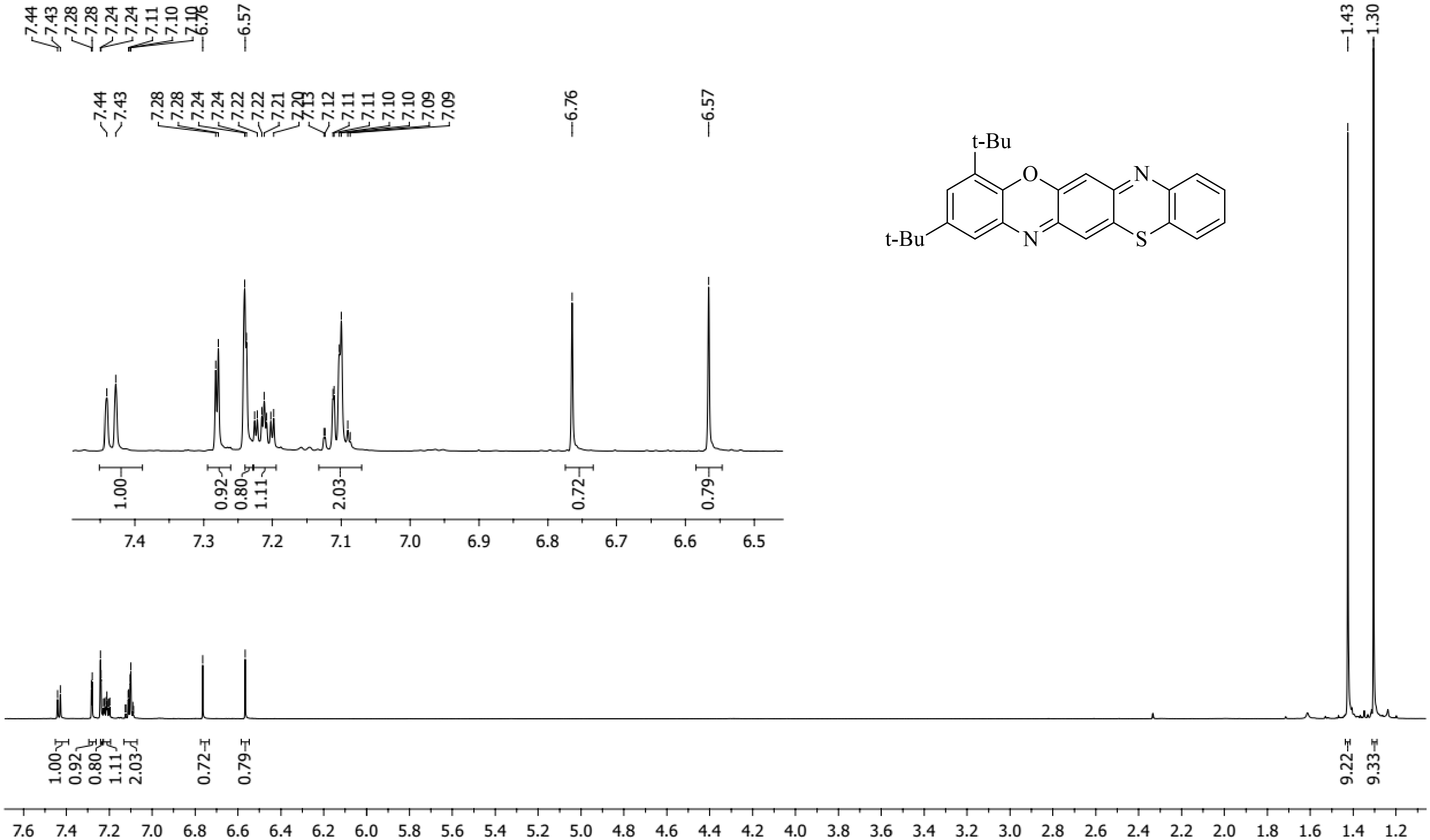

Figure S42: ^{1}H NMR spectrum of 2,4-di-*tert*-butylbenzo[5,6][1,4]oxazino[2,3-*b*]phenothiazine (**10c**).

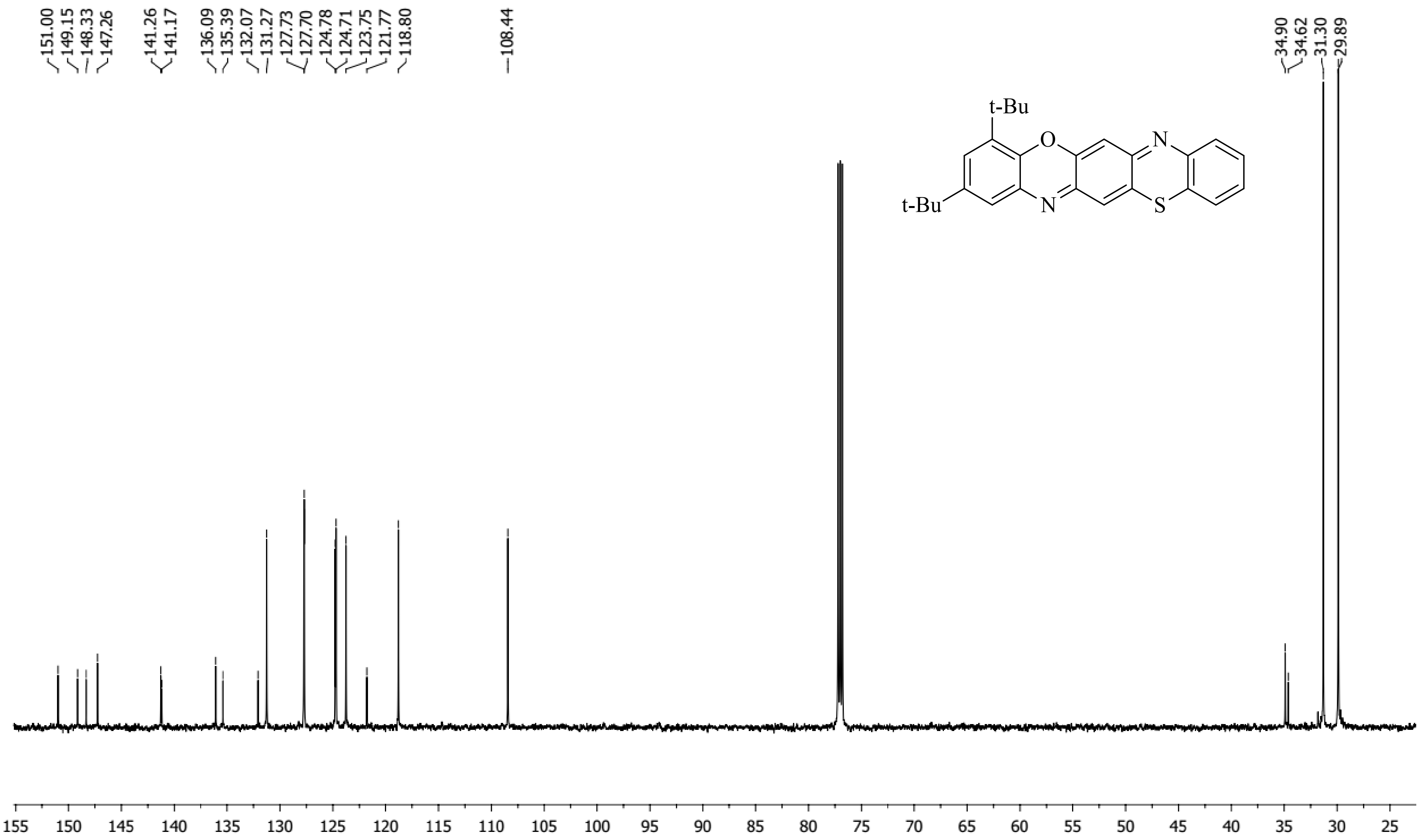

Figure S43: ^{13}C NMR spectrum of 2,4-di-*tert*-butylbenzo[5,6][1,4]oxazino[2,3-*b*]phenothiazine (**10c**).

7. HRMS spectra

Display Report

Analysis Info

Analysis Name	D:\Data\demidov\A7000002.d
Method	Direct_Infusion_50-500_2023_2.m
Sample Name	1
Comment	
Acquisition Date	10/17/2023 2:24:05 PM
Operator	Demidov
Instrument	maXis impact 282001.00109

Acquisition Parameter

Source Type	ESI	Ion Polarity	Positive	Set Nebulizer	0.4 Bar
Focus	Active	Set Capillary	4500 V	Set Dry Heater	180 °C
Scan Begin	50 m/z	Set End Plate Offset	-500 V	Set Dry Gas	4.0 l/min
Scan End	900 m/z	Set Collision Cell RF	500.0 Vpp	Set Divert Valve	Waste

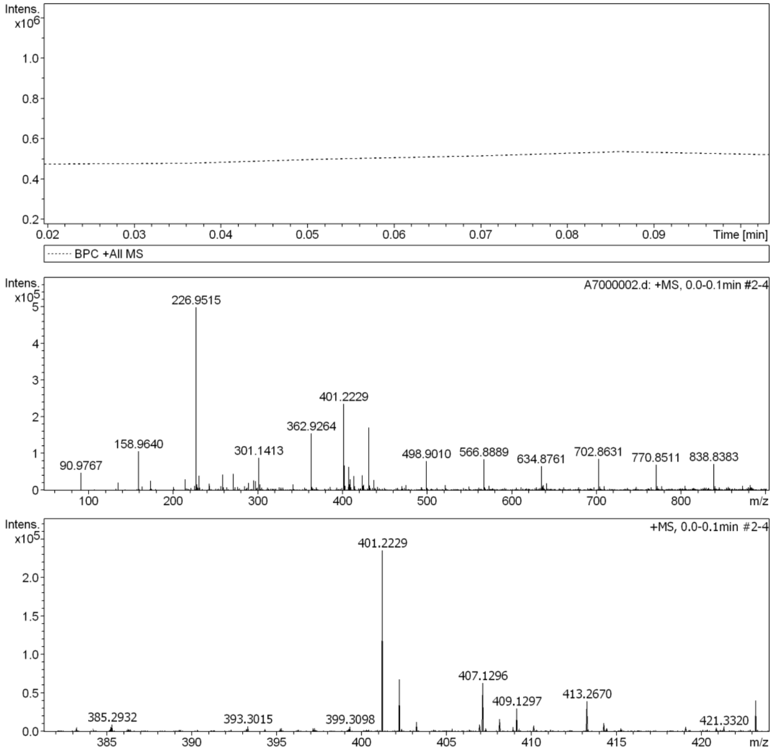

Bruker Compass DataAnalysis 4.1 printed: 10/17/2023 2:26:08 PM by: Demidov Page 1 of 2

Display Report

Meas. m/z	#	Ion Formula	m/z	err [ppm]	mSigma	#	Score	rdb	e⁻ Conf	N-Rule
401.2229	1	C26H29N2O2	401.2224	-1.4	3.9	1	100.00	13.5	even	ok

+MS, 0.0-0.1min #2-4

Bruker Compass DataAnalysis 4.1 printed: 10/17/2023 2:26:08 PM by: Demidov Page 2 of 2

Figure S44: HRMS spectrum of 6,8-di-*tert*-butyl-2-(phenylamino)-3*H*-phenoxazin-3-one (**4a**).

Display Report

Analysis Info

Analysis Name	D:\Data\demidov\FON-mm000001.d	Acquisition Date	7/24/2023 10:52:40 AM
Method	Tune_pos_Standard23.m	Operator	Demidov
Sample Name	1	Instrument	maXis impact 282001.00109
Comment			

Acquisition Parameter

Source Type	ESI	Ion Polarity	Positive	Set Nebulizer	0.3 Bar
Focus	Active	Set Capillary	4000 V	Set Dry Heater	220 °C
Scan Begin	50 m/z	Set End Plate Offset	-500 V	Set Dry Gas	4.0 l/min
Scan End	1111 m/z	Set Collision Cell RF	500.0 Vpp	Set Divert Valve	Source

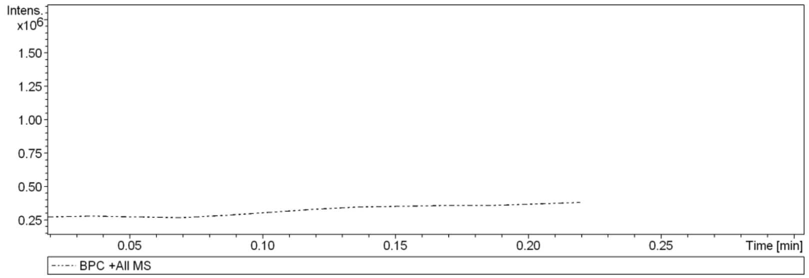

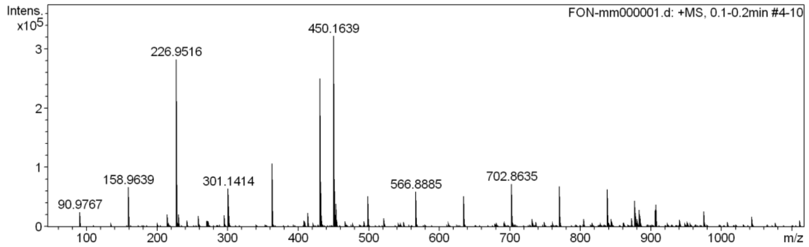

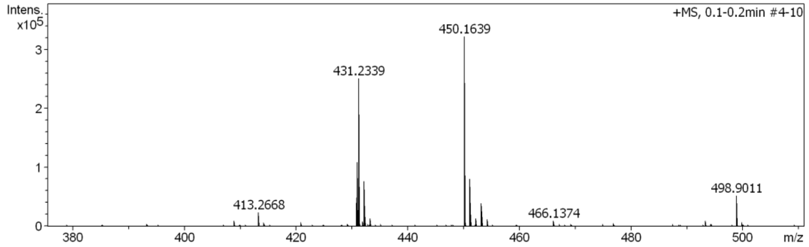

Display Report

Meas. m/z	#	Ion Formula	m/z	err [ppm]	mSigma		Score	rdb	e⁻ Conf	N-Rule
431.2339	1	C27H31N2O3	431.2329	-2.2	0.6	1	100.00	13.5	even	ok
	1	C30H32NaO	431.2345	1.5	12.3	1	100.00	14.5	even	ok
	1	C27H36KO2	431.2347	1.9	31.6	1	100.00	9.5	even	ok
	1	C27H31N2O3	431.2329	-2.2	0.6	1	100.00	13.5	even	ok

+MS, 0.1-0.2min #4-10

Figure S45: HRMS spectrum of 6,8-di-*tert*-butyl-2-((3-methoxyphenyl)amino)-3*H*-phenoxazin-3-one (**4c**).

Display Report

Analysis Info

Analysis Name	D:\Data\demidov\FON-mh000001.d	Acquisition Date	7/24/2023 10:48:10 AM
Method	Tune_pos_Standard23.m	Operator	Demidov
Sample Name	1	Instrument	maXis impact 282001.00109
Comment			

Acquisition Parameter

Source Type	ESI	Ion Polarity	Positive	Set Nebulizer	0.3 Bar
Focus	Active	Set Capillary	4000 V	Set Dry Heater	220 °C
Scan Begin	50 m/z	Set End Plate Offset	-500 V	Set Dry Gas	4.0 l/min
Scan End	1111 m/z	Set Collision Cell RF	500.0 Vpp	Set Divert Valve	Source

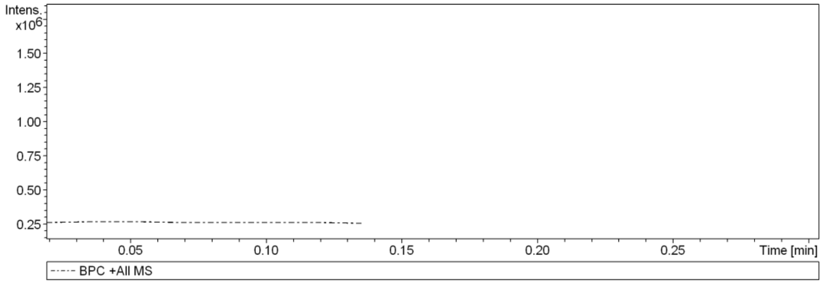

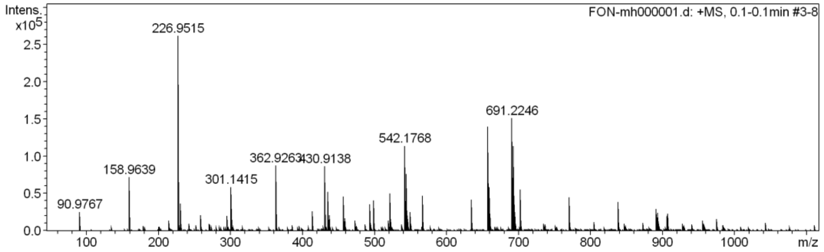

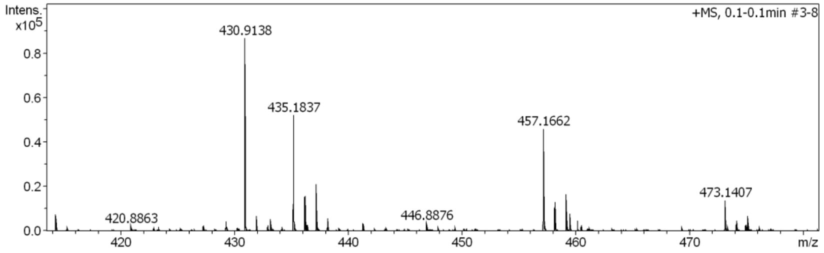

Display Report

Meas. m/z	#	Ion Formula	m/z	err [ppm]	mSigma		Score	rdb	e⁻ Conf	N-Rule
435.1837	1	C26H28ClN2O2	435.1834	-0.7	18.1	1	100.00	13.5	even	ok
	1	C29H29ClNa	435.1850	3.0	18.9	1	100.00	14.5	even	ok
	1	C26H33ClKO	435.1852	3.3	15.7	1	100.00	9.5	even	ok
	1	C26H28ClN2O2	435.1834	-0.7	18.1	1	100.00	13.5	even	ok

+MS, 0.1-0.1min #3-8

Figure S46: HRMS spectrum of 6,8-di-*tert*-butyl-2-((3-chlorophenyl)amino)-3*H*-phenoxazin-3-one (**4d**).

Display Report

Analysis Info

Analysis Name	D:\Data\demidov\FON-1000003.d	Acquisition Date	5/10/2023 10:51:41 AM
Method	Direct_Infusion_50-500_neg.m	Operator	Demidov
Sample Name	1	Instrument	maXis impact 282001.00109
Comment			

Acquisition Parameter

Source Type	ESI	Ion Polarity	Negative	Set Nebulizer	0.4 Bar
Focus	Active	Set Capillary	4500 V	Set Dry Heater	180 °C
Scan Begin	50 m/z	Set End Plate Offset	-500 V	Set Dry Gas	4.0 l/min
Scan End	1200 m/z	Set Collision Cell RF	1600.0 Vpp	Set Divert Valve	Waste

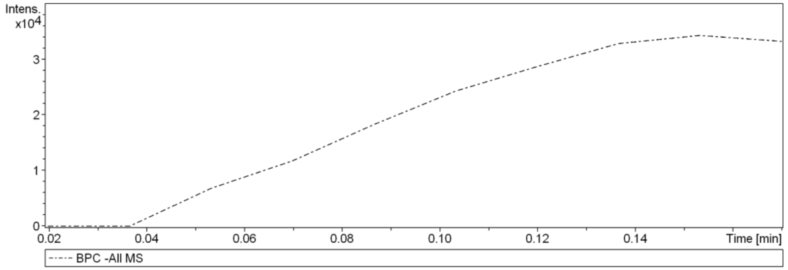

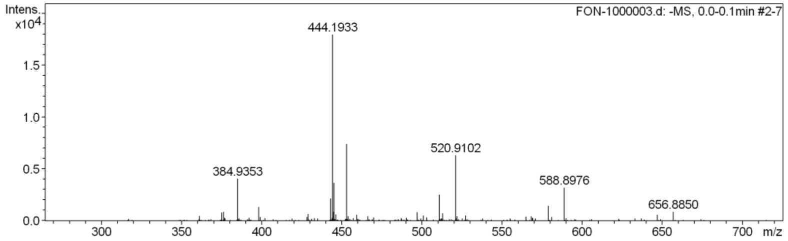

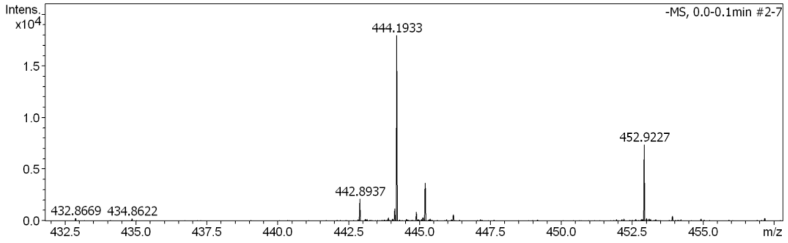

Display Report

Meas. m/z	#	Ion Formula	m/z	err [ppm]	mSigma	mSigma	Score	rdb	e⁻ Conf	N-Rule
444.1933	1	C26H26N3O4	444.1929	-0.9	46.8	46.8	100.00	15.5	even	ok

-MS, 0.0-0.1min #2-7

Figure S47: HRMS spectrum of 6,8-di-*tert*-butyl-2-((4-nitrophenyl)amino)-3*H*-phenoxazin-3-one (**4e**).

Display Report

Analysis Info

Analysis Name	D:\Data\demidov\FON-3000001.d	Acquisition Date	4/28/2023 11:21:27 AM
Method	Direct_Infusion_50-500_2023_2.m	Operator	Demidov
Sample Name	1	Instrument	maXis impact 282001.00109
Comment			

Acquisition Parameter

Source Type	ESI	Ion Polarity	Positive	Set Nebulizer	0.4 Bar		
Focus	Active	Set Capillary	4500 V	Set Dry Heater	180 °C		
Scan Begin	50 m/z	Set End Plate Offset	-500 V	Set Dry Gas	8.0 l/min		
Scan End	900 m/z	Set Collision Cell RF	500.0 Vpp	Set Divert Valve	Waste		

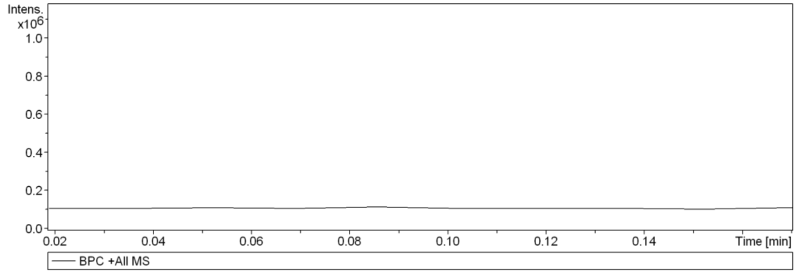

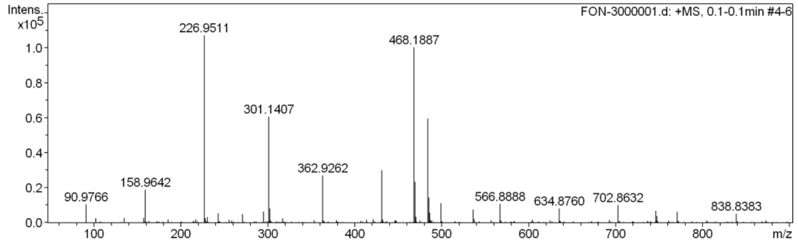

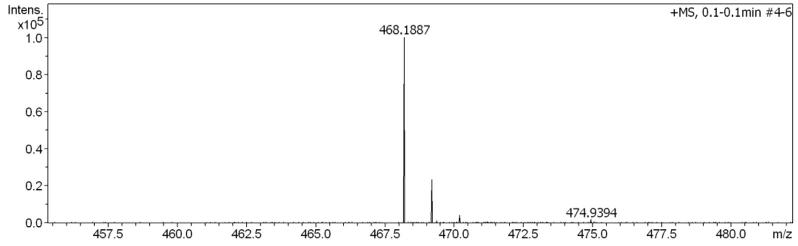

Display Report

Meas. m/z	#	Ion Formula	m/z	err [ppm]	mSigma	#	Score	rdb	e⁻ Conf	N-Rule
468.1887	1	C26H27N3NaO4	468.1894	1.5	32.6	1	100.00	14.5	even	ok

+MS, 0.1-0.1min #4-6

Figure S48: HRMS spectrum of 6,8-di-*tert*-butyl-2-((2-nitrophenyl)amino)-3*H*-phenoxazin-3-one (**4f**).

Display Report

Analysis Info

Acquisition Date 7/24/2023 10:56:23 AM

Analysis Name D:\Data\demidov\FON-pa000001.d
Method Tune_pos_Standard23.m
Sample Name 1
Comment

Operator Demidov
Instrument maXis impact 282001.00109

Acquisition Parameter

Source Type	ESI	Ion Polarity	Positive	Set Nebulizer	0.3 Bar
Focus	Active	Set Capillary	4000 V	Set Dry Heater	220 °C
Scan Begin	50 m/z	Set End Plate Offset	-500 V	Set Dry Gas	4.0 l/min
Scan End	1111 m/z	Set Collision Cell RF	500.0 Vpp	Set Divert Valve	Source

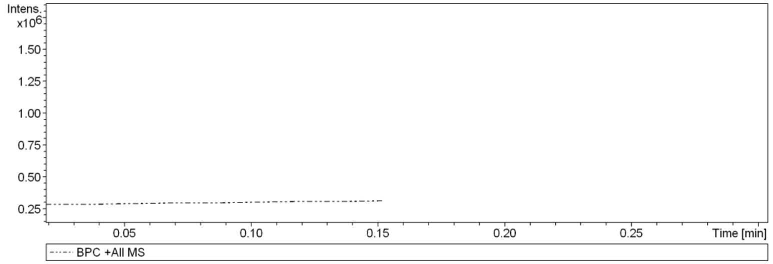

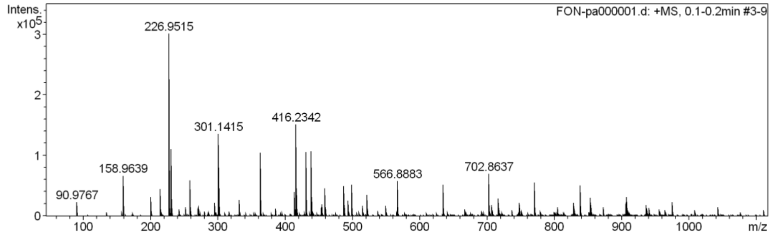

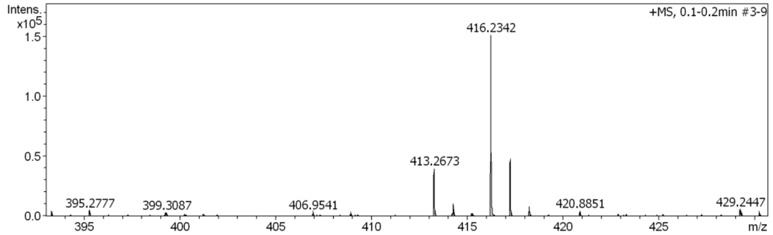

Display Report

Meas. m/z	#	Ion Formula	m/z	err [ppm]	mSigma		Score	rdb	e⁻ Conf	N-Rule
416.2342	1	C26H30N3O2	416.2333	-2.2	6.1	1	100.00	13.5	even	ok

+MS, 0.1-0.2min #3-9

Figure S49: HRMS spectrum of 2-((4-aminophenyl)amino)-6,8-di-*tert*-butyl-3*H*-phenoxazin-3-one (**4g**).

Display Report

Analysis Info

Acquisition Date 5/10/2023 11:06:50 AM

Analysis Name	D:\Data\demidov\FON-4_NEG000001.d		
Method	Direct_Infusion_50-500_neg.m	Operator	Demidov
Sample Name	1	Instrument	maXis impact 282001.00109
Comment			

Acquisition Parameter

Source Type	ESI	Ion Polarity	Negative	Set Nebulizer	0.4 Bar
Focus	Active	Set Capillary	4500 V	Set Dry Heater	180 °C
Scan Begin	50 m/z	Set End Plate Offset	-500 V	Set Dry Gas	4.0 l/min
Scan End	1200 m/z	Set Collision Cell RF	1600.0 Vpp	Set Divert Valve	Waste

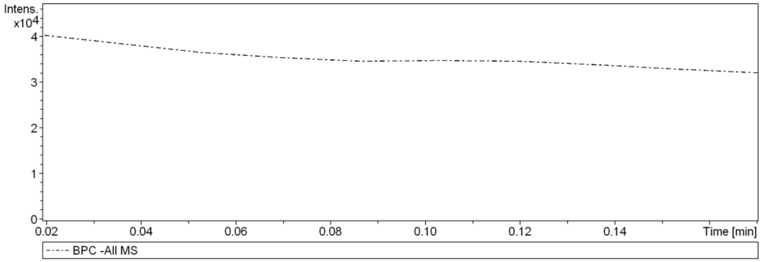

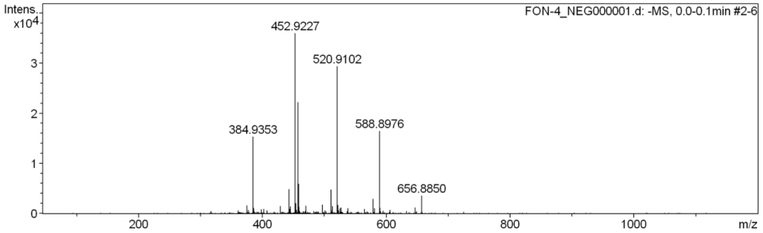

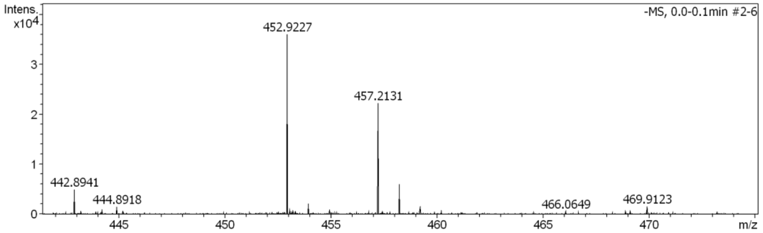

Display Report

Meas. m/z	#	Ion Formula	m/z	err [ppm]	mSigma	mSigma	Score	rdb	e⁻ Conf	N-Rule
457.2131	1	C28H29N2O4	457.2133	0.3	23.6	23.6	100.00	15.5	even	ok

-MS, 0.0-0.1min #2-6

Figure S50: HRMS spectrum of methyl 4-((6,8-di-*tert*-butyl-3-oxo-3*H*-phenoxazin-2-yl)amino)benzoate (**4h**).

Display Report

Analysis Info

Analysis Name	D:\Data\demidov\FDA-0000001.d	Acquisition Date	4/28/2023 11:11:32 AM
Method	Direct_Infusion_50-500_2023_2.m	Operator	Demidov
Sample Name	1	Instrument	maXis impact 282001.00109
Comment			

Acquisition Parameter

Source Type	ESI	Ion Polarity	Positive	Set Nebulizer	0.4 Bar
Focus	Active	Set Capillary	4500 V	Set Dry Heater	180 °C
Scan Begin	50 m/z	Set End Plate Offset	-500 V	Set Dry Gas	8.0 l/min
Scan End	900 m/z	Set Collision Cell RF	500.0 Vpp	Set Divert Valve	Waste

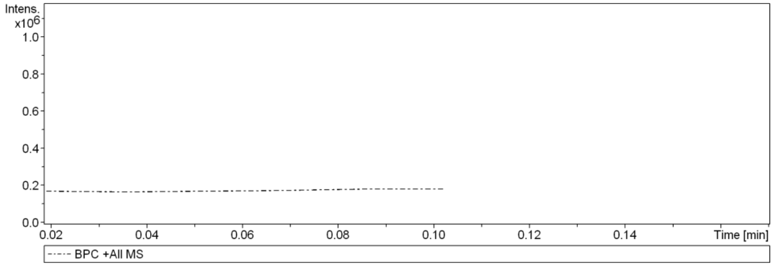

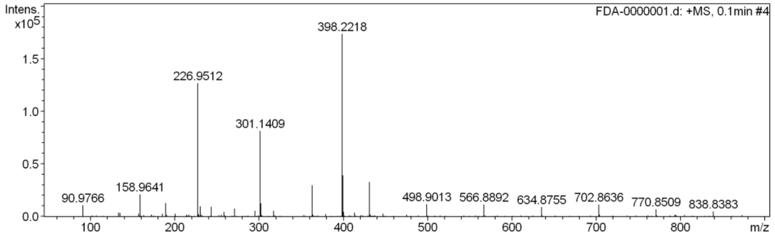

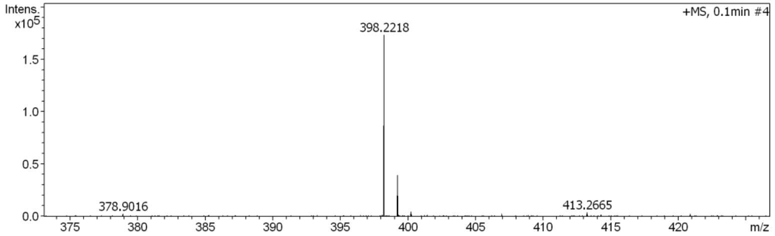

Display Report

Meas. m/z	#	Ion Formula	m/z	err [ppm]	mSigma		Score	rdb	e⁻ Conf	N-Rule
398.2218	1	C26H28N3O	398.2227	2.2	35.8	1	100.00	14.5	even	ok
	1	C26H28N3O	398.2227	2.2	35.8	1	100.00	14.5	even	ok

+MS, 0.1min #4

Figure S51: HRMS spectrum of 2,4-di-*tert*-butyl-14*H*-quinoxalino[2,3-*b*]phenoxazine (**5a**).

Display Report

Analysis Info

Analysis Name	D:\Data\demidov\FDA-DM000001.d	Acquisition Date	4/28/2023 11:14:31 AM
Method	Direct_Infusion_50-500_2023_2.m	Operator	Demidov
Sample Name	1	Instrument	maXis impact 282001.00109
Comment			

Acquisition Parameter

Source Type	ESI	Ion Polarity	Positive	Set Nebulizer	0.4 Bar
Focus	Active	Set Capillary	4500 V	Set Dry Heater	180 °C
Scan Begin	50 m/z	Set End Plate Offset	-500 V	Set Dry Gas	8.0 l/min
Scan End	900 m/z	Set Collision Cell RF	500.0 Vpp	Set Divert Valve	Waste

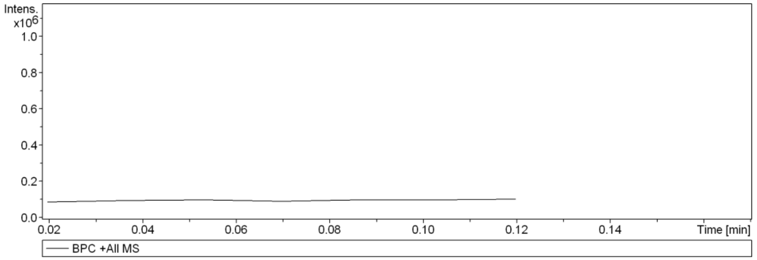

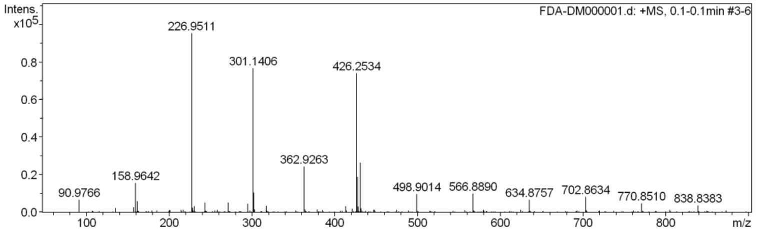

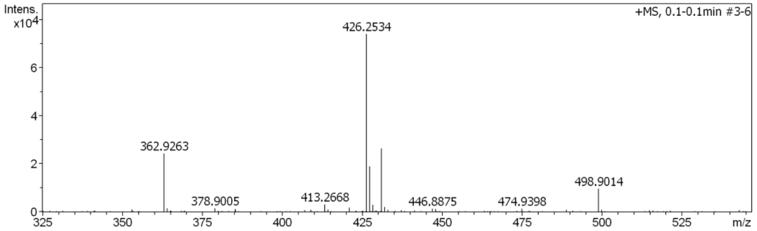

Bruker Compass DataAnalysis 4.1 printed: 4/28/2023 11:16:38 AM by: Demidov Page 1 of 2

Display Report

Meas. m/z	#	Ion Formula	m/z	err [ppm]	mSigma		Score	rdb	e⁻ Conf	N-Rule
426.2534	1	C28H32N3O	426.2540	1.5	31.4	1	100.00	14.5	even	ok
	1	C28H32N3O	426.2540	1.5	31.4	1	100.00	14.5	even	ok

+MS, 0.1-0.1min #3-6

Bruker Compass DataAnalysis 4.1 printed: 4/28/2023 11:16:38 AM by: Demidov Page 2 of 2

Figure S52: HRMS spectrum of 2,4-di-*tert*-butyl-9,10-dimethyl-14*H*-quinoxalino[2,3-*b*]phenoxazine (**5b**).

Display Report

Analysis Info

Analysis Name	D:\Data\demidov\HF3000001.d	Acquisition Date	10/17/2023 2:20:32 PM
Method	Direct_Infusion_50-500_2023_2.m	Operator	Demidov
Sample Name	1	Instrument	maXis impact 282001.00109
Comment			

Acquisition Parameter

Source Type	ESI	Ion Polarity	Positive	Set Nebulizer	0.4 Bar
Focus	Active	Set Capillary	4500 V	Set Dry Heater	180 °C
Scan Begin	50 m/z	Set End Plate Offset	-500 V	Set Dry Gas	4.0 l/min
Scan End	900 m/z	Set Collision Cell RF	500.0 Vpp	Set Divert Valve	Waste

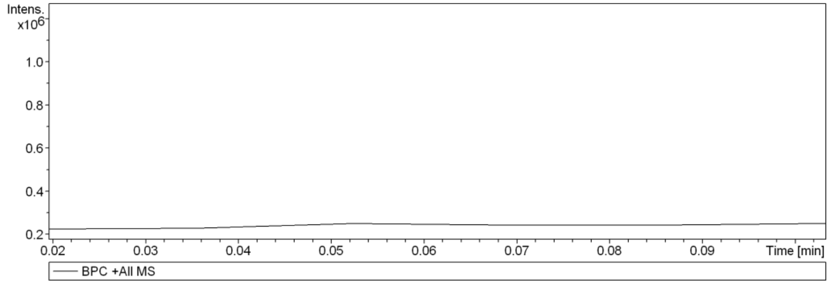

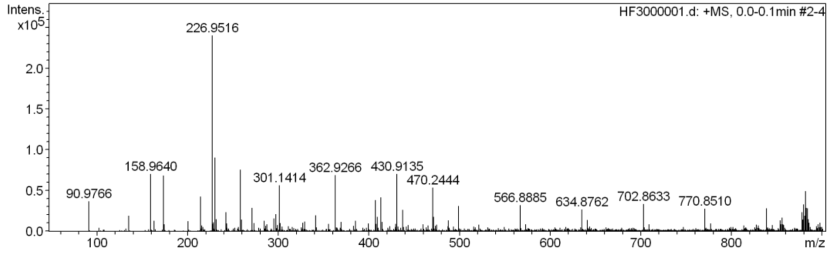

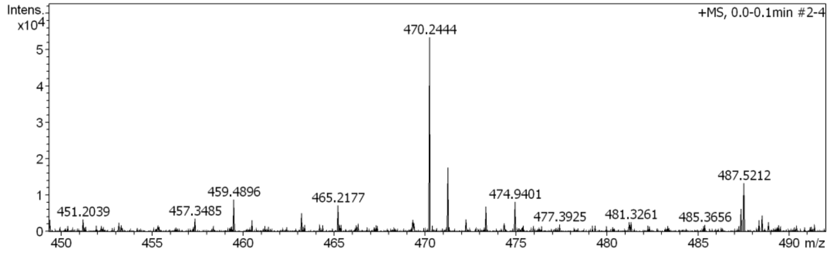

Bruker Compass DataAnalysis 4.1 printed: 10/17/2023 2:22:18 PM by: Demidov Page 1 of 2

Display Report

Meas. m/z	#	Ion Formula	m/z	err [ppm]	mSigma		Score	rdb	e¯ Conf	N-Rule
470.2444	1	C29H32N3O3	470.2438	-1.1	6.4	1	100.00	15.5	even	ok

+MS, 0.0-0.1min #2-4

Bruker Compass DataAnalysis 4.1 printed: 10/17/2023 2:22:18 PM by: Demidov Page 2 of 2

Figure S53: HRMS spectrum of ethyl 2,4-di-*tert*-butyl-14*H*-quinoxalino[2,3-*b*]phenoxazine-10-carboxylate (**5c**).

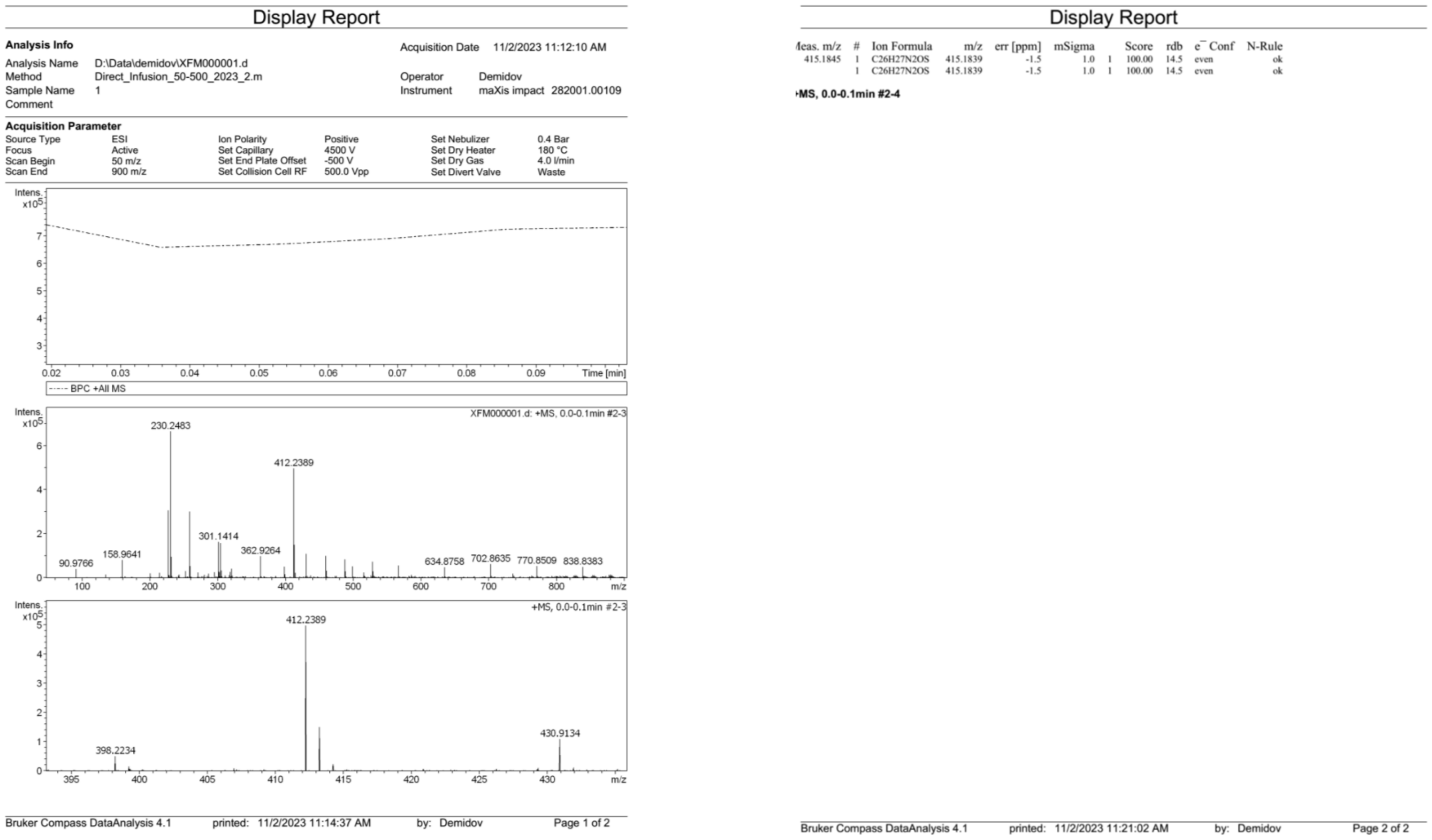

Figure S54: HRMS spectrum of 2,4-di-*tert*-butyl-14-methyl-14*H*-quinoxalino[2,3-*b*]phenoxazine (**6a**).

Display Report

Analysis Info

Analysis Name	D:\Data\demidov\XFN000001.d	Acquisition Date	11/2/2023 11:22:25 AM
Method	Direct_Infusion_50-500_2023_2.m	Operator	Demidov
Sample Name	1	Instrument	maXis impact 282001.00109
Comment			

Acquisition Parameter

Source Type	ESI	Ion Polarity	Positive	Set Nebulizer	0.4 Bar
Focus	Active	Set Capillary	4500 V	Set Dry Heater	180 °C
Scan Begin	50 m/z	Set End Plate Offset	-500 V	Set Dry Gas	4.0 l/min
Scan End	900 m/z	Set Collision Cell RF	500.0 Vpp	Set Divert Valve	Waste

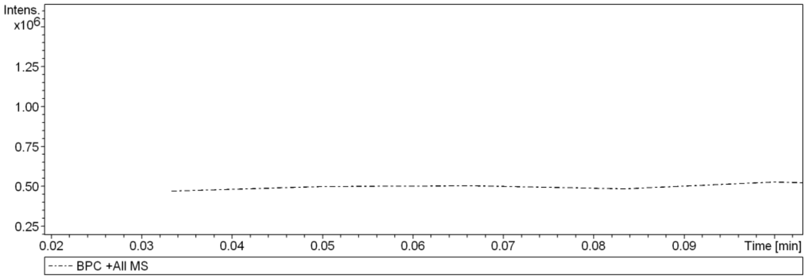

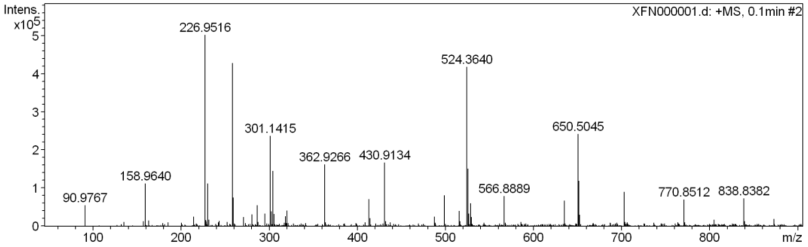

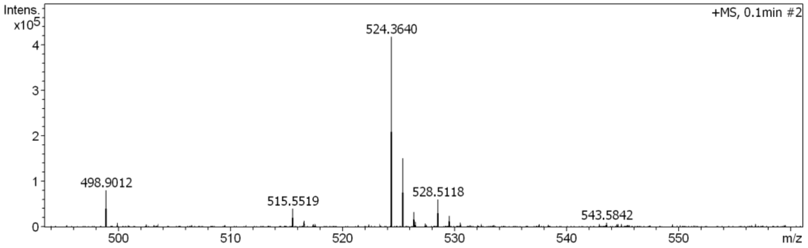

Bruker Compass DataAnalysis 4.1 printed: 11/2/2023 11:25:35 AM by: Demidov Page 1 of 2

Display Report

Meas. m/z	#	Ion Formula	m/z	err [ppm]	mSigma	#	Score	rdb	e⁻ Conf	N-Rule
524.3640	1	C35H46N3O	524.3635	-0.9	16.7	1	100.00	14.5	even	ok
	1	C35H46N3O	524.3635	-0.9	16.7	1	100.00	14.5	even	ok

+MS, 0.1min #2

Bruker Compass DataAnalysis 4.1 printed: 11/2/2023 11:25:35 AM by: Demidov Page 2 of 2

Figure S55: HRMS spectrum of 2,4-di-*tert*-butyl-14-nonyl-14*H*-quinoxalino[2,3-*b*]phenoxazine (**6b**).

Display Report

Analysis Info

Analysis Name	D:\Data\demidov\FT1000002.d	Acquisition Date	11/2/2023 11:18:59 AM
Method	Direct_Infusion_50-500_2023_2.m	Operator	Demidov
Sample Name	1	Instrument	maXis impact 282001.00109
Comment			

Acquisition Parameter

Source Type	ESI	Ion Polarity	Positive	Set Nebulizer	0.4 Bar
Focus	Active	Set Capillary	4500 V	Set Dry Heater	180 °C
Scan Begin	50 m/z	Set End Plate Offset	-500 V	Set Dry Gas	4.0 l/min
Scan End	900 m/z	Set Collision Cell RF	500.0 Vpp	Set Divert Valve	Waste

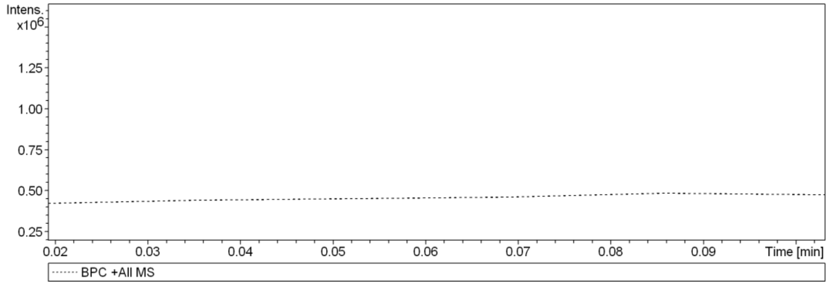

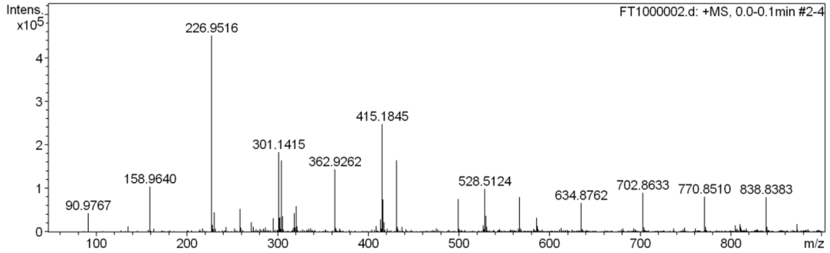

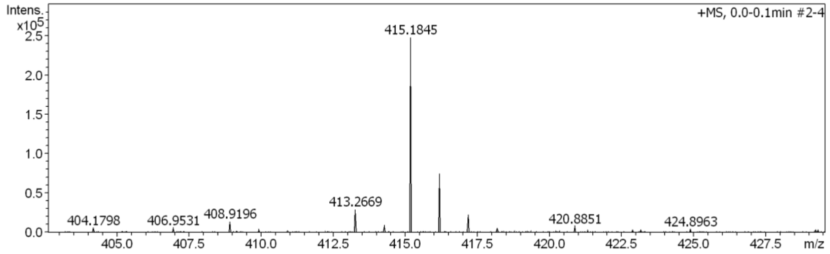

Display Report

Meas. m/z	#	Ion Formula	m/z	err [ppm]	mSigma	#	Score	rdb	e⁻ Conf	N-Rule
415.1845	1	C26H27N2OS	415.1839	-1.5	1.0	1	100.00	14.5	even	ok
	1	C26H27N2OS	415.1839	-1.5	1.0	1	100.00	14.5	even	ok

+MS, 0.0-0.1min #2-4

Figure S56: HRMS spectrum of 2,4-di-*tert*-butylbenzo[5,6][1,4]oxazino[2,3-*b*]phenothiazine (**10c**).

8. Cartesian coordinates of compounds **1**, **7b** (7*H*-tautomer), **7** (12*H*-tautomer), and **7a** (14*H*-tautomer) calculated by the DFT B3LYP/6-311++G(d,p) method.

1

8	-6.085914609	0.070266272	0.000220138
6	-3.432254221	-2.333867993	-0.000050801
1	-3.233269287	-3.399345746	-0.000087314
6	-4.679886954	-1.828604793	0.000047626
6	-4.937481442	-0.368508982	0.000064560
8	-1.435138224	0.806511637	-0.000202675
7	-1.075598268	-1.969048435	-0.000128590
6	-0.164132791	0.290885030	-0.000076202
6	-3.766159244	0.504184208	0.000038630
1	-3.927754100	1.574715074	0.000076731
6	-2.519756848	-0.021159152	-0.000068793
6	-2.273220576	-1.462171773	-0.000099485
6	0.003988409	-1.104431549	-0.000083610
6	0.931818163	1.172884859	0.000014288
6	2.412566175	-0.826291753	0.000030692
6	3.853123174	-1.366529336	0.000049213
6	1.299496866	-1.648703049	-0.000032280
1	1.369106429	-2.727568987	-0.000044451
6	2.189739690	0.567156829	0.000060855
1	3.055412814	1.212815516	0.000114302
6	0.761523633	2.706890857	0.000003704
6	4.593360123	-0.865925012	1.262442288
1	4.091590552	-1.208398474	2.171580972
1	5.618226698	-1.248946151	1.275589167
1	4.647482256	0.224697127	1.300075786
6	2.122183335	3.431759440	-0.000016934
1	2.715907508	3.196796274	-0.887759395
1	1.949107978	4.510770901	-0.000261943
1	2.715728646	3.197151882	0.887937198
6	-0.001477992	3.157169365	-1.269517387
1	-1.005085929	2.736895736	-1.317002050

1	-0.091806961	4.247408380	-1.278447783
1	0.539236379	2.858361494	-2.172101683
6	4.593473896	-0.865722337	-1.262193045
1	4.647592854	0.224905623	-1.299656678
1	5.618345762	-1.248732363	-1.275299707
1	4.091795872	-1.208060329	-2.171433332
6	-0.001469525	3.157253505	1.269497808
1	0.539223679	2.858484263	2.172108033
1	-0.091728643	4.247494636	1.278339831
1	-1.005092808	2.737021680	1.317009458
6	3.891731417	-2.905676819	-0.000075143
1	3.408376156	-3.325498002	-0.886372950
1	4.931019659	-3.244498958	-0.000051330
1	3.408290429	-3.325646172	0.886106245
1	-5.557087675	-2.465251591	0.000115890

7b

8	-0.464808000	1.056262000	-0.000042000
7	-0.494079000	-1.740064000	-0.000063000
6	-1.682062000	0.389788000	-0.000032000
6	1.856869000	1.103520000	0.000006000
1	1.819862000	2.187196000	0.000038000
6	0.685142000	0.375352000	-0.000032000
6	0.665656000	-1.077008000	-0.000057000
6	-1.653118000	-1.018856000	-0.000043000
6	-2.867507000	1.142053000	-0.000011000
6	-4.089862000	-1.013205000	0.000007000
6	-5.454457000	-1.727334000	0.000033000
6	-2.890149000	-1.700621000	-0.000028000
1	-2.836307000	-2.780223000	-0.000040000
6	-4.049297000	0.399724000	0.000009000
1	-4.986930000	0.934919000	0.000015000
6	-2.871831000	2.686160000	-0.000001000
6	-6.250559000	-1.320432000	1.262091000
1	-5.711893000	-1.603912000	2.170506000

1	-7.223583000	-1.821396000	1.273535000
1	-6.430682000	-0.243614000	1.301924000
6	-4.304415000	3.255975000	0.000027000
1	-4.867370000	2.954078000	-0.887047000
1	-4.252139000	4.347843000	0.000072000
1	-4.867365000	2.954007000	0.887080000
6	-2.166206000	3.222127000	-1.269581000
1	-1.122231000	2.914957000	-1.320605000
1	-2.198562000	4.316203000	-1.279286000
1	-2.670950000	2.863535000	-2.171030000
6	-6.250649000	-1.320366000	-1.261944000
1	-6.430772000	-0.243546000	-1.301706000
1	-7.223674000	-1.821330000	-1.273346000
1	-5.712045000	-1.603798000	-2.170410000
6	-2.166161000	3.222102000	1.269561000
1	-2.670902000	2.863527000	2.171019000
1	-2.198478000	4.316179000	1.279266000
1	-1.122197000	2.914892000	1.320566000
6	-5.307660000	-3.259363000	-0.000012000
1	-4.775455000	-3.616360000	-0.885616000
1	-6.298031000	-3.722940000	-0.000014000
1	-4.775434000	-3.616414000	0.885558000
7	4.308906000	-1.692847000	-0.000009000
7	4.251536000	1.076021000	0.000008000
6	5.457221000	-0.981621000	0.000011000
6	1.910316000	-1.731357000	-0.000087000
1	1.925721000	-2.812873000	-0.000093000
6	3.127014000	-1.043951000	-0.000034000
6	3.076483000	0.415714000	-0.000012000
6	5.485314000	0.442824000	0.000020000
6	6.712957000	-1.643567000	0.000028000
6	7.887638000	0.479425000	0.000044000
6	6.683289000	1.163453000	0.000028000
1	6.661802000	2.249414000	0.000020000
6	7.893029000	-0.929566000	0.000045000
1	8.839523000	-1.458122000	0.000058000

1	8.821244000	1.028385000	0.000062000
1	6.703729000	-2.726633000	0.000021000
1	4.235079000	2.088361000	0.000011000

7

8	-0.480028000	1.099783000	0.000049000
7	-0.471477000	-1.707225000	0.000052000
6	-1.680494000	0.415672000	0.000031000
6	1.859125000	1.123684000	-0.000101000
1	1.859997000	2.205769000	-0.000088000
6	0.695781000	0.421193000	-0.000038000
6	0.656634000	-1.038006000	-0.000008000
6	-1.652983000	-0.988157000	0.000052000
6	-2.878798000	1.148812000	0.000005000
6	-4.083933000	-1.018054000	0.000031000
6	-5.439483000	-1.749080000	0.000042000
6	-2.870817000	-1.688865000	0.000050000
1	-2.801720000	-2.767817000	0.000064000
6	-4.052857000	0.390463000	0.000005000
1	-4.995234000	0.918125000	-0.000018000
6	-2.903525000	2.692626000	-0.000020000
6	-6.241269000	-1.352985000	1.261792000
1	-5.699541000	-1.629777000	2.170466000
1	-7.208408000	-1.865320000	1.272922000
1	-6.434052000	-0.278359000	1.301564000
6	-4.343375000	3.243680000	-0.000083000
1	-4.902877000	2.935357000	-0.887420000
1	-4.304721000	4.336035000	-0.000089000
1	-4.902951000	2.935373000	0.887212000
6	-2.203537000	3.236543000	-1.269414000
1	-1.156774000	2.940071000	-1.319157000
1	-2.247140000	4.330145000	-1.277332000
1	-2.705463000	2.874288000	-2.171229000
6	-6.241252000	-1.353056000	-1.261742000
1	-6.434041000	-0.278434000	-1.301573000
1	-7.208386000	-1.865399000	-1.272856000

1	-5.699507000	-1.629895000	-2.170392000
6	-2.203628000	3.236581000	1.269407000
1	-2.705658000	2.874395000	2.171194000
1	-2.247185000	4.330184000	1.277263000
1	-1.156885000	2.940062000	1.319266000
6	-5.271781000	-3.279063000	0.000084000
1	-4.734946000	-3.628971000	-0.885720000
1	-6.255638000	-3.756257000	0.000095000
1	-4.734949000	-3.628922000	0.885908000
7	4.324949000	-1.633719000	-0.000342000
7	4.235963000	1.142199000	-0.000068000
6	5.519358000	-0.936616000	-0.000087000
6	1.914543000	-1.714944000	-0.000061000
1	1.893764000	-2.799008000	-0.000066000
6	3.093754000	-1.020970000	-0.000140000
6	3.122536000	0.447319000	-0.000115000
6	5.442839000	0.478131000	-0.000008000
6	6.758119000	-1.585458000	-0.000002000
6	7.869726000	0.566430000	0.000242000
6	6.642740000	1.210953000	0.000157000
1	6.569370000	2.291848000	0.000215000
6	7.925941000	-0.833899000	0.000170000
1	8.884913000	-1.338732000	0.000244000
1	8.786173000	1.144297000	0.000371000
1	6.799630000	-2.670238000	-0.000070000
1	4.356242000	-2.643466000	-0.000150000

7a

8	-0.440561000	1.072076000	-0.004362000
7	-0.461801000	-1.687132000	-0.004956000
6	-1.656000000	0.399397000	-0.002257000
6	1.904136000	1.106772000	-0.001386000
1	1.882199000	2.188842000	-0.001062000
6	0.747729000	0.389810000	-0.002803000
6	0.761669000	-1.052090000	-0.003308000

6	-1.674506000	-0.996200000	-0.002727000
6	-2.845133000	1.145638000	-0.000275000
6	-4.096040000	-0.986191000	0.000630000
6	-5.461072000	-1.699746000	0.002126000
6	-2.890188000	-1.681512000	-0.001208000
1	-2.864744000	-2.764150000	-0.001597000
6	-4.039499000	0.414833000	0.001053000
1	-4.969700000	0.962518000	0.002504000
6	-2.841198000	2.689891000	0.000224000
6	-6.255427000	-1.292063000	1.264906000
1	-5.717141000	-1.575965000	2.173452000
1	-7.229836000	-1.790202000	1.277018000
1	-6.431532000	-0.214879000	1.303791000
6	-4.270253000	3.268928000	0.002543000
1	-4.837353000	2.972995000	-0.884136000
1	-4.208590000	4.360112000	0.002574000
1	-4.834542000	2.972773000	0.890939000
6	-2.133969000	3.220462000	-1.270831000
1	-1.094041000	2.902241000	-1.324242000
1	-2.155650000	4.314624000	-1.276757000
1	-2.646911000	2.870307000	-2.171276000
6	-6.258235000	-1.291799000	-1.258800000
1	-6.434218000	-0.214577000	-1.297158000
1	-7.232760000	-1.789768000	-1.268777000
1	-5.722054000	-1.575690000	-2.168596000
6	-2.130178000	3.219646000	1.269498000
1	-2.640106000	2.868521000	2.171272000
1	-2.152247000	4.313794000	1.276433000
1	-1.089952000	2.901816000	1.319321000
6	-5.318372000	-3.232533000	0.001808000
1	-4.790837000	-3.592386000	-0.886074000
1	-6.309581000	-3.693620000	0.002932000
1	-4.788726000	-3.592558000	0.888363000
7	4.345272000	-1.681206000	-0.000547000
7	4.288708000	1.157984000	0.000590000
6	5.481134000	-0.957918000	0.000619000

6	1.959906000	-1.719047000	-0.002397000
1	1.997436000	-2.802539000	-0.002753000
6	3.193557000	-1.008480000	-0.001159000
6	3.168105000	0.440634000	-0.000595000
6	5.454016000	0.480916000	0.001219000
6	6.739956000	-1.623091000	0.001267000
6	7.876543000	0.522268000	0.003088000
6	6.683663000	1.197193000	0.002484000
1	6.637039000	2.279869000	0.002933000
6	7.904099000	-0.899839000	0.002460000
1	8.859827000	-1.411934000	0.002938000
1	8.811108000	1.071811000	0.004054000
1	6.738309000	-2.706754000	0.000776000
1	-0.482823000	-2.694588000	-0.004656000

7Hmod

8	-2.358639000	1.339421000	0.000001000
7	-2.401241000	-1.475812000	-0.000024000
6	-3.565892000	0.662517000	-0.000009000
6	-0.038806000	1.368303000	0.000013000
1	-0.073626000	2.452041000	0.000023000
6	-1.213275000	0.647349000	0.000001000
6	-1.241614000	-0.809341000	-0.000013000
6	-3.554476000	-0.748197000	-0.000022000
6	-4.729935000	1.415865000	-0.000007000
6	-5.986388000	-0.650136000	-0.000030000
6	-4.815316000	-1.387905000	-0.000032000
1	-4.825793000	-2.471203000	-0.000042000
6	-5.953030000	0.753029000	-0.000017000
1	-6.874449000	1.322334000	-0.000016000
7	2.399058000	-1.438970000	-0.000004000
7	2.355809000	1.329311000	0.000023000
6	3.550575000	-0.734017000	0.000008000
6	0.000829000	-1.466502000	-0.000015000
1	0.011999000	-2.548043000	-0.000025000
6	1.220662000	-0.784478000	-0.000003000

6	1.177952000	0.675157000	0.000011000
6	3.585523000	0.690542000	0.000022000
6	4.802790000	-1.403235000	0.000007000
6	5.987451000	0.714191000	0.000032000
6	4.787755000	1.405055000	0.000033000
1	4.772342000	2.491023000	0.000044000
6	5.985924000	-0.695439000	0.000019000
1	6.929877000	-1.228375000	0.000018000
1	6.924127000	1.257889000	0.000041000
1	4.787545000	-2.486198000	-0.000003000
1	2.344128000	2.341902000	0.000032000
1	-4.665082000	2.497575000	0.000003000
1	-6.941710000	-1.162565000	-0.000038000

12Hmod

8	2.379513000	-1.377400000	0.000023000
7	2.381743000	1.448286000	-0.000025000
6	3.567942000	-0.683568000	-0.000003000
6	0.041403000	-1.383348000	-0.000002000
1	0.038279000	-2.465430000	0.000019000
6	1.207473000	-0.688019000	-0.000002000
6	1.254004000	0.775286000	-0.000021000
6	3.557826000	0.722994000	-0.000022000
6	4.746974000	-1.415524000	-0.000001000
6	5.985065000	0.664329000	-0.000039000
6	4.798737000	1.383758000	-0.000040000
1	4.790600000	2.467238000	-0.000055000
6	5.961879000	-0.735167000	-0.000020000
1	6.889049000	-1.295821000	-0.000020000
7	-2.412023000	1.384341000	-0.000096000
7	-2.335351000	-1.391202000	0.000022000
6	-3.609127000	0.692828000	-0.000011000
6	-0.001569000	1.455207000	-0.000037000
1	0.023689000	2.539108000	-0.000056000
6	-1.184128000	0.766423000	-0.000042000

6	-1.219227000	-0.701453000	-0.000013000
6	-3.538933000	-0.722353000	0.000034000
6	-4.845252000	1.347168000	0.000004000
6	-5.965566000	-0.800747000	0.000112000
6	-4.741916000	-1.450607000	0.000095000
1	-4.672940000	-2.531754000	0.000129000
6	-6.015572000	0.600254000	0.000068000
1	-6.972491000	1.108922000	0.000083000
1	-6.884659000	-1.374320000	0.000161000
1	-4.882394000	2.432052000	-0.000034000
1	-2.438881000	2.394356000	-0.000039000
1	6.933893000	1.188037000	-0.000053000
1	4.699090000	-2.497922000	0.000014000

14Hmod

8	2.328274000	-1.365100000	-0.003095000
7	2.367159000	1.412658000	-0.002794000
6	3.530563000	-0.685045000	-0.000739000
6	-0.016361000	-1.378116000	-0.001247000
1	0.001548000	-2.460263000	-0.001280000
6	1.144448000	-0.669772000	-0.001940000
6	1.141006000	0.774155000	-0.001883000
6	3.571883000	0.714421000	-0.000625000
6	4.697655000	-1.432843000	0.001096000
6	5.988338000	0.604897000	0.003144000
6	4.813470000	1.353211000	0.001384000
1	4.852122000	2.437636000	0.001491000
6	5.934213000	-0.787411000	0.003012000
1	6.845686000	-1.372158000	0.004410000
7	-2.438455000	1.425623000	-0.000100000
7	-2.401483000	-1.413877000	-0.000124000
6	-3.578950000	0.710439000	0.000410000
6	-0.053165000	1.447137000	-0.001182000
1	-0.084491000	2.530833000	-0.001129000
6	-1.291688000	0.744506000	-0.000624000

6	-1.275797000	-0.704010000	-0.000638000
6	-3.561495000	-0.728953000	0.000402000
6	-4.833220000	1.384482000	0.000973000
6	-5.983976000	-0.753548000	0.001484000
6	-4.796244000	-1.436921000	0.000957000
1	-4.757102000	-2.519852000	0.000942000
6	-6.001871000	0.669045000	0.001492000
1	-6.954176000	1.187422000	0.001916000
1	-6.922395000	-1.296439000	0.001904000
1	-4.824191000	2.468073000	0.000969000
1	2.387475000	2.420170000	-0.001813000
1	4.621206000	-2.513383000	0.000946000
1	6.944730000	1.113876000	0.004659000
